# Direct and selective pharmacological disruption of the YAP–TEAD interface by IAG933 inhibits Hippo-dependent and RAS–MAPK-altered cancers

**DOI:** 10.1038/s43018-024-00754-9

**Published:** 2024-04-02

**Authors:** Emilie A. Chapeau, Laurent Sansregret, Giorgio G. Galli, Patrick Chène, Markus Wartmann, Thanos P. Mourikis, Patricia Jaaks, Sabrina Baltschukat, Ines A. M. Barbosa, Daniel Bauer, Saskia M. Brachmann, Clara Delaunay, Claire Estadieu, Jason E. Faris, Pascal Furet, Stefanie Harlfinger, Andreas Hueber, Eloísa Jiménez Núñez, David P. Kodack, Emeline Mandon, Typhaine Martin, Yannick Mesrouze, Vincent Romanet, Clemens Scheufler, Holger Sellner, Christelle Stamm, Dario Sterker, Luca Tordella, Francesco Hofmann, Nicolas Soldermann, Tobias Schmelzle

**Affiliations:** 1 Novartis BioMedical Research, Basel, Switzerland; 2 Novartis BioMedical Research, Cambridge, MA USA; 3 Present Address: AstraZeneca, Oncology R&D, Cambridge, UK; 4 Present Address: Pierre Fabre Group, R&D Medical Care, Toulouse, France

**Keywords:** Cancer, Cancer therapy

## Abstract

The YAP–TEAD protein–protein interaction mediates YAP oncogenic functions downstream of the Hippo pathway. To date, available YAP–TEAD pharmacologic agents bind into the lipid pocket of TEAD, targeting the interaction indirectly via allosteric changes. However, the consequences of a direct pharmacological disruption of the interface between YAP and TEADs remain largely unexplored. Here, we present IAG933 and its analogs as potent first-in-class and selective disruptors of the YAP–TEAD protein–protein interaction with suitable properties to enter clinical trials. Pharmacologic abrogation of the interaction with all four TEAD paralogs resulted in YAP eviction from chromatin and reduced Hippo-mediated transcription and induction of cell death. In vivo, deep tumor regression was observed in Hippo-driven mesothelioma xenografts at tolerated doses in animal models as well as in Hippo-altered cancer models outside mesothelioma. Importantly this also extended to larger tumor indications, such as lung, pancreatic and colorectal cancer, in combination with RTK, KRAS-mutant selective and MAPK inhibitors, leading to more efficacious and durable responses. Clinical evaluation of IAG933 is underway.

## Abstract (web summary)

Chapeau et al. develop a nonallosteric inhibitor of the interaction between YAP and all four TEAD proteins. Treatment with the inhibitor, either as monotherapy or in combination with other treatment modalities, leads to induction of cell death in several in vivo cancer models.

## Main

The four highly conserved paralogs of the TEA/ATSS domain transcription factor (TEAD1–TEAD4) are the most distal effectors of the Hippo signaling pathway that regulate cell growth, tissue homeostasis and embryonic development^1,2^. Inhibition of the Hippo pathway and subsequent increased TEAD transcriptional activity promote tumorigenesis or resistance to therapies in a wide variety of cancers, including malignant mesothelioma, non-small cell lung cancer (NSCLC), pancreatic ductal adenocarcinoma (PDAC) and colorectal cancer (CRC)^3–9^. These remain challenging cancers to treat, with limited targeted therapy options available. Although systemic chemotherapy and emerging KRAS-targeting therapies in NSCLC, PDAC and CRC as well as immunotherapy in NSCLC offer some benefit, they often fail to achieve durable responses, underscoring the need for alternative therapeutic modalities effective as a single agent or combination partner^10–14^.

TEAD transcriptional activity is dependent on protein–protein interactions (PPIs) with cofactors, of which yes-associated protein (YAP) and its paralog transcriptional coactivator with PDZ-binding motif (WWTR1/TAZ) are the two most important coactivators, and vestigial-like family member 4 (VGLL4) is a prominent co-repressor. The YAP/TAZ–TEAD complex has recently become a druggable oncology target, with the first inhibitors being a variety of allosteric binders of the TEAD lipid pocket (LP)^15–21^, whose binding prevents post-translational autopalmitoylation of a conserved internal cysteine that is essential for TEAD maturation and function^22,23^. Although these TEAD binders showed promising results in preclinical studies^17,21^, to date, the consequences of a direct pharmacological disruption of the YAP–TEAD interface remain unexplored.

We have recently described the chemical discovery and optimization path of a unique class of nonallosteric dihydrobenzofuran-based YAP–TEAD PPI inhibitors (YTPs) with high-affinity binding to the TEAD Ω-loop pocket that mediates the YAP/TAZ–TEAD PPI^24,25^. Unlike the LP-binding compounds, these molecules directly prevent complex formation between YAP/TAZ and TEADs by competition at the binding site. Here, we report NVP-IAG933 (hereafter IAG933), an advanced YTP currently under phase 1 clinical investigation (NCT04857372), which was developed from the lead compounds by structure- and compound property-based optimizations to improve potency, pharmacokinetics (PKs) and preclinical safety (manuscript in preparation). Herein, we describe the biological and preclinical characterization of IAG933 and its close analogs, their monotherapy activity in specific Hippo-dependent cancers and in combination with receptor tyrosine kinases (RTKs), KRAS, BRAF, MEK or ERK inhibitors in a broad range of other cancer models.

## Results

### IAG933 binds to the YAP interface of all TEAD paralogs

**Fig. 1:**
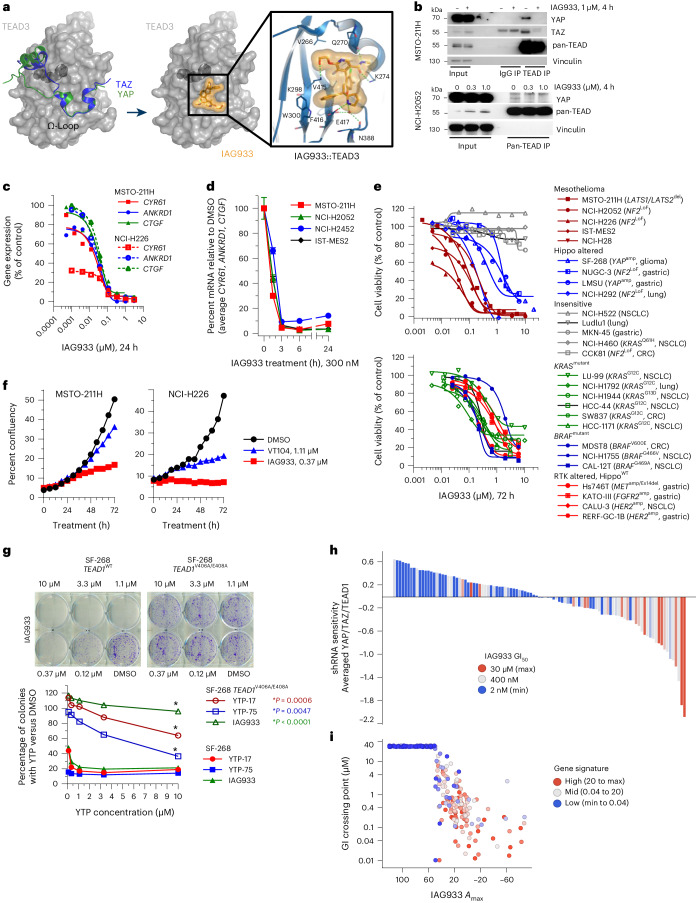
Selective target modulation by YTPs in cellular systems. **a**, Left, YAP and TAZ proteins mapped on the TEAD3 surface, as a result of structural alignments with the complex structures (PDB codes 5GN0, 5OAQ). The TEAD3 surface is shown in gray with the bound myristate in the buried LP drawn in dark gray. Middle/right, TEAD3–IAG933 cocrystal structure. Bound inhibitor is shown as a stick model with its surface in orange. IAG933 binds within the TEAD Ω-loop pocket to prevent coactivator binding by steric hindrance. The main hydrogen bonds and the salt bridge between protein, inhibitor and water are indicated with dotted green lines. **b**, Coimmunoprecipitation after a 4-h incubation of NCI-H2052 or MSTO-211H cells with DMSO (0 or –) or the indicated concentrations of IAG933 shows compound-induced inhibition of YAP and TAZ binding to TEAD isoforms. The blots are representative of two individual experiments; IP, immunoprecipitation. **c**, Dose-dependent inhibition of TEAD target gene expression in MSTO-211H and NCI-H226 cells treated for 24 h with IAG933. IC_50_ values are between 11 and 26 nM. **d**, TEAD target gene inhibition kinetics (mean ± s.e.m., *n* = 4 of the three genes combined) in four mesothelioma cell lines treated with 300 nM IAG933. **e**, Antiproliferative activity of IAG933 (72-h treatment) in a panel of mesothelioma, Hippo-altered, non-Hippo-mutated or insensitive cell lines. The GI_50_ in MSTO-211H cells was 73 nM. The results from one experiment or the mean of two experiments is shown; amp, amplification; Ex, exon; LoF, loss of function; WT, wild type. **f**, Real-time live-cell assessments of MSTO-211H and NCI-H226 cells treated with IAG933 or VT104; data show the mean of *n* = 2 replicates. **g**, Dose-dependent rescue of YTP activity in a CRISPR knock-in *TEAD1*^V406A/E408A^-mutant YTP-resistant clone of (YAP-amplified) SF-268 glioma cells. These TEAD1 residues correspond to V415 and E417 of the TEAD3 protein. Two-tailed paired *t*-test *P* values are included in the graph. **h**, Correlation of pharmacological and genetic sensitivity profiles in 103 cancer cell lines. Each bar represents one cell line. The *y* axis shows cell survival values of averaged shRNA drop-out profiles for YAP, TAZ (WWTR1) and TEAD1. Bar colors stratify GI_50_ for IAG933 (blue, maximum survival/refractory; red, minimum survival/sensitive). Data were analyzed by two-tailed Spearman correlation test between shRNA sensitivity and GI_50_; *P* < 0.0001. **i**, In vitro pharmacological sensitivity of 283 cancer cell lines to IAG933 (GI_50_ versus maximal response (*A*_max_)). Color stratifies geometric mean expression of TEAD target genes *CCN1*, *CCN2*, *ANKRD1* and *AMOTL2*. Data were analyzed by two-tailed Spearman correlation test between gene signature and *A*_max_ (*P* < 0.0001) or between gene signature and GI_50_ (*P* < 0.0001). Source data

**Extended Data Fig. 1:**
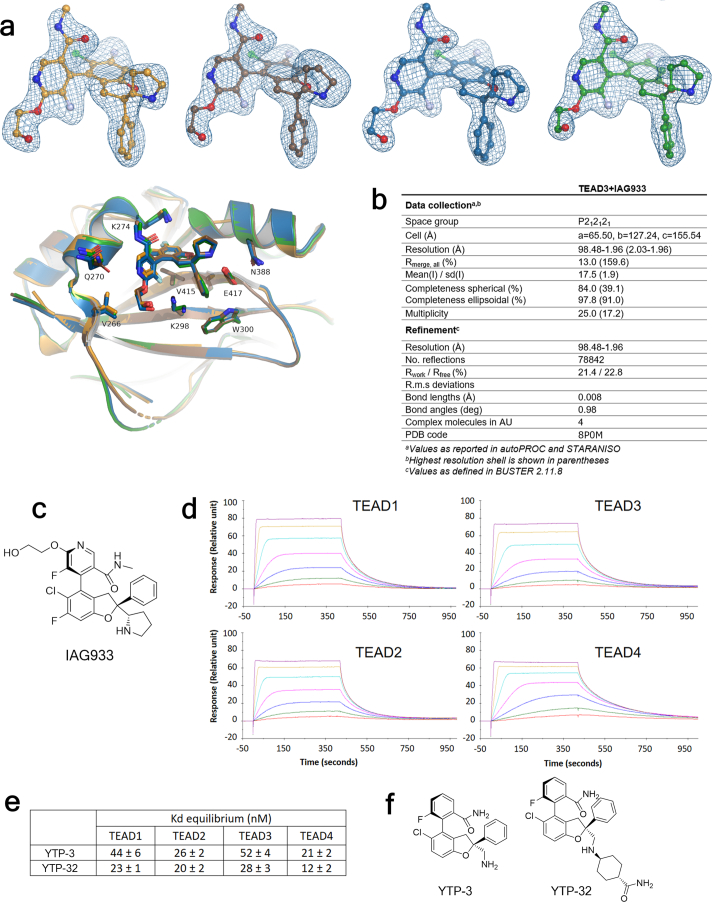
Crystallographic and biochemical characterization of dihydrobenzofuran YTP compounds. **a**, The initial unbiased difference electron density map contoured at 3.5σ within the Ω-loop pocket for all four protein chains within the asymmetric unit. The final refined inhibitor models are superimposed as ball-and-stick representation in yellow for protein chain A, in brown for protein chain B, in blue for protein chain C and in green for protein chain D. All four complexes from the asymmetric unit superimpose well, especially around the binding site of IAG933 (bottom panel, residues of TEAD3 are labelled). Only subtle conformational changes for a few side chains are observed. **b**, Crystallographic data collection and refinement statistics. **c**, IAG933 structure represented in 2D. **d,e**, Surface plasmon resonance. The four N-biotinylated avi-tagged TEAD proteins were immobilized on sensor chips and the binding of YTPs was measured at 298°K. **d**, Representative sensorgrams are shown for YTP-32. The data were globally fitted with a 1:1 interaction model to determine the dissociation constants (K_d_) measured at equilibrium. **e**, The SPR values are represented as the mean ± SD of n = 2 experiments. **f**, 2D chemical structures of the two compounds used in the SPR assay. Source data

**Extended Data Fig. 2:**
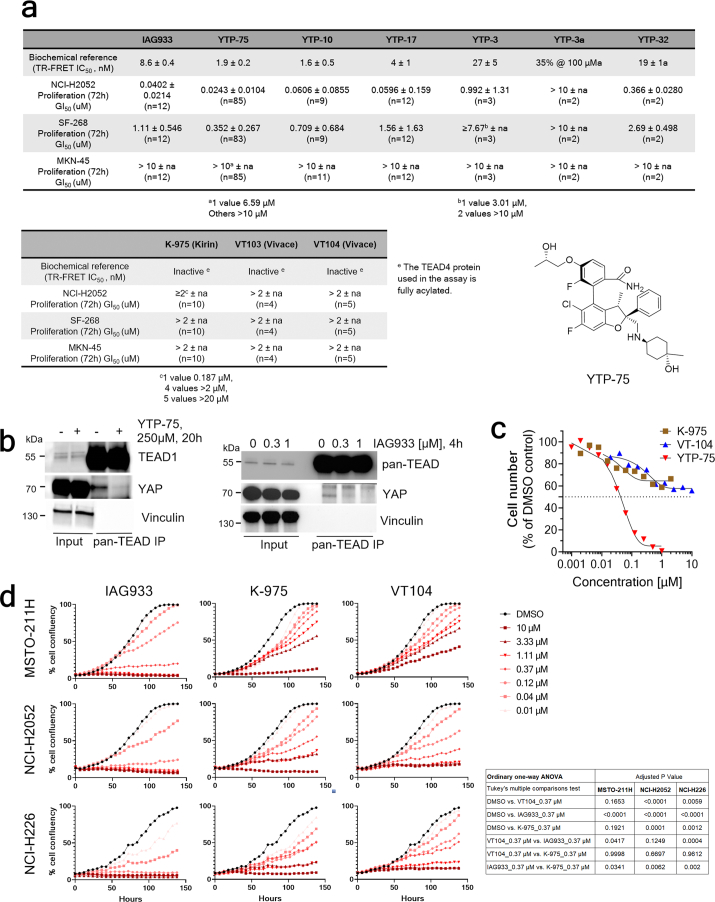
Biochemical and cellular activities of YTPs and allosteric TEAD inhibitors. **a**, Results from biochemical and 72 h cell proliferation assays. Biochemical IC_50_’s have been obtained from a TR-FRET assay. Values represent the mean ± SD of n ≥ 2 experiments as indicated in the table. The values for YTP-3, YTP-3a, YTP-10 and YTP-17 were previously reported^24,25^. All the compounds targeting the TEAD lipid-pocket are inactive in TR-FRET because the TEAD4 protein used in this assay is fully acylated. Resazurin 72 h cell proliferation assays were performed in three cell lines for YTP compounds and indicated lipid-pocket binders. SF-268 (MCAT engineered line) is a YAP-amplified, TEAD-dependent glioma cell line, NCI-H2052 (MCAT engineered line) is a NF2-mutant, highly TEAD-dependent mesothelioma cell line, and MKN-45 is a YAP-deleted and TEAD-insensitive gastric cell line (negative control)^25^. GI_50_’s are represented as geometric mean and SD. **b**, Coimmunoprecipitation with MSTO-211H cell lysate shows dose-dependent YAP-TEADs disruption with YTPs. Left panel: After 20 h incubation with DMSO (-) or 250 nM YTP-75 (+). Right panel: After 4 h incubation with DMSO (0) or increasing doses of IAG933. The blots are representative of at least two repeats. **c**, Concentration-dependent antiproliferative effect of YTP-75 and TEAD lipid-binders VT104 and K-975. The number of viable NCI-H2052 cells following a 72 h incubation with the indicated compounds was quantified by resazurin assay. Values are mean of two independent experiments. Half-maximal growth inhibition is indicated by a dashed line. **d**, Real-time live cell measurements were performed over 6 days with the live-cell analysis system Incucyte in three indicated mesothelioma cell line models with a range of dose levels for IAG933 or the TEAD allosteric lipid pocket (LP) binders VT104 and K-975. All compounds displayed antiproliferative effects in long-term assays. MSTO-211H is a cell line with LATS1/2 deletion, NCI-H2052 and NCI-H226 are cell lines with NF2-inactivation. Mean of duplicates, or triplicates for DMSO, one-way ANOVA test were run for each cell line and each compound at concentration of 370 nM, as indicated in the table below the graphs. Source data

The cocrystal structure of IAG933 with TEAD3 (2 Å) shows a combination of polar and hydrophobic interactions with its Ω-loop pocket (Fig. 1a and Extended Data Fig. 1a–c), recognized as the interface of the PPI with YAP and TAZ^24^. IAG933 sits in a hydrophobic region created by the side chains of residues V266, I271, l296, F275, T395, F416 and V415 but also shares a salt bridge with E417, hydrogen bonds with K274, K298 and Q270 and a face-to-edge aromatic interaction with W300. This Ω-loop binding region is known to be conserved across the four human TEAD paralogs^26,27^, and YAP or TAZ binds to all four with similar affinities^28^. Consistent with this, surface plasmon resonance showed comparable binding of IAG933-related YTPs across all four paralogs (Extended Data Fig. 1d–f). Moreover, IAG933 and other YTP analogs disrupted the interaction between YAP and TEAD4 with nanomolar potency in a time-resolved fluorescence energy transfer (TR-FRET) assay (Extended Data Fig. 2a).

### Rapid TEAD inhibition by disruption of coactivator binding

**Extended Data Fig. 3:**
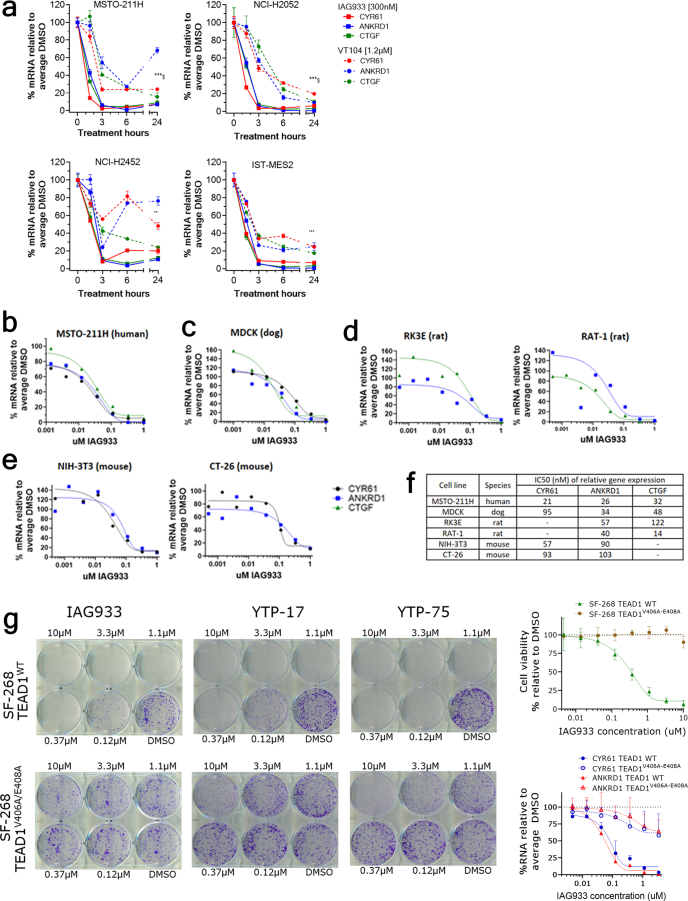
Cellular activity and selectivity. **a**, Time-dependent effect in quantitative measurements of TEAD target gene expression in cultured mesothelioma cell lines MSTO-211H, NCI-H2052, NCI-H2452, and IST-MES2, with IAG933 PPI inhibitor and VT104 TEAD allosteric inhibitor. Data shown for four replicates, Mean ± SD. Two-tailed paired t test VT-104 [1.2 µM]” vs. “IAG933 [300 nM] using the results from all 3 genes: **p = 0.0002, ***^$^, p = 0.0001***, p < 0.0001 for each cell line. **b-f**, Transcriptional modulation induced byIAG933 in cell lines from non-human species shows a similar range of activity after 24 h treatment. **b**, human, **c**, dog, **d**, rat, **e**, mouse cell lines were subjected to *in vitro* treatment with a concentration range of IAG933 for 16 h. mRNA levels of three YAP/TEAD target genes (*CYR61*, *ANKRD1* and *CTGF*), expressed as relative percentages vs the vehicle control (DMSO), are represented on the y-axis versus tested concentrations of IAG933 (μM) on the log-scaled x-axis. In some cell lines, one of the TEAD target genes could not be detected. **f**, Lists of the mean IC_50_ values (n = 2–4) of IAG933, calculated using Fit models (203) from XLFit (Microsoft). **g**, Right: characterization of the SF-268 cell clones derived from lentivirus infection with a TEAD1 WT or TEAD1^V406A/E408A^ mutant. The sensitivity to IAG933 is impaired in the mutant clone. Mean ± SD, results of 6 individual experiments. Left: Rescue experiments that demonstrate TEAD-selectivity of IAG933 and YTPs in a colony formation assay. Cell colonies obtained using CRISPR knock-in engineered clones of the SF-268 YAP-amplified glioma cell line assess the rescue of YTP effects. This cell line mainly expresses and depends on the TEAD1 isoform. Wild-type and TEAD1^V406A/E408A^ mutant clones were treated with indicated doses of IAG933, YTP-17, or YTP-75. The picture is representative of 3 individual experiments. Source data

Disruption of the YAP/TAZ–TEAD interaction by IAG933 and YTP-75 was demonstrated by coimmunoprecipitation (Fig. 1b and Extended Data Fig. 2b) following incubation of the Hippo-altered mesothelioma cell line MSTO-211H (*LATS1*/*LATS2* loss of function) with IAG933 or YTP-75, an IAG933 analog with a cellular potency within a similar range (Extended Data Fig. 2a). Furthermore, IAG933 treatment for 24 h almost completely inhibited the expression of direct TEAD target genes *CCN1*, *ANKRD1* and *CCN2* in both MSTO-211H cells and another Hippo-altered mesothelioma line NCI-H226 (*NF2* loss of function), with half-maximal inhibitory concentration (IC_50_) values between 11 and 26 nM (Fig. 1c). This transcriptional inhibition was rapid and maximized at 3 h after treatment (Fig. 1d and Extended Data Fig. 2c). Consistent with these pharmacodynamic (PD) data, IAG933 and IAG933 analogs displayed potent antiproliferative activity in Hippo-dependent cell lines (Fig. 1e), particularly in mesothelioma, which showed half-maximal growth inhibition (GI_50_) values between 13 and 91 nM irrespective of pathway alterations. Non-mesothelioma lines with Hippo alterations (SF-268, LMSU and NUGC-3) were more moderately sensitive (GI_50_ of ~1 μM). We compared the antiproliferative and PD activity of IAG933 with two allosteric TEAD inhibitors that bind to the TEAD LP, VT104 (ref. ^21^) and K-975 (ref. ^17^), in a panel of TEAD-dependent mesothelioma lines. IAG933 treatment resulted in a substantially more rapid and profound reduction in cell viability than the LP-binding compounds (Fig. 1f and Extended Data Fig. 2a,c), which required longer incubations to show substantive activity (Extended Data Fig. 2d). IAG933 also showed a faster and more complete inhibition of TEAD-dependent gene transcription (Extended Data Fig. 3a).

Consistent with the high conservation of the TEAD Ω-loop pocket across species^27^, IAG933 demonstrated comparable levels of PD activity against TEAD-dependent transcriptional targets in human, rodent and dog cells (Extended Data Fig. 3b–f), with IC_50_ values from 14 to 122 nM.

### Selective in vitro modulation of TEAD activity by IAG933

**Extended Data Fig. 4:**
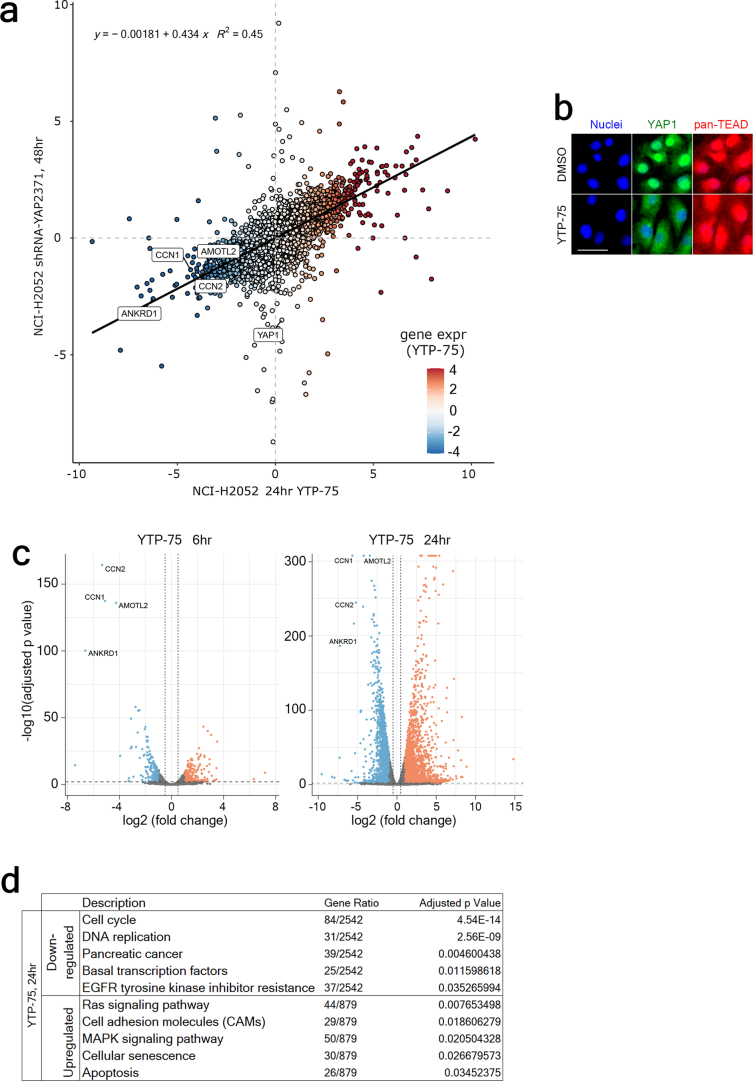
Compound effects on sub-cellular localization of YAP1 and transcriptional changes. **a**, Correlation analysis of RNA-seq results NCI-H2052 cell viability when treated with YTP-75 for 24 hr or subjected to YAP shRNA knockdown. R^2^ indicates the coefficient of determination of the linear model used for regression analysis. The results of 3 independent experiments are presented. **b**, Immunofluorescence microscopy shows nuclear/cytoplasmic localization of YAP1 and TEAD in the presence of 0.1% DMSO or 500 nM of YTP-75. NCI-H2052 cells were incubated for 4 h before immunofluorescence staining was performed. Nuclei were stained with Hoechst 33342. Size bar indicates 50 μm. The picture is representative of 3 individual experiments. **c**, Volcano plots representing gene expression changes based on RNA-Seq analyses of MSTO-211H cells treated with 250 nM of YTP-75 for 6 h or 24 h. p-values obtained by a two-sided Wald test were corrected for multiple testing using the Benjamini and Hochberg method. **d**, Summary table of the gene functional annotation analysis performed on genes differentially expressed based on the RNA-seq results. Two-sided hypergeometric test p values with Benjamini–Hochberg multiple-testing adjustment are shown. Source data

To evaluate selective target modulation by YTPs in cellular systems, we engineered a stable YTP-resistant TEAD1^V406A/E408A^ variant of the TEAD1-dependent, YAP-amplified glioma line SF-268, which maintains its interaction with endogenous YAP and TAZ. This variant was resistant to growth inhibition by IAG933, YTP-17 and YTP-75, whereas wild-type SF-268 cells remained sensitive (Fig. 1g and Extended Data Fig. 3g), confirming the exquisite on-target selectivity of IAG933 and IAG933 analogs. Previous PRISM-screening studies in a large cell panel showed that the cellular activity of allosteric TEAD inhibitors correlated with responsiveness to TEAD1 activity^29^. We therefore integrated genetic, transcriptomic^30^ and pharmacologic profiling to elucidate the functional selectivity of IAG933. Antiproliferative activity of IAG933 in a panel of 103 cancer cell lines showed significant overlap with the genetic sensitivity profile of averaged short hairpin RNA (shRNA) knockdown results from Project DRIVE^30^ for YAP, TAZ and TEAD1 (Fig. 1h). Consistent with the observed IAG933 activity in a subset of Hippo-unaltered cell lines (Fig. 1e), additional profiling on 263 cancer cell lines revealed higher sensitivity in cells that display high basal TEAD activity, as determined by a four-gene (*CCN1*, *CCN2*, *ANKRD1* and *AMOTL2*) transcriptional signature (Fig. 1i). Additionally, compound selectivity was further demonstrated by correlating RNA-sequencing (RNA-seq) gene expression changes from shRNA-mediated *YAP1* knockdown and YTP-75 treatment in the NCI-H2052 mesothelioma cell line (Extended Data Fig. 4a).

### YAP–TEAD direct inhibitors rapidly evict YAP from chromatin

**Fig. 2:**
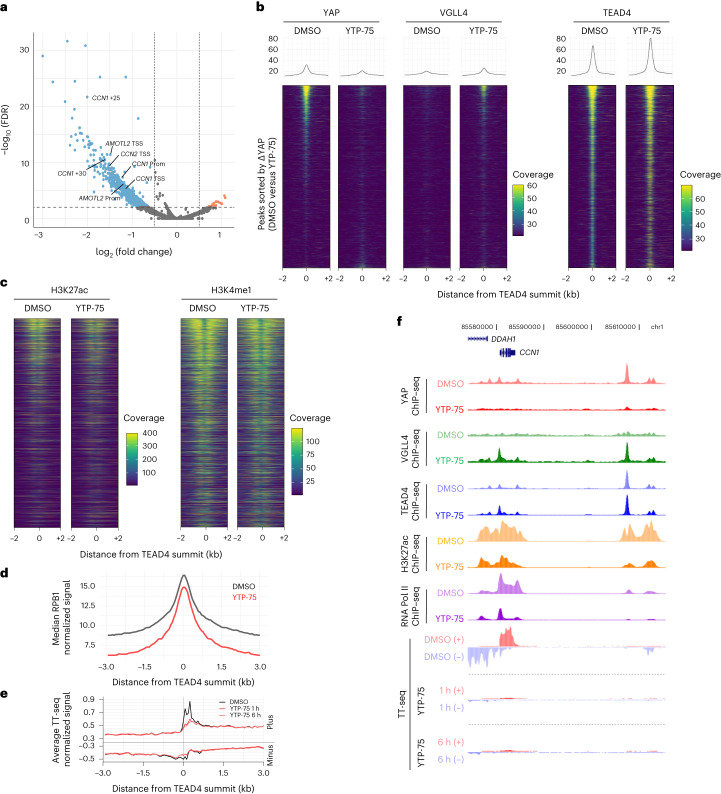
YTPs evict YAP from TEAD-occupied sites to reduce transcription of Hippo target genes. **a**–**d**, MSTO-211H mesothelioma cells treated for 24 h with 250 nM YTP-75 or DMSO. The results of three independent experiments are presented. **a**, Volcano plot of differential chromatin binding of YAP (YTP-75 versus DMSO). Representative peaks assigned to canonical Hippo target genes are highlighted; TSS, transcription start site; Prom, promoter; +*n*, distance to closest transcription start site (kilobases). **b**, ChIP–seq signal heat maps showing occupancy of YAP, VGLL4 and TEAD4 in TEAD4 peaks. Heat maps are sorted according to YAP differential occupancy (YTP-75 versus DMSO). **c**, Heat maps of H3K27ac and H3K4me1 ChIP–seq signals, with a similar representation as in **b**. **d**, Metaplot of RPB1 ChIP–seq distribution on TEAD4 sites in cells after treatment with DMSO or YTP-75. **e**, Metaplot of stranded TT-seq signal at TEAD4 sites in MSTO-211H cells 1 or 6 h after treatment with 250 nM YTP-75 or DMSO. **f**, Representative genome browser snapshot of the *CCN1* gene locus overlaying indicated ChIP–seq and TT-seq signals. Source data

Given the potency and selectivity of YTPs, we sought to characterize the epigenomic changes elicited by a 24-h treatment of YTP-75 in MSTO-211H mesothelioma cells. Chromatin immunoprecipitation with sequencing (ChIP–seq) analyses revealed a YTP-75-induced loss of YAP chromatin occupancy in a large number of regulatory elements (*n* = 1,058, log (fold change) > 0.5 or log (fold change) < –0.5 with a false discovery rate (FDR) of <0.01; Fig. 2a), including previously described enhancers and promoters of *CCN1* (*CYR61*), *CCN2* (*CTGF*) and *AMOTL2* (refs. ^31–33^). Furthermore, analysis of YAP binding at TEAD4 peaks showed that YAP signal intensity scaled with TEAD4 signal intensity (Fig. 2b). YTP-75 treatment resulted in near-complete loss of YAP signal at TEAD4 sites, with a concomitant gain in occupancy of the TEAD co-repressor VGLL4 (ref. ^34^; Fig. 2b), consistent with the competition model between VGLLs and YAP for binding to TEAD factors^35^. Further consistent with a YAP displacement from nuclear, chromatin-resident TEAD^36^, YTP-75 treatment induced YAP cytoplasmic relocation (Extended Data Fig. 4b). The downstream effects of YAP eviction were also explored. Notably, YTP-75 lowered the activity of regulatory elements at sites characterized by high YAP binding, as evidenced by a decrease in acetylated H3K27, while more moderately affecting the enhancer mark H3K4me1 (Fig. 2c). These impairments in enhancer activity translated into reduced occupancy of RNA polymerase II (RNA Pol II) subunit RPB1 (Fig. 2d). We then measured transcriptional outputs by using transient transcriptome sequencing (TT-seq) and RNA-seq methods and observed a rapid and direct impact of YTP-75 on the transcriptome. Remarkably, nascent RNA levels at TEAD4 binding sites were inhibited after only 1 h of treatment (Fig. 2e), both at target genes and distal regulatory elements (Fig. 2e). These data demonstrate a YTP-75-dependent effect on direct YAP–TEAD target genes by rapid shutdown of transcriptional elongation at the gene body as well as of RNA Pol II engagement and enhancer RNA expression at enhancer regions (Fig. 2f). Steady-state RNA-seq analysis after 6 and 24 h of treatment with YTP-75 demonstrated a consistent and progressive inhibition of direct target genes (Extended Data Fig. 4c) and genes downregulating cell cycle, DNA replication and general transcription factor pathways and upregulating MAPK and RAS signaling pathways as well as apoptosis (Extended Data Fig. 4d).

### IAG933 and YTP-75 achieve dose-dependent antitumor efficacy

**Fig. 3:**
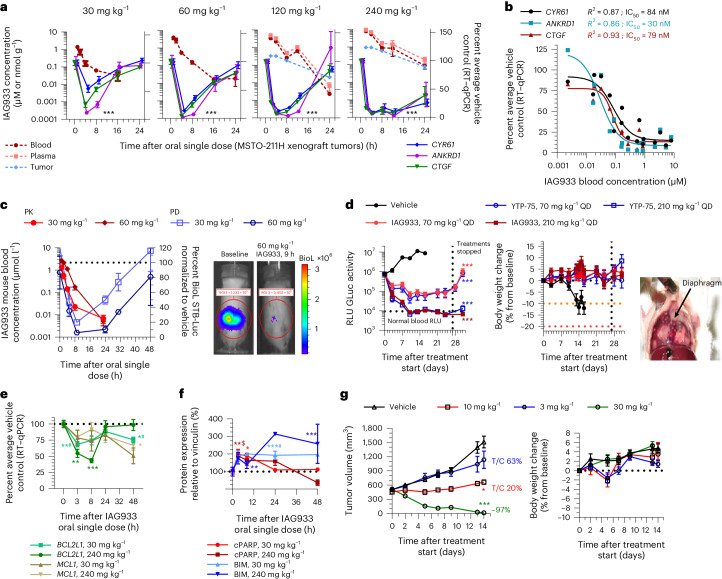
IAG933 demonstrates rapid PD and robust antitumor activity in mouse and rat MSTO-211H xenograft models. **a**, Inhibition of TEAD target gene expression in tumors after single-dose oral administration of IAG933. Data are shown as mean ± s.e.m.; *n* = 5 mice for vehicle and *n* = 3 mice for each dose per time point. Data were analyzed by one-way analysis of variance (ANOVA) using the results of all three target genes, treated versus vehicle, for all doses; ***P < 0.0001. Plasma, total blood and tumor exposures were quantified (mean of *n* = 3 mice); RT–qPCR, quantitative PCR with reverse transcription. **b**, Exposure-dependent inhibition of tumor target gene expression after single-dose oral administration of IAG933. In vivo IC_50_ values were calculated based on a 95% confidence interval for all genes, nonlinear fit to a simple logistic function. **c**, Kinetics of IAG933 bioluminescence inhibition in an in vivo reporter assay in mice bearing orthotopic pleural MSTO-211H tumors expressing firefly luciferase under the control of TEAD-responsive elements (MSTO-211H-STB-Luc cells). Data are shown as mean ± s.e.m.; *n* = 3 mice per group; BioL, bioluminescence; ROI, region of interest. **d**, Comparative dose-dependent effect on tumor volume and body weight of IAG933 and its close analog YTP-75 in orthotopic pleural MSTO-211H tumors. Body weight reduces when pleural tumor burden becomes excessive. Data are shown as mean ± s.e.m.; *n* = 3 mice per group. Data were analyzed by one-way ANOVA, treated versus vehicle, *P* < 0.0001 for treatment versus vehicle groups; RLU, relative light units; QD, once per day. **e**, Modulation of mRNA expression of antiapoptotic genes in tumors following administration of IAG933 in mice. Data are shown as mean ± s.e.m.; *n* = 3 mice per time point except *n* = 5 for the vehicle. Data were analyzed by one-way ANOVA with a Turkey’s multiple comparisons test (*P = 0.045, *^#^*P* = 0.0225, **^#^*P* = 0.006, **P = 0.0087 and ***P = 0.0002). **f**, Western blot analysis of proapoptotic protein expression in tumors after IAG933 dosing in mice. Data are shown as mean ± s.e.m.; *n* = 3 mice per time point. Quantifications were performed using images and a one-way ANOVA with a Turkey’s multiple comparisons test (*P = 0.0165, **P = 0.005, **^$^*P* = 0.041, ***^#^*P* < 0.001 and ***P = 0.0006). **g**, Antitumor efficacy and tolerability of IAG933 in a rat MSTO-211H xenograft model. Data are shown as mean ± s.e.m.; *n* = 5 rats per group. Data were analyzed by one-way ANOVA with a Tukey’s multiple comparison test (*P = 0.029 at 10 mg kg^−1^ and *P* < 0.0001 at 30 mg kg^−1^ versus vehicle groups). T/C, tumor control ratio. Source data

**Extended Data Fig. 5:**
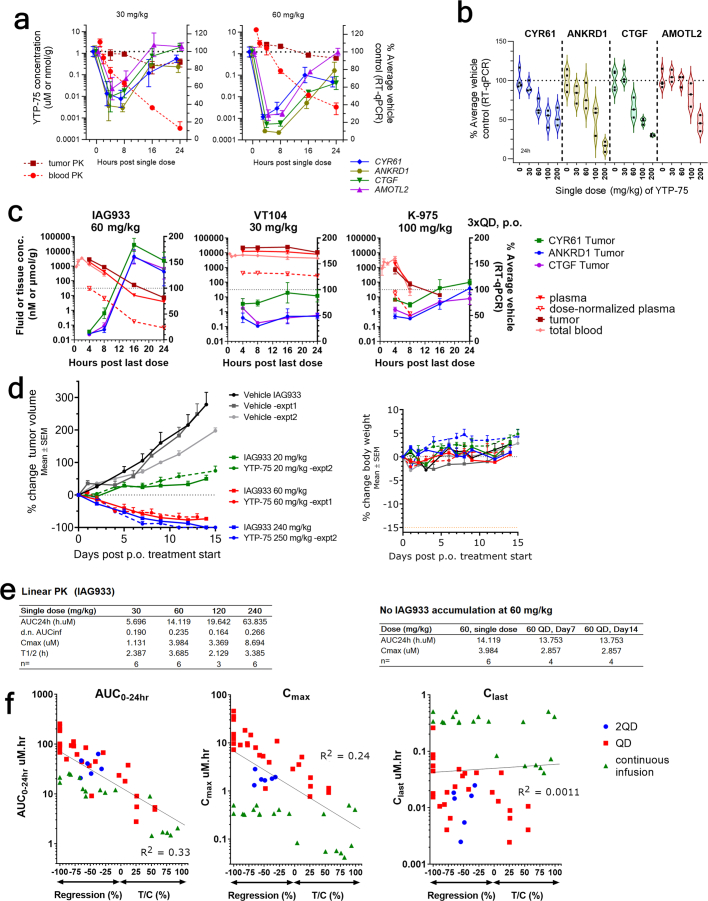
PK/PD/efficacy relationships of TEAD inhibitors in mesothelioma mouse subcutaneous xenografts. **a**, Kinetics of inhibition of TEAD target genes expression in MSTO-211H subcutaneous tumors after single administration of YTP-75 at indicated doses. Mean ± SEM, n = 4 mice for vehicle group, n = 3 mice for each treatment point. One-way ANOVA test using the results of all three target genes, treated vs untreated, p < 0.0001***. Total blood and tumor exposures of YTP-75 were measured in three animals. **b**, TEAD target gene expression- in MSTO-211H tumors 24 h after single-dose administration of YTP-75 at four dose levels. n = 4 mice for vehicle, n = 3 mice for each treatment point. **c**, TEAD-related pharmacodynamics of IAG933 and TEAD allosteric lipid pocket (LP) binders VT104 and K-975 in NCI-H226 mesothelioma murine xenografts. The inhibition kinetics of TEAD target gene expression was assessed by RT-qPCR after administration of three daily doses of compounds. Plasma, total blood and tumor exposures are displayed. Mean ± SEM, n = 3 mice per point. **d**, Comparative dose-dependent antitumor effect of IAG933 and its close analog YTP-75 in subcutaneous MSTO-211H xenografts. Three experiments are shown on the graph. Percentage change of tumor volume and body weight from first day of treatment are shown. Mean ± SEM, n = 5 mice per group, except for IAG933 240 mg/kg for which only two animals were used. **e**, PK parameters in mice for IAG933 showing linear PK and no compound accumulation at 60 mg/kg at the indicated number of days. (d.n. dose-normalized). Mean values are shown. **f**, Correlation (Pearson r) between pharmacokinetic (PK) parameters and antitumor responses following two weeks of YTP-75 treatment. The data were collected from five distinct pharmacology studies conducted in Nude mice with MSTO-211H subcutaneous xenograft tumors. The mice were treated either daily (QD), twice daily (2QD), or via continuous infusion using minipump delivery. The utilization of a twice daily dosing schedule or continuous infusion enabled the assessment of the correlation between antitumor efficacy and PK parameters. Source data

IAG933 was assessed in mouse MSTO-211H cell-derived xenograft (CDX) models at single doses between 30 and 240 mg per kg of body weight (mg kg^−^^1^) administered by oral gavage. Dose-related blood exposure was observed with a time at maximal concentration (*T*_max_) of ~1–2 h, correlating with a dose/exposure-dependent TEAD target gene inhibition commencing at ~2 h after dosing (Fig. 3a,b). The in vivo blood IC_50_ for target gene inhibition of 64 nM was slightly higher than the in vitro IC_50_ of 11–26 nM for MSTO-211H cells (Fig. 1c). An in vivo reporter assay, using luciferase expression under TEAD-responsive elements in orthotopic pleural MSTO-211H tumors, showed rapid and profound loss of bioluminescence following a single dose of IAG933 (Fig. 3c), followed within a few hours by a rebound to baseline due to the relatively short half-life of IAG933 in mice. Similar PK/PD findings were observed for the IAG933 analog YTP-75 (Extended Data Fig. 5a,b), demonstrating deep and quick TEAD in vivo transcriptional inhibition by both compounds.

Comparative studies of IAG933 versus VT104 and K-975 after three daily oral doses were undertaken in the NCI-H226 CDX mesothelioma model that is known to be sensitive to both allosteric inhibitors^17,21^. The three compounds displayed different properties, with IAG933 appearing more potent, reducing TEAD target RNA expression to 2–21% of baseline despite a *C*_max_ 2.6-fold lower than VT104 (Extended Data Fig. 5c). The longer half-life of VT104 resulted in stable 24-h blood and tumor exposure, consistent with a more moderate but sustained PD response.

Under extended daily dosing (2–4 weeks) of mouse models bearing orthotopic or subcutaneous MSTO-211H xenografts, IAG933 or YTP-75 antitumor effects were comparable and accompanied by significant dose-dependent responses that ranged from near stasis to profound tumor regression. IAG933 prevented mouse morbidity induced by increasing tumor burden in pleura, and antitumor responses were sustained over 4 weeks of treatment (Fig. 3d and Extended Data Fig. 5d). Encouragingly, despite IAG933 having cellular activity in mouse cells (Extended Data Fig. 3e,f), no weight loss or tolerability issues were observed in these mouse experiments. Steady-state IAG933 exposure was reached rapidly and was linear and dose proportional, with no accumulation at 60 mg kg^−1^; Extended Data Fig. 5e). Pharmacological assessment of YTP-75, including twice daily administration and continuous infusion via micropump, established that the antitumor response correlated best with 24-h area under the concentration–time curve values (Extended Data Fig. 5f).

**Extended Data Fig. 6:**
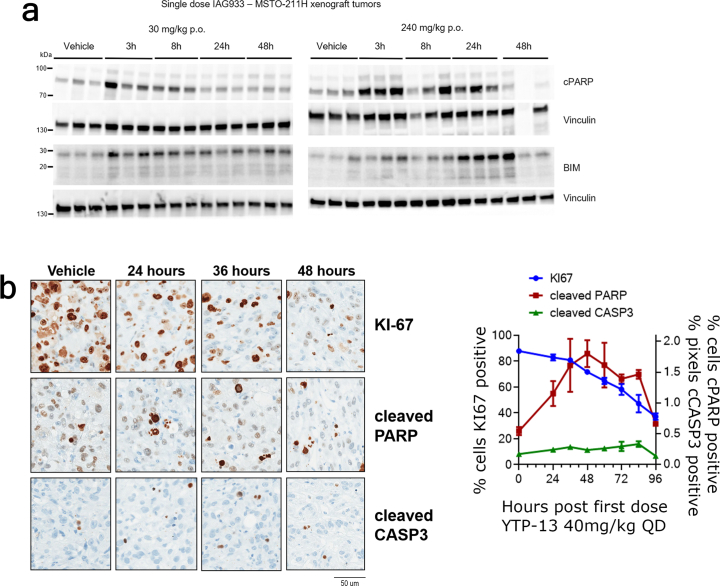
*In vivo* evaluation of pro-apoptotic protein expression, cell apoptosis and proliferation in mesothelioma tumors. **a**, Evaluation of changes in protein expression in MSTO-211H xenograft tumors after administration of a single dose of IAG933 at 30 and 240 mg/kg. Western-blots detected cleaved PARP and BIM proteins, Vinculin was used as loading control. This blot is representative of 2 individual experiments. **b**, Immunohistochemistry changes following treatment of NCI-H2052 xenograft tumors with three daily doses of YTP-13. Representative panels are shown for Ki-67, cleaved PARP and cleaved CASP3. Mean and SEM are shown, n = 3 per point. Source data

To gain insight into the effects of TEAD inhibition by YTPs, we further examined their impact on cell proliferation and apoptosis in xenograft models. A single dose of IAG933 induced proapoptotic signals, as evidenced by the presence of cleaved PARP and BIM proteins, while simultaneously decreasing the expression of the antiapoptotic genes *BCL2L1* and *MCL1* in MSTO-211H tumors (Fig. 3e,f and Extended Data Fig. 6a). Additionally, immunohistochemistry analysis of NCI-H2052 xenograft tumors treated with the IAG933 analog YTP-13 for 3 days demonstrated an increase in cleaved PARP levels along with a reduction in the expression of the cell proliferation marker Ki67 (Extended Data Fig. 6b). These results confirm that YTPs exhibit both cytostatic and cell killing effects in implanted human mesothelioma tumors, further supporting their potential as therapeutic agents.

### IAG933 erradicates tumors in a rat model at tolerated doses

**Extended Data Fig. 7:**
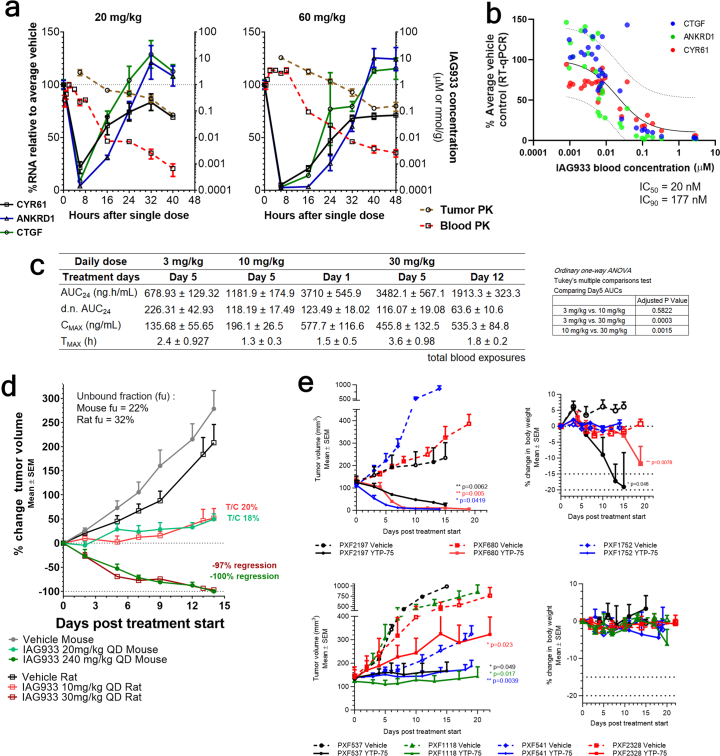
Exposure-response relationships of IAG933 in a MSTO-211H mesothelioma rat xenograft model, and YTP-75 antitumor efficacy in mouse mesothelioma PDX models. **a-d**, The MSTO-211H subcutaneous xenograft model was developed in irradiated Nude rats. Following cell implantation, measurable tumors developed in all animals. **a**, Exposure-dependent inhibition of the expression of three TEAD target genes in MSTO-211H tumors after single-dose administration of IAG933 in rats. Mean ± SEM; n = 3 rats per point. **b**, Correlation of IAG933 blood concentration with TEAD target gene expression in MSTO-211H tumors after single-dose IAG933 administration. *In vivo* IC_50_ and IC_90_ were calculated based on a 95% confidence interval for all genes (fit to a simple logistic function). **c**, PK parameters in rats for IAG933 showing linear PK and no compound accumulation at 30 mg/kg at the indicated number of days. One-way ANOVA test results are shown for the comparison of Day 5 AUC_24h_ values. Mean ± SEM; n = 5 rats per group, except for the Day1 30 mg/kg group: n = 3. **d**, Comparative representation of antitumor efficacy in MSTO-211H xenograft models of mice and rats. Daily administration of IAG933 at specified doses was employed. By overlaying the anti-tumor response curves from mice and rats in 2-week experiments, corresponding doses for tumor near-stasis (10 mg/kg QD for rats vs. 20 mg/kg QD for mice) and near-complete regression (30 mg/kg QD for rats vs. 240 mg/kg QD for mice) could be determined. Mean ± SEM; n = 5 animals per group, except for the IAG933 240 mg/kg, n = 2 mice. **e**, YTP-75 was administered at 240 mg/kg daily in PDX models from Charles River. Only PDX models sensitive to treatment are shown here; two which did not respond are not represented. All treatments were well tolerated. Mean ± SEM; n = 4, 5, or 6 mice per group depending on the PDX models. Two-tailed paired t test results are shown for the comparison of vehicle vs. YTP-75-treated for each PDX model, significant p values are indicated in the graphs. Source data

Extending our in vivo studies beyond mice, we also evaluated IAG933 in a subcutaneous MSTO-211H rat xenograft model. Target gene inhibition kinetics after single-dose IAG933 were similar to those observed in the mouse model (Extended Data Fig. 7a,b), whereas the averaged *CCN2*/*ANKRD1*/*CCN1* IC_50_ of 20 nM was approximately threefold lower and similar to the in vitro IC_50_ values. After 2 weeks of daily dosing, tumor stasis was observed at 10 mg kg^−1^, and complete regression was seen at 30 mg kg^−1^ in four of five animals (Fig. 3g). Exposures were dose proportional, and no compound accumulation was detected over 12 days of daily treatment (Extended Data Fig. 7c). No body weight loss was observed, and treatments were well tolerated. Comparing rat and mouse model response curves established a dosing equivalence between 30 mg kg^−1^ once a day in rats and 240 mg kg^−1^ once a day in mice (Extended Data Fig. 7d).

### IAG933 activity in mesothelioma and Hippo-altered xenografts

**Fig. 4:**
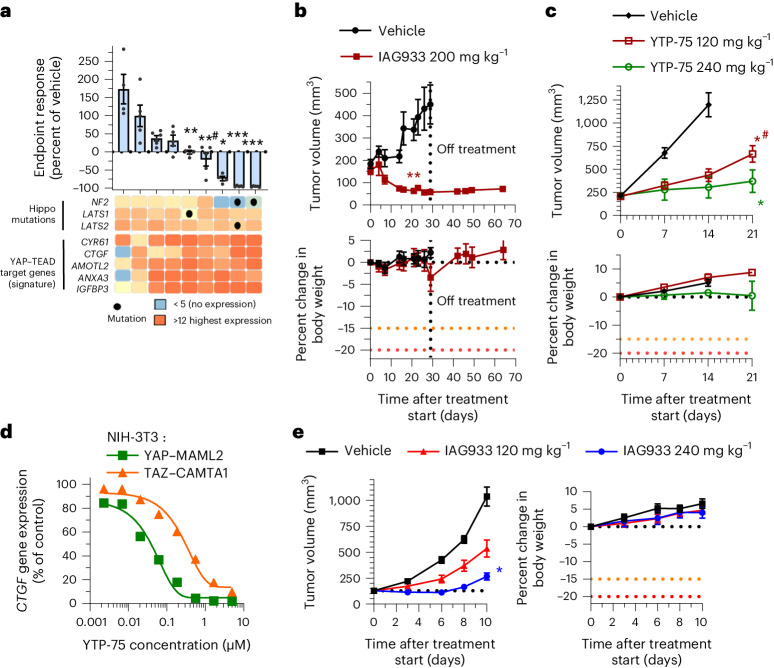
Antitumor efficacy in mesothelioma PDX and Hippo-altered non-mesothelioma models. **a**, Endpoint tumor responses of nine mesothelioma PDX models treated with 240 mg kg^−1^ YTP-75 once daily for 14–21 days. Data are shown as mean ± s.e.m.; *n* = 4, 5 or 6 mice per group depending on the model. Data were analyzed by two-tailed unpaired *t*-test (*P = 0.014, **P = 0.002, **^#^*P* = 0.0001 and ***P < 0.0001). Gene expression levels and genetic alterations across models retrieved from the Charles River database are displayed on the bottom. **b**,**c**, Antitumor efficacy of IAG933 or YTP-75 and change in body weight in two NF2 loss-of-function mouse xenograft models of non-mesothelioma cancers. Data are shown as mean ± s.e.m. and were analyzed by two-tailed paired *t*-tests (**P = 0.001, *P = 0.0146 and *^#^*P* = 0.0397); *n* = 5 per group (5938-HX triple-negative breast ductal carcinoma PDX model; **b**) and *n* = 6 per group (NCI-H292 lung carcinoma CDX model; **c**). **d**, Dose-dependent inhibition of the *CCN2* TEAD target gene by YTP-75 (24-h treatment) in NIH-3T3 cells stably expressing *YAP*–*MAML2* or *TAZ*–*CAMTA1* fusion genes. Calculated IC_50_ values are between 82 and 292 nM. **e**, Dose-dependent antitumor efficacy and change in body weight of IAG933 in subcutaneous NIH-3T3 xenograft tumors expressing TAZ–CAMTA1. Data are shown as mean ± s.e.m. and were analyzed by one-way ANOVA; *n* = 6 per group; *P = 0.0252. Source data

Mesothelioma pathogenesis frequently involves genetic alterations in tumor suppressor genes of the Hippo signaling cascade, including *NF2* and *LATS1*/*LATS2*, in an estimated 32–50% of cases^9,37–39^. We explored the antitumor efficacy of YTPs in differing mesothelioma genetic backgrounds in a panel of nine human-derived xenograft (PDX) mouse models treated daily with YTP-75. Significant tumor responses were observed in seven of nine models, with deep tumor regressions in three *NF2*-altered models and durable tumor stasis in four other models without reported Hippo alterations (Fig. 4a and Extended Data Fig. 7e). Interestingly, the two tumor models that did not respond displayed the lowest basal expression of TEAD target genes (Fig. 4a). *NF2* mutations have also been detected at low prevalence (~1–2%) in other solid tumors^9,38,40^. To explore YTP activity in such cases, we assessed IAG933 in an *NF2*-altered PDX model of triple-negative breast cancer (5938-HX) and YTP-75 in a CDX model of *NF2*-altered lung carcinoma (NCI-H292). Both models showed an antitumor response to treatment, but while 5938-HX underwent tumor regression (Fig. 4b), the NCI-H292 model showed a lesser inhibition of tumor growth (Fig. 4c).

**Extended Data Fig. 8:**
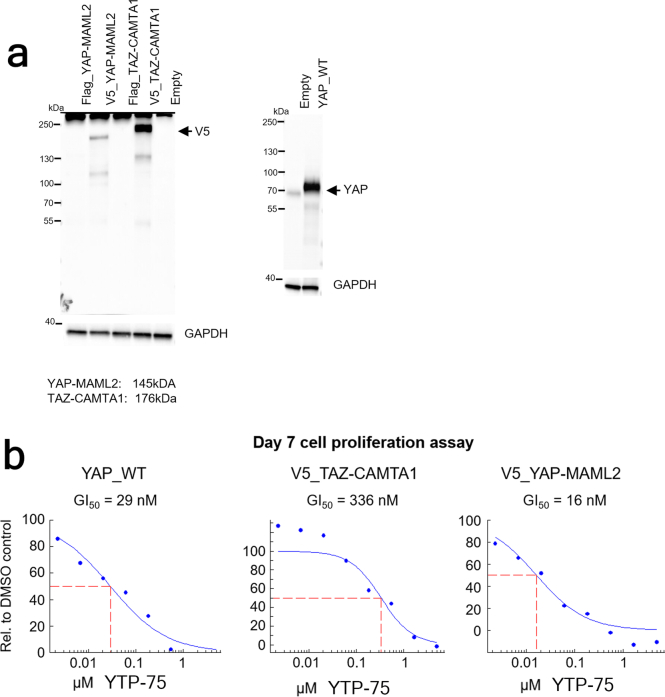
NIH-3T3 engineered cell line models transformed with YAP1 or TAZ fusion genes show sensitivity to IAG933 or YTP-75. The NIH-3T3 cell line was transformed with the YAP-MAML2, TAZ-CAMTA1, or YAP1 genes. Lentivirus infection allowed for stable integration of the construct. **a**, Western-blot analysis with V5, YAP1 or GAPDH antibodies, showing expression of the exogenous proteins. This blot is representative of 3 individual experiments. **b**, Cellular activity of YTP-75 represented by effects on cell viability after a 7-day treatment in NIH-3T3 cells expressing indicated genes. GI_50_’s were calculated from two independent experiments. Source data

Other Hippo alterations include fusion oncoprotein drivers TAZ–CAMTA1 and YAP–MAML2 that confer TEAD dependency in soft tissue sarcomas, such as epithelioid hemangioendothelioma^41^ and porocarcinoma^42^. Notably, both cell cultures and implanted tumors of NIH-3T3 cells transformed by stable overexpression of these fusions^41^ were also sensitive to IAG933 and YTP-75 (Fig. 4d,e and Extended Data Fig. 8).

### IAG933 combination treatment improves RTK inhibitor efficacy

Co-inhibition of EGFR and TEAD by osimertinib and VT104, respectively, has previously been shown to enhance osimertinib tumor response in NSCLC models^43^. Elevated YAP activity has been described in HER2-positive cancers and relapsing cancers^3,8^, and YAP–TEAD activation has been linked to trastuzumab resistance^8,44^. Moreover, recent data suggest that TEAD activation maintains a minimal residual disease under RTK inhibitor treatment^6,43^. Therefore, co-inhibition of TEAD could be essential for eradicating RTK-mediated cancers and achieving tumor elimination.

**Fig. 5:**
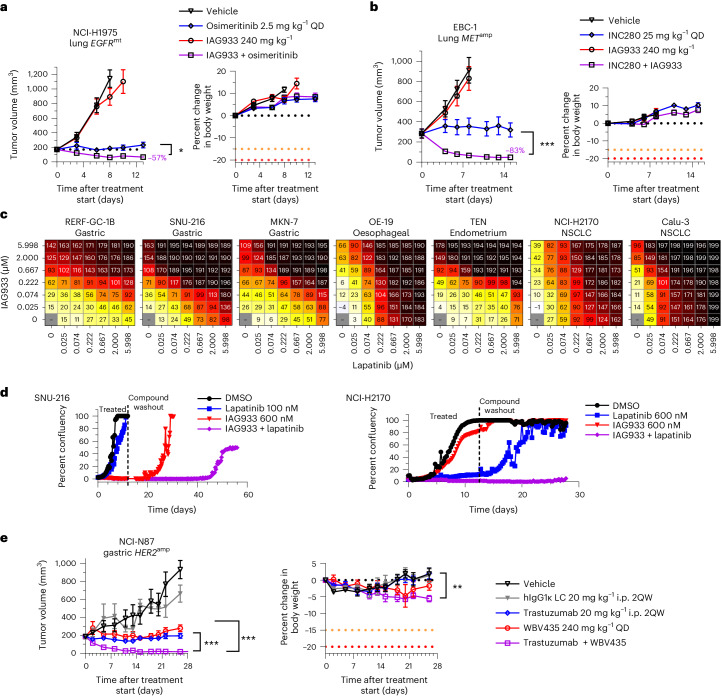
IAG933 enhances responses to EGFR, MET and HER2 RTK inhibitors. **a**,**b**, Tumor responses and body weight changes in mouse CDX lung cancer xenograft models. Data are shown as mean ± s.e.m. and were analyzed by one-way ANOVA; *n* = 6 per group (*P = 0.252 and ***P < 0.001). **a**, NCI-H1975 *EGFR*-mutant (*EGFR*^mt^) xenografts treated with osimertinib, IAG933 or both. **b**, EBC-1 *MET*-amplified (*MET*^amp^) xenografts treated with capmatinib, IAG933 or both. **c**,**d**, IAG933 enhances HER2 inhibitor efficacy in *HER2*-amplified (*HER2*^amp^) tumor cell lines. **c**, Short-term (6-day) treatment matrices show IAG933 dose-dependent enhancement of lapatinib antiproliferative activity. Growth inhibition (%) is shown in relation to treatment start: 0–99%, delayed proliferation; 100%, growth arrest/stasis; 101–200%, reduction in cell number/cell death. Data are shown as the mean values of triplicates. **d**, Long-term lapatinib and IAG933 combination treatment in SNU-216 gastric cancer and NCI-H2170 NSCLC cells. Data are derived from live-cell imaging experiments and are presented as the mean values of duplicates. **e**, Antitumor responses and body weight changes of the mouse NCI-N87 *HER2*-amplified gastric cancer CDX model to vehicle (*n* = 4), IgG1 control (*n* = 4), trastuzumab (*n* = 7), YTP-75 (*n* = 7) or trastuzumab + YTP-75 (*n* = 7). Data are shown as mean ± s.e.m. and were analyzed by one-way ANOVA (**P = 0.076 and ***P < 0.0001); LC, light chain; i.p., intraperitoneal; 2QW, twice per week; hIgG1κ, human IgG1κ. Source data

Consistent with this concept, IAG933 plus osimertinib showed enhanced antitumor benefit, leading to rapid regression in the *EGFR*-mutated NCI-H1975 CDX model of NSCLC (Fig. 5a). Furthermore, IAG933 plus the MET inhibitor capmatinib induced profound tumor shrinkage in the EBC-1 *MET*-amplified CDX model of lung cancer, while no activity was seen for IAG933 alone (Fig. 5b). Despite modest single-agent effects by IAG933 in a panel of seven *HER2*-amplified cell lines from various cancer indications, dose-dependent combination activity was seen for IAG933 with the HER2 inhibitor lapatinib (Fig. 5c), and prolonged combination activity after the end of treatment was observed in lengthier in vitro studies (Fig. 5d). In vivo, the *HER2*-amplified NCI-N87 gastric carcinoma xenograft model underwent complete tumor regression with the combination of YTP-75 and trastuzumab (Fig. 5e). Hence, a combination benefit is observed with YTPs in cancer models that are driven by different RTKs, indicating a shared underlying mechanism and presenting an opportunity for combining these therapeutic agents.

### IAG933 improves response to JDQ443 by inducing apoptosis

**Fig. 6:**
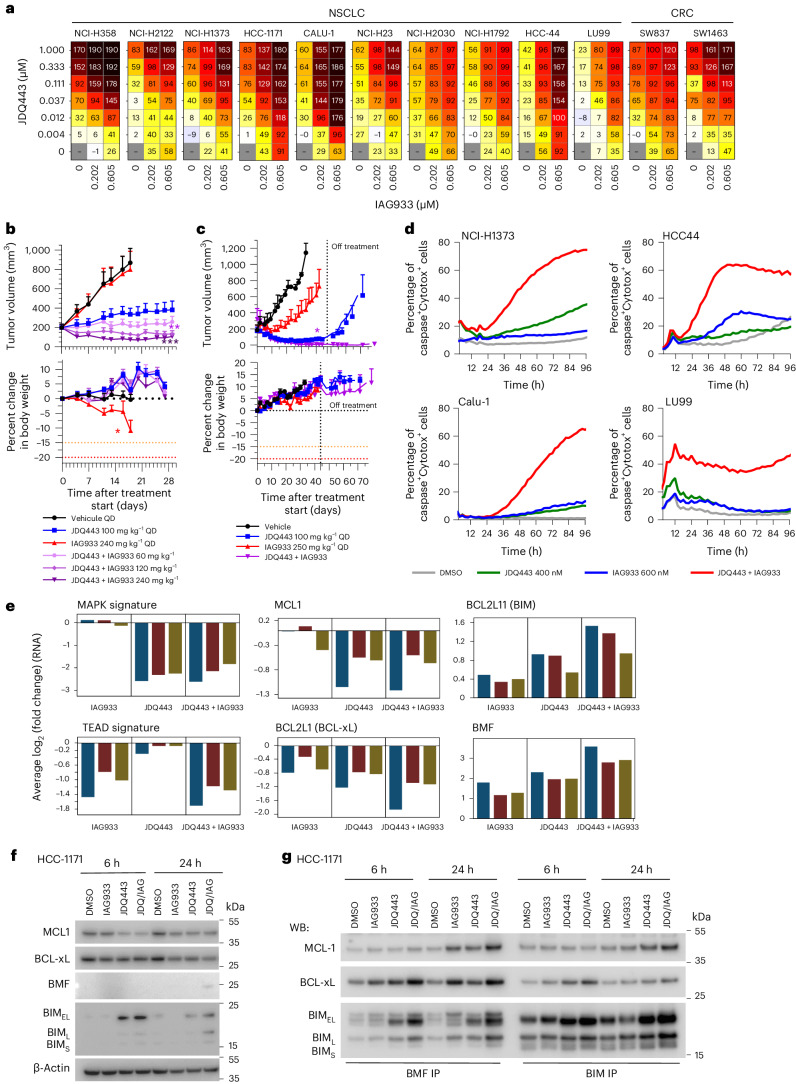
Synergistic antitumor efficacy of JDQ443 + IAG933 in *KRAS*^G12C^-mutated cancer models involves convergence of apoptotic regulators to induce cell death. **a**, Cell viability dosing matrices (7 days) show a combination benefit for IAG933 and JDQ443 in a range of *KRAS*^G12C^-mutated NSCLC and CRC cell lines. Data represent the mean values of triplicates. **b**,**c**, In vivo antitumor efficacy and tolerability of JDQ443 + IAG933 in the NCI-H2122 CDX (**b**; *n* = 6 per group) and 2094-HX PDX (**c**; *n* = 3 per group) NSCLC models (*KRAS*^G12C^). Treatment was discontinued at day 45 for 2094-HX to assess tumor eradication. Data in **b** are shown as mean ± s.e.m. and were analyzed by one-way ANOVA comparing JDQ443 and combinations. Data in **c** are shown as mean ± s.e.m. and were analyzed by two-tailed paired *t*-test comparing JDQ443 and combinations. **d**, Kinetics of apoptotic cell death induction by IAG933 and JDQ443 in four cell lines by live-cell imaging assessing caspase activity and cell death. Data represent the mean values of triplicates. **e**, RNA-seq following treatment of NSCLC cell lines Calu1, HCC1171, NCI-H1373, NCI-H23 and HCC44 with JDQ443 (400 nM), IAG933 (600 nM) or both. The average log_2_ (fold change) versus vehicle control across all five lines is reported for the indicated expression signatures or individual gene. **f**,**g**, Apoptosis factors in HCC1171 cells treated with JDQ443 (400 nM), IAG933 (600 nM) or both. Total cell lysates were subjected to immunoblotting analysis in one replicate with the indicated antibodies (**f**) or were used for immunoprecipitation with either BMF or BIM before immunoblotting (**g**); JDQ/IAG, JDQ443 + IAG933; WB, western blot; BIM-EL, BIM extra long; BIM-L, BIM long; BIM-S, BIM short. Source data

**Extended Data Fig. 9:**
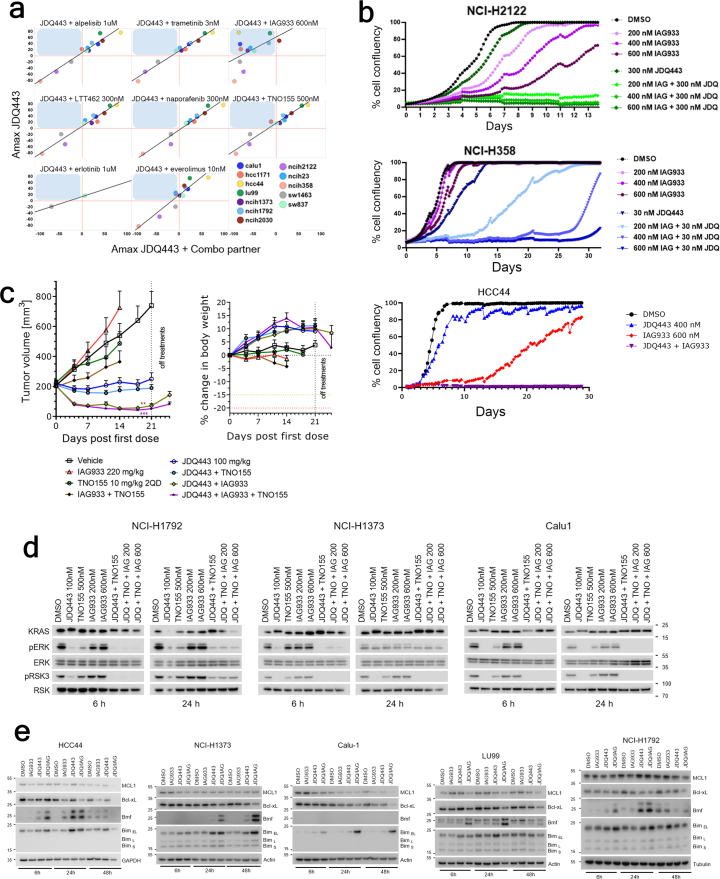
Effects of JDQ443 + IAG933 combination treatment on *KRAS*^*G12C*^-mutated NSCLC models. **a**, The maximal antiproliferative effect (Amax) was determined for JDQ443 with (x-axis) and without (y-axis) the indicated combination partner using a 7-day assay (CellTiter-Glo). Cell lines falling in the blue-shaded quadrant were those where a given combination was more inhibitory than JDQ443 alone. For each cell line indicated, the Amax was derived from a titration of JDQ443 (from 1.6 µM) with a fixed dose of other inhibitors. Results of 12 different cell lines are shown. **b**, Live-cell confluency was measured over 30 days using an Incucyte device with 3 cell lines treated as indicated. Mean of duplicates for HCC44, and 5 replicates for NCI-H2122 and NCI-H358. **c**, *In vivo* anti-tumor efficacy and body weight monitoring in the NCI-H2122 *KRAS*^*G12C*^-mutated NSCLC CDX model. Treatments were given until day 21. Mean ± SEM; n = 7 per group, One-way ANOVA, Tukey’s multiple comparisons test, show significant differences for JDQ443 vs. JDQ443/IAG933 **p = 0.0007, and JDQ443/TNO155 vs. JDQ443/TNO155/IAG933, ***p < 0.0001. **d**, Cultured NCI-H1792, NCI-H1373 and Calu1 *KRAS*^*G12C*^-mutated NSCLC cells were treated as indicated for 6 h and 24 h. Total cell lysates were prepared and subjected to immunoblot analysis with the indicated antibodies. Some blots were partially shown in a previous report^48^. Each experiment was performed once and repeated in several cell lines. **e**, Cultured *KRAS*^*G12C*^-mutated NSCLC cell lines were treated with JDQ443 400 nM and IAG933 600 nM alone or in combination for 6 h and 24 h. Total cell lysates were prepared and subjected to immunoblot analysis with the indicated antibodies. Each experiment was performed once and repeated in several cell lines. Source data

Despite the impact of selective KRAS^G12C^ inhibitors on mutant cancers, their clinical effectiveness is generally less pronounced than RTK inhibitors. Overcoming resistance to KRAS^G12C^ inhibitors remains a challenge, prompting ongoing clinical trials that investigate combination therapies^12^. In line with the findings obtained from allosteric TEAD inhibitors^45–47^, IAG933 and the Novartis KRAS^G12C^ inhibitor JDQ443 (ref. ^48^) displayed strong combination benefit in a panel of *KRAS*^G12C^-mutated NSCLC and CRC cell lines (Fig. 6a). IAG933 compared favorably to other JDQ443 candidate partners, such as inhibitors of SHP2, MEK, ERK or PIKα, by causing a notable shift in maximal growth inhibition across cell lines (Extended Data Fig. 9a). In long-term proliferation assays, we observed robust and sustained inhibition of cell growth when combining subefficacious concentrations of JDQ443 and IAG933, which modestly delayed cell proliferation as single agents (Extended Data Fig. 9b). Consistently, in vivo, upfront addition of IAG933 deepened responses to JDQ443 in NCI-H2122 NSCLC xenografts (Fig. 6b), with this combination outperforming JDQ443 plus the SHP2 inhibitor TNO155 (Extended Data Fig. 9c). This antitumor combination effect was also observed in a PDX model of NSCLC, with no tumor regrowth observed for 30 days after end of treatment (Fig. 6c).

Live-cell imaging using caspase-3/caspase-7 and cell death reporters in four *KRAS*^G12C^-mutated NSCLC cell lines revealed that the JDQ443 + IAG933 combination led to apoptotic signals at concentrations where single agents showed minimal activity (Fig. 6d). To gain mechanistic insight, a transcriptomics analysis was performed on a panel of five *KRAS*^G12C^-mutated NSCLC cell lines treated with JDQ443 and/or IAG933. Gene expression signatures for YAP–TEAD^9^ and MAPK^49^ were downmodulated mainly by IAG933 and JDQ443, respectively, and depth of inhibition was not further suppressed in combination, suggesting minimal cross-pathway inhibition (Fig. 6e). IAG933 did not deepen or lead to a more sustained MAPK pathway inhibition when combined with JDQ443, as shown by western blotting of phospho-ERK and phospho-RSK3 (Extended Data Fig. 9d). Given the observed induction of apoptosis specifically in the combination setting, we examined the expression levels of proapoptotic (*BCL2L11* (*BIM*) and *BMF*) and antiapoptotic (*MCL1* and *BCL2L1* (*BCLxL*)) regulators, revealing that their modulation was often more pronounced in combination^6^ (Fig. 6e). Protein levels reflected these mRNA changes, although with more variability across cell lines and time points (Fig. 6f and Extended Data Fig. 9e). Immunoprecipitation experiments further revealed that remaining levels of antiapoptotic proteins MCL1 and BCLxL were efficiently sequestered by proapoptotic BIM and BMF in the combination setting (Fig. 6g). This suggests that the upfront benefit of IAG933 and KRAS^G12C^ inhibitor cotreatment results from a convergence onto pro- and antiapoptotic factors, leading to apoptotic cell death.

### TEAD inhibition boosts the KRAS^G12D^ blockade effect in tumors

**Extended Data Fig. 10:**
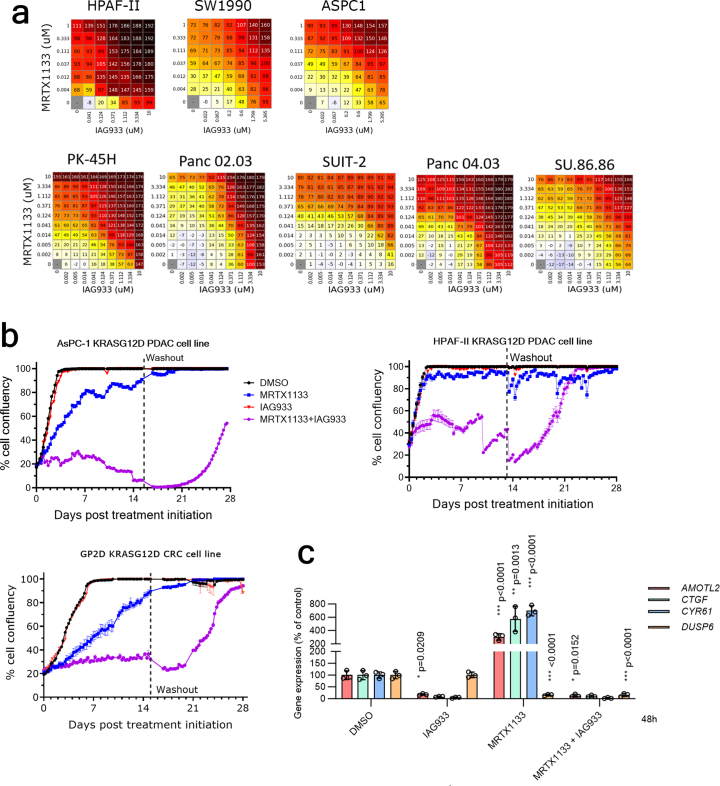
IAG933 improves the antiproliferative activity of the *KRAS*^*G12D*^ mutant-specific inhibitor MRTX1133 in KRAS^G12D^-dependent pancreatic and colorectal cancer cells, and prevents MRTX1133-induced TEAD activation. **a**, Antiproliferation dose matrices of MRTX1133 and IAG933 combinations in 8 different PDAC cell lines in 6-day assays. Mean of three experiments is shown. **b**, Combinations of IAG933 (600 nM), and MRTX1133 (40 nM) in extended cancer cell culture assays (Incucyte). Mean of n = 2 (AsPC-1, GP2D) or n = 3 independent experiments (HPAF-II), Mean ± SEM. **c**, MAPK pathway (*DUSP6*) and TEAD reporter gene expression in HPAF-II PDAC cells treated with IAG933, MRTX1133, or both, for 48 h (mean n = 3 biological replicates) shows IAG933 abrogation of MRTX1133-induced TEAD activity. Mean ± SD. One-way ANOVA test results are shown for the comparison vs. DMSO control values, p values are indicated on the graph. Source data

G12D is the most common KRAS mutation and is particularly prominent in PDAC and CRC^50^. TEAD2 paralog activation by YAP has been shown to compensate for loss of KRAS^G12D^ activity in PDAC tumor models^5^, suggesting a benefit to co-inhibiting KRAS^G12D^ and TEAD2. A combination benefit for IAG933 with the cell-active KRAS^G12D^ inhibitor MRTX1133 (ref. ^51^) was observed in a panel of eight *KRAS*^G12D^-mutated PDAC cell lines (Extended Data Fig. 10a). Long-term application of this combination strongly reduced cell confluency with a sustained post-treatment effect in two PDAC cell lines and a CRC line (Extended Data Fig. 10b). Notably, HPAF-II cells exhibited elevated expression of TEAD target genes following 48 h of treatment with MRTX1133, an effect that was counteracted by simultaneous inhibition of TEAD using IAG933. Expression of the *DUSP6* gene, indicative of MAPK signaling activation, was suppressed by MRTX1133 single treatment or in combination (Extended Data Fig. 10c).

Overall, pharmacological inhibition of TEADs by IAG933 not only enhances antitumor responses for KRAS^G12C^-mutant-specific inhibitors but also for KRAS^G12D^, indicating its broad potential as a combination partner in targeting KRAS mutations.

### IAG933 combination shows benefit in *BRAF*^V600E^-altered tumors

**Fig. 7:**
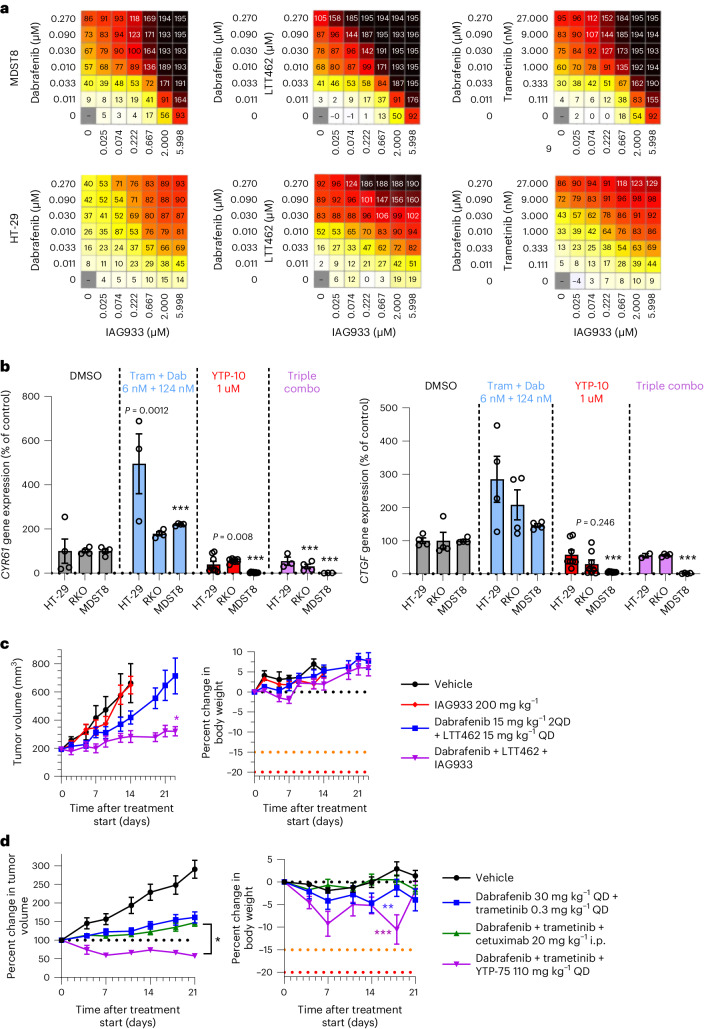
IAG933 shows a combination benefit with dabrafenib and other MAPK inhibitors in *BRAF*^V600E^-mutated cancer models. **a**, Antiproliferative activity dose matrices, 6-day readout; data represent the mean values of triplicates for IAG933 with dabrafenib, IAG933 with dabrafenib and LTT462 and IAG933 with dabrafenib and trametinib in HT-29 and MDST8 *BRAF*^V600E^-mutated CRC cells. **b**, *CCN1* and *CCN2* TEAD target gene expression in three cell lines after 24 h of treatment with dabrafenib (Dab) plus trametinib (Tram), YTP-10 or all three. Data are shown as mean ± s.e.m.; *n* = 4 for DMSO, *n* = 8 for YTP-10, *n* = 3 for dabrafenib/trametinib and *n* = 3 for the combination. Data were analyzed by one-way ANOVA, and comparisons to DMSO are shown (***P < 0.001; other *P* values are indicated on graphs). **c**, Antitumor efficacy of IAG933, dabrafenib + LTT462 or all three therapeutic agents combined in mouse HT-29 xenografts. Data are shown as mean ± s.e.m.; *n* = 5 per group. Data were analyzed by one-way ANOVA, and comparisons to the vehicle group are shown (*P = 0.0255). 2QD indicates twice daily. **d**, Antitumor efficacy of dabrafenib + trametinib ± cetuximab or YTP-75 in the 5238-HX mouse PDX model. Data are shown as mean ± s.e.m.; *n* = 10 for the vehicle group, *n* = 18 for the dabrafenib/trametinib group, *n* = 32 for the dabrafenib/trametinib + cetuximab group and *n* = 6 for the dabrafenib/trametinib + YTP-75 group. Data were analyzed by one-way ANOVA, and triple combinations were compared (*P = 0.083) or body weights were compared to the vehicle group (**P = 0.0471 and ***P < 0.0002). Source data

Because activating mutations in *BRAF* also drive oncogenic reliance on the MAPK pathway, we explored the combination potential of YTPs in the setting of *BRAF*^V600E^-mutant disease by combining IAG933 with the BRAF inhibitor dabrafenib, the MEK1/MEK2 inhibitor trametinib and/or the ERK1/ERK2 inhibitor LTT462. The combinations of dabrafenib + IAG933, dabrafenib + LTT462 + IAG933 and dabrafenib + trametinib + IAG933 showed benefit in short-term cell viability assays (Fig. 7a). Consistent with an adaptive role for TEAD activity on MAPK pathway inhibition, increased expression of TEAD-responsive genes was noted after dabrafenib + trametinib treatment without a YTP, which was prevented by concomitant TEAD inhibition with the IAG933 analog YTP-10 (Fig. 7b). Stronger antitumor responses were seen in the *BRAF*^V600E^-mutated CRC CDX model HT-29 with the triple combination of dabrafenib + LTT462 + IAG933 than with single-agent treatments (Fig. 7c). Similarly, the triple combination of dabrafenib + trametinib + YTP-75 showed stronger antitumor activity in the *BRAF*^V600E^-mutated CRC xenograft model 5238-HX than either dabrafenib + trametinib or dabrafenib + trametinib + cetuximab, resulting in a sustained tumor regression across the 21-day study period (Fig. 7d).

### TEAD and RAF/MAPK blockade benefit in non-*KRAS*^G12C^ PDAC

**Fig. 8:**
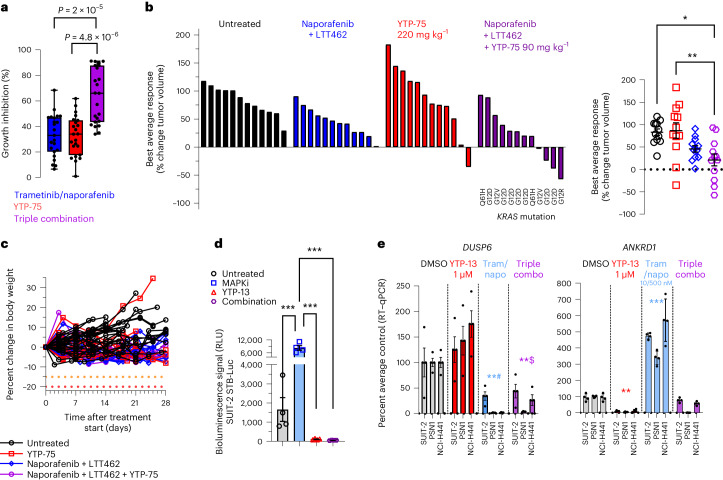
YTP blockade of MAPK pathway inhibitor-induced TEAD activation and increased antitumor response in pancreatic cancer models. **a**, Pharmacological sensitivity profiles across a panel of 23 pancreatic cancer cell lines using trametinib (10 nM), naporafenib (1 µM) and YTP-75 (1 µM). Cells were treated for 72 h. Significance was determined using a two-sided Welch’s *t*-test from 23 independent experiments. A box plot with whiskers extending to minimum and maximum is represented with individual data points. **b**, Best average responses (waterfall and scatter plots) in a panel of 12 non-*KRAS*^G12C^-mutated pancreatic cancer mouse PDX models treated with naporafenib (50 mg kg^−1^ twice daily) + LTT462 (15 mg kg^−1^ once daily), single-agent YTP-75 (220 mg kg^−1^ once daily) or naporafenib + LTT462 + YTP-75 (90 mg kg^−1^ once daily) in a 1 × 1 × 1 format (1 mouse × 1 model × 1 treatment). Significance was determined by a one-way ANOVA with a Tukey’s multiplicity adjustment and is represented on the scatter plot; *P = 0.0045 and **P = 0.0024. **c**, Good tolerability of the treatments reflected by body weight monitoring of animals implanted with 12 non-*KRAS*^G12C^-mutated pancreatic cancer mouse PDX models. **d**, TEAD activity in vitro reflected by the SUIT-2 STB-Luciferase cell bioluminescence reporter assay; data are shown as mean ± s.d.; *n* = 4 replicates after 48 h of treatment with naporafenib (500 nM) + trametinib (10 nM), YTP-13 (1 μM) or all three. Significance was determined by a one-way ANOVA with a Tukey’s multiplicity adjustment (***P < 0.0001); MAPKi, MAPK inhibitor. **e**, *DUSP6* and *ANKRD1* gene expression; data are shown as mean ± s.d.; *n* = 4 replicates in three cell lines treated for 48 h with naporafenib (500 nM) + trametinib (10 nM; Tram/napo), YTP-13 (1 μM) or all three. Data were analyzed by one-way ANOVA for all three cell lines, and comparisons to DMSO are shown (**^#^*P* = 0.002, **^$^*P* = 0.0051, **P = 0.0072 and ***P = 0.0005). Source data

Apart from the clinically targetable G12C variant, therapeutic suppression of KRAS-driven oncogenesis remains challenging^52^. To address non-*KRAS*^G12C^-mutant tumors, effective inhibition of downstream RAF, MEK and/or ERK effectors may offer potential therapeutic options. In this context, IAG933 could represent a promising combination opportunity, considering the encouraging combination outcomes achieved with mutant-specific inhibitors (Figs. 6 and 7 and Extended Data Fig. 10). We investigated this hypothesis in PDAC cells bearing various *KRAS* alleles. The addition of YTP-75 to trametinib plus the RAF inhibitor naporafenib significantly enhanced growth inhibition in a panel of 23 PDAC cell lines (Fig. 8a), consistent with results obtained from a mouse clinical trial^53^, including 12 PDAC PDXs with different *KRAS* mutations (7 G12D, 2 G12V, 2 Q61H and 1 G12R), where 8 models (66%) showed tumor regression or near stasis with the triple combination (Fig. 8b,c). Strong induction of TEAD transcriptional activity by trametinib + naporafenib was observed and prevented by YTP-13 cotreatment in a luciferase-based reporter system in SUIT-2 PDAC cells (Fig. 8d), and this triple combination was shown to inhibit both *DUSP6* and TEAD-responsive *ANKRD1* gene expression in a panel of three PDAC lines (Fig. 8e).

## Discussion

Here, we describe IAG933, a potent and selective small-molecule disruptor of the YAP/TAZ–TEAD PPI that shows promising preclinical activity, tolerability and PK properties. The observed effects after direct disruption of the YAP/TAZ–TEAD transcriptional complex in mesothelioma and Hippo-mutated tumor cells bearing *NF2* loss-of-function mutations or YAP–TAZ fusions were instrumental in establishing a clinical rationale for an ongoing first-in-human study (NCT04857372) of the oral compound IAG933 in these types of solid tumors. Of note, two other open-label, dose escalation studies for the allosteric LP-binding TEAD inhibitors VT3989 (NCT04665206, Vivace Therapeutics) and IK-930 (NCT05228015, Ikena Oncology) are also currently ongoing.

In principle, targeting the conserved TEAD coil site of the PPI region with IAG933 or a similar YTP compound could result in higher selectivity than a more ubiquitous structure such as the TEAD LP^26,27^. Moreover, the LP is subject to post-translational modifications and is not conserved among TEAD paralogs; consequently, we can expect variability in the affinity of LP binders among TEAD paralogs^28^. High-potency pan-TEAD targeting by YTPs could explain the deep and rapid cellular effects and antitumor responses obtained with IAG933, where the conserved inhibitory site likely prevents an easy bypass through poor inhibition or reactivation of TEAD paralogs. Comparison of forthcoming data from the three ongoing trials of TEAD inhibitors will be of great interest to establish any practical clinical differences between the two inhibitory modes and possibly serve as complementary targeting avenues to inhibit the YAP/TAZ–TEAD complex as a critical oncogenic node.

In addition to its direct oncogenic activity in Hippo pathway-altered cancers, YAP/TAZ–TEAD contributes to the intrinsic and acquired tumor resistance that undermines a wide variety of antitumor treatments^13,54,55^. Interest is therefore growing in the possibility of combining TEAD inhibitors with other agents to eradicate drug-tolerant persister cells. The development of targeted inhibitors for various components of the oncogenic MAPK signaling cascade has revolutionized treatment of many solid tumors, but these seldom affect remission on their own because resistance almost invariably develops by a wide variety of mechanisms, including gain of TEAD activity^3,5–8,10–13,43,44,48,56^. Although effective vertical combinations within the MAPK pathway can prolong responses, they remain limited by eventual relapse and challenging clinical on-target/in-pathway toxicity. TEAD inhibition may offer an orthogonal combination approach, with MAPK inhibitors benefiting from nonoverlapping toxicity profiles. In several preclinical models, we have observed enhanced TEAD activity and target gene transcription after treatment with MAPK-related inhibitors, including in *BRAF*^V600E^ CRC and in *KRAS*-mutant PDAC, consistent with a compensatory adaptation mechanism to MAPK blockade that could increase susceptibility to YAP/TEAD inhibition. Conversely, in the case of NSCLC, treatment with a KRAS^G12C^ inhibitor did not exhibit a distinct modulation of TEAD target gene expression. However, the combination of IAG933 with a KRAS^G12C^ inhibitor still demonstrated significant antitumor activity, which was accompanied by the induction of apoptosis. Hence, the success of these combinations with IAG933 could be attributed to multiple mechanisms. Further experiments are necessary to clarify the dynamics and distinct contributions in such YAP/TAZ–TEAD inhibitor combinations in specific cancer indications.

YAP/TAZ–TEAD inhibition was found to be the most effective pharmacological and genetic strategy to improve the response to KRAS^G12C^ inhibitors in our study and others^46,47^. Recently, allosteric TEAD inhibitors have been used to evaluate the activity of the combination of KRAS^G12C^ inhibitor and TEAD inhibitor^45–47^. Although the mechanisms of synergy appear to differ based on cancer model, sensitivity, intrinsic versus acquired resistance and different indications, we equally observe the combination of mutant-selective KRAS inhibitors with IAG933 ultimately promoting sustained antiproliferative and proapoptotic effects.

The preclinical data reported here reinforce the use of IAG933 as a combination partner with MAPK pathway-based drugs to increase the therapeutic opportunities in populations with high unmet medical need. Clinical trials of IAG933-based combinations are awaited following the ongoing single-agent first-in-human assessment in Hippo-driven tumors.

## Methods

The research performed in this report complies with Novartis relevant ethical regulations, and protocols were approved by the human samples ethical and animal welfare committee.

### Statistics and reproducibility

Statistical analyses of the data were conducted using R or GraphPad Prism. When assessed, the data met the assumptions of the statistical tests used (normality and equal variances). In cases when not tested, and data distribution was assumed to be normal, data distribution (individual data points) is shown. We verified that experiments were reproducible; most experiments were performed at least twice with similar results and often in different cell lines or tumor models. Sample sizes were determined based on the frequently used number of experimental replicates in standard experiments or the available literature rather than on sample size calculations. The sample size was not specifically selected to enable statistical analysis, and no statistical method was used to predetermine sample size. No data were excluded from analyses. Sex of tumor models was not considered in the study designs. For mouse or rat in vivo experiments, when tumors reached a volume of approximately 150–250 mm^3^, animals were randomized based on tumor size and assigned to different experimental groups. Investigators were blinded to group allocation during genomics data processing. For other experiments, no blinding was applied.

### Crystal structure determination

Protein sample expression and purification of human TEAD3^218–435^ for crystallization have been described previously^24^. Crystals of TEAD3^218–435^ for inhibitor soaking were grown at 293 K using the sitting drop vapor diffusion method. Purified TEAD3^218–435^ at 6.8 mg ml^–1^ in 25 mM Tris (pH 8), 250 mM NaCl, 1 mM TCEP and 5% glycerol was mixed with an equal volume of reservoir solution (1.0 µl + 1.0 µl) composed of 50 mM magnesium chloride hexahydrate, 0.1 M HEPES (pH 7.5) and 30% PEG monomethyl-ether 550. Crystals appeared over several days and were soaked in reservoir solution containing 20% glycerol and 5 mM IAG933 for 5 h. The soaked crystals were flash cooled in liquid nitrogen for X-ray data collection. X-ray diffraction data were collected at the Swiss Light Source (beamline X10SA) using a Pilatus pixel detector. The data were processed with autoPROC^57^ using the default pipeline, which includes XDS, Truncate, Aimless and STARANISO (Global Phasing). Analysis by STARANISO revealed that diffraction data were anisotropic, with estimated diffraction limits for reciprocal space directions of 1.90 Å along a*, 2.37 Å along b* and 1.92 Å along c*. For the final round of data processing with STARANISO, the resolution was set manually to 1.96 Å and resulted in an anisotropic corrected data set for further analysis. The structure was solved by molecular replacement with PHASER^58^ using as a search model the coordinates of previously solved in-house structures of TEAD3^218–435^. The software programs COOT^59^ and BUSTER (version 2.11.8, Global Phasing) were used for iterative rounds of model building and structure refinement. PyMol (retrieved from http://www.pymol.org/pymol) was used for structural visualization and figure preparation. The refined coordinates of the complex structure have been deposited in the Protein Data Bank (www.wwpdb.org) under accession code 8P0M. Four complexes of TEAD3^218–435^ bound to IAG933 were found within the asymmetric unit of the crystal, each of them displaying strong initial difference electron density for the bound inhibitor. For all further analysis and figures, the complex with protein chain A was used.

### Compound synthesis

The syntheses of the following compounds were described in previous reports: YTP-10 (ref. ^25^), YTP-17 (ref. ^25^), YTP-3 (ref. ^24^) and YTP-3a (ref. ^24^). The syntheses of IAG933, YTP-75 and YTP-32 were described in a published patent (patent WO2021/186324, Biaryl derivatives as YAP/TAZ–TEAD protein–protein interaction inhibitors, 2021).

### Surface plasmon resonance assay

Surface plasmon resonance assay measurements were acquired with human TEAD1^209–426^, TEAD2^221–447^, TEAD3^218–435^ and TEAD4^217–434^ as previously described^28^. The four *N*-biotinylated TEAD proteins were tagged with AviTag and immobilized on sensor chips, and the binding of different concentrations of YTP-3 and YTP-32 was measured at 298 K. The data were globally fitted with a 1:1 interaction model using Biacore T200 evaluation software (Cytivia) to determine the dissociation constants (*K*_d_) measured at equilibrium.

### TR-FRET assays

Different compounds were tested in a TR-FRET assay as previously reported^24^. The lipid-binder compounds (K-975 and VT104) targeting the myristate/palmitoyl pocket are inactive in TR-FRET because the TEAD4 protein used in this assay is fully acylated.

### Cell lines and cell culture

All human cancer cell lines are part of the Broad-Novartis Cancer Cell Line Encyclopedia (https://sites.broadinstitute.org/ccle) and were cultured with medium as stated in the database. Novartis Biomedical Research, Oncology Department, maintains a cell line bank with a strict protocol in place to ensure the control and quality of all cell lines. All cell lines are maintained internally, with direct oversight and management of their handling and storage. To ensure integrity of the cell lines, the bank implements regular single nucleotide polymorphism genotyping. It is mandatory for all labs within Novartis Oncology to use the cell line bank stocks when initiating an experiment. This requirement ensures uniformity and consistency across different research projects and prevents any potential variations that may arise from using different cell line sources. Reagents were purchased at BioConcept. Cells were maintained at 37 °C with 5% CO_2_. mRNA expression and DNA alteration data from in vitro cultures were previously generated^56^. Nonhuman cell lines, purchased from ATCC, were cultured at 37 °C and 5% CO_2_ and were split twice a week (MDCK (CCL-34), NIH-3T3 (CRL-1658), CT-26 (CRL-2638) and RAT-1 (CRL-2210)). These cell lines were maintained in high-glucose DMEM (4.5 g l^–1^), 10% fetal calf serum (FCS), 2 mM l-glutamine, 1 mM sodium pyruvate and 0.1 mM nonessential amino acids (BioConcept), except for the CT-26 cell line that was cultured with RPMI-1640, 10% FCS, 4 mM l-glutamine, 1 mM sodium pyruvate and 0.1 mM nonessential amino acids (BioConcept) and MDCK cell lines cultured in EMEM, 10% FCS, 4 mM l-glutamine, 1 mM sodium pyruvate and 0.1 mM nonessential amino acids (BioConcept). Cell lines implanted in vivo were confirmed to be *Mycoplasma* free and pathogen free with IMPACT-8, IDEXX BioAnalytics.

### Reporter gene and cell proliferation profiling assays

Cellular reporter gene and proliferation assays were performed as previously described^24^. The reporter gene assay used NCI-H2052 and SF-268 cell lines stably expressing the previously described TEAD-responsive MCAT_Luc reporter^60^.

### Cell engineering and cell line derivatives

MSTO-211H or SUIT-2 cells were engineered to express STB-Luc firefly luciferase under the control of a minimal promoter, to which six repeats of the TEAD-responsive element 5′-GCA GGA ATG CAG GGA ATG-3′ were added. These were included in a pLenti6 backbone that expressed luciferase reporter gene *luc2P* (*Photinus pyralis*). Cells were infected and then selected with blasticidin.

NCI-H2052 cells expressing doxycycline-inducible shRNAs were generated by lentiviral transduction of a modified pLKO-TET-ON plasmid, followed by puromycin selection (1 μg ml^–1^). shRNA sequences used for single validation studies were described previously^61^. The shYAP1.2371 21-mer guide sequence was 5′-TTATATGGAAATTGTCTCATG-3′, and the passenger–loop–guide sequence was 5′-CATGAGACAATTTCCATATAATTCAAGAGATTATATGGAAATTGTCTCATG-3′.

Secreted *Gaussia* luciferase (GLuc) was expressed in MSTO-211H cells using a lentiplasmid vector Lenti-UBC-GLuc-T2A-Puro (Targeted Systems). Stable expression was under control of the *UBC* promoter and puromycin resistance. MSTO-211H cells were infected and selected before xenograft implantation.

The fusion gene cellular models from the NIH-3T3 cell line were generated via lentivirus from a pXP1510 backbone^62^ in which the synthesized sequences (GeneArt) of wild-type *YAP*, *YAP*–*MAML2* or *TAZ*–*CAMTA1* cDNAs were integrated. V5 tag was introduced via mutagenesis of pXP1510 *YAP*–*MAML2* and *TAZ*–*CAMTA1* using a QuikChange XL site-directed mutagenesis kit (Stratagene, 200516) and the following primers:

V5_TAZ_CAMTA1_forward: 5′-cgccaccatgGGTAAGCCTATCCCTAACCCTCTCCTCGGTCTCGATTCTACGaatcccgcctc-3′

V5_ TAZ_CAMTA1_reverse:

5′-gaggcgggattCGTAGAATCGAGACCGAGGAGAGGGTTAGGGATAGGCTTACCcatggtggcg-3′

V5_YAP_MAML2_forward:

5′-cgccaccatgGGTAAGCCTATCCCTAACCCTCTCCTCGGTCTCGATTCTACGgatcccggccaac-3′

V5_YAP_MAML2_reverse:

5′-gttggccgggatcCGTAGAATCGAGACCGAGGAGAGGGTTAGGGATAGGCTTACCcatggtggcg-3′

### Short-term cell proliferation assays

The effect of compounds on cell proliferation was assessed by quantifying cellular reducing capacity using a resazurin sodium salt dye reduction assay^63^. Briefly, cells were seeded at 750 cells per well into black-wall, clear-bottom 384-well plates (Corning) and incubated overnight at 37 °C before addition of serial compound dilutions or vehicle control (0.1% DMSO) using a HP300 digital dispenser (TECAN). After incubation for 72 h at 37 °C, compound-mediated modulation of cell viability was assessed as follows. After addition of a 1:5 (vol/vol) aliquot of 5× resazurin stock solution (resazurin sodium salt (Sigma) dissolved at 3.25 µg ml^–1^ in PBS), cell plates were incubated for an additional 4 h at 37 °C and 5% CO_2_. Following equilibration of the plates at room temperature for 15 min, the levels of resorufin (the reduced form of resazurin) were quantified using a M200 multipurpose plate reader (TECAN), with fluorescence excitation and emission wavelengths set to 544 nm and 590 nm, respectively. To enable differentiation of cytotoxic and cytostatic compound effects, the number of viable cells on the day of compound addition (day 0) was also assessed in a separate cell plate and used to calculate the extent of cell viability suppression as follows. The assay background value determined in wells containing medium with no cells was subtracted from all data points. The extent of growth inhibition and potential cell killing was assessed by comparing the resorufin levels in compound-treated cells to those present at the time of compound addition. To this end, the following conditional concept was programmatically applied in HELIOS^64^, an in-house software package that applies a multistep decision tree to arrive at optimal concentration response curve fits to calculate percent growth for each compound-treated well: percent growth = [(*T* – *V*_0_)/*V*_0_] × 100 when *T* < *V*_0_, and percent growth = [(*T* – *V*_0_)/(*V* – *V*_0_)] × 100 when *T* ≥ *V*_0_. Here, *V*_0_ is the viability level at time of compound addition, whereas *V* and *T* represent vehicle control and compound-treated viability levels, respectively, at the end of the compound incubation; 100%, 0% and −100% signify absence of growth inhibition, growth stasis and complete cell killing, respectively. Compound concentrations leading to GI_50_ and residual cell viability at the highest tested compound concentration (data(*C*_max_), expressed in percent) were routinely calculated.

### Immunohistochemistry and fluorescence microscopy of cultured cells

NCI-H2052-MCAT-luc cells^24^ (2,500 cells per well) were plated into black, clear-bottom 384-well plates (Becton Dickinson) and incubated overnight at 37 °C before noncontact dispensing of compounds or vehicle control (0.1% DMSO) using a HP300 digital dispenser (TECAN). Cells were fixed by addition of an equal volume of 7.4% formaldehyde. After 10 min, plates were washed once with PBS using a BioTek ELx450 Plate Washer (Agilent) and permeabilized by incubating for 15 min with Triton X-100 (0.5% (vol/vol) in PBS), followed by three washes with PBS. Cells were then blocked for 1 h with Odyssey Blocking Buffer (LI-COR), followed by an overnight incubation at 4 °C with rabbit anti-YAP (EP1674Y, Abcam) and mouse anti-TEF-1 (TEAD1; Becton Dickinson), both diluted 1:500 in Odyssey Blocking Buffer. Following three washes with PBS, cells were incubated for 1 h at room temperature with Alexa Fluor 488-labeled goat anti-rabbit (Invitrogen) and Alexa Fluor 568-labeled goat anti-mouse (Invitrogen), both diluted 1:500 in Odyssey Blocking Buffer containing 1 μg ml^–1^ Hoechst 33342. Following three final washes with PBS, nuclei, YAP and TEF-1 were imaged on a Cellomics ArrayScan VTI high-content imager (Thermo Fisher) in widefield mode using a ×10/0.3-NA objective on the first three channels of the BGFRF dichroic filter set with LED excitation at 386, 485 and 549 nm and emission at 440, 524 and 593 nm, respectively.

### Tumor immunohistochemistry

Tumor samples were fixed overnight in 4% paraformaldehyde, rinsed in PBS, dehydrated in ascending baths of ethanol and finally embedded in paraffin. Paraffin sections were then cut on a rotary microtome (3 μm, Mikrom International), spread in a 45 °C water bath, mounted on microscope slides (Thermo Scientific) and air dried in an oven at 37 °C overnight. After drying, 3-μm tissue section slides were stained on a BondRX platform (Leica Biosystems), as per the manufacturer’s instructions, using Epitope Retrieval 2 conditions for 20 minutes at 100 °C and a Refine DAB kit (Leica Biosystems) as an amplification system for all tested markers. Primary antibodies used were either anti-Ki67 clone SP6 (Neomarkers, RM9106) or cleaved caspase-3 (Cell Signaling Technology, 9661) at a 1:2,000 dilution or cleaved PARP (Cell Signaling Technology, 9541) at a 1:100 dilution. After dehydration and coverslipping, slides were then scanned with a Scanscope XT slide scanner (Aperio). Corresponding digital slides were then quantified using the HALO Area Quantification algorithm (Indica Labs) for cleaved caspase-3 expression and the HALO CytoNuclear algorithm (Indica Labs) for cleaved PARP and Ki67 expression. Results were expressed as the percentage of positive pixels per total pixels for cleaved caspase-3 and percentage of positive cells per total cells for cleaved PARP and Ki67.

### Selectivity assessment in colony formation assays with clones derived from SF-268 cells

The SF-268 cell line was engineered, and a clone bearing a double mutation in *TEAD1* (V406A/E408A) was established as follows. The targeting sequence of *TEAD1* (gtgcattcgctgtttcaaat) was cloned into the pNGx_006 vector (pUC/ori, U6 promoter for tracrRNA/chimera, CMV promoter for SPyCas9 and puromycin selection). SF-268 cells (2 × 10^5^) were electroporated with 1.5 μg of pNGx_006_sgTEAD1 and 0.5 μg of single-stranded oligonucleotide for *TEAD1*^V406A^ and *TEAD1*^E408^ (ttaacaggtggtaacaaacagggatacacaagaaactctactctgcatggcctgtgcattcgctgtttcaaatagtgaacacggagcacaacatcatatttacaggcttgtaaaggactg) using a Neon Transfection System (Invitrogen) with the following parameters: voltage 1,300 V, pulse 20 ms and pulse number 2. Single clones were seeded after puromycin selection and characterized by Sanger sequencing. For the colony formation assay, SF-268 clones 18 and 23 were seeded at low density (1,000 cells per well in six-well plates) 24 h before treatment. Test compound (IAG933) was distributed into the assay plates in a five-point threefold serial dilution starting at a top concentration of 10 μM. DMSO was used as a control, and DMSO content was normalized to the highest volume in all compound-treated wells. Medium containing compound was renewed twice a week. After an incubation period of 11 days under regular cell culture conditions (37 °C, 5% CO_2_), cells were fixed with 3.7% formaldehyde for 10 min, and colonies were stained with crystal violet.

### Quantitative PCR with reverse transcription for evaluation of target gene inhibition

RNA from cell lines or tissues was extracted using an RNeasy Mini kit (Qiagen, 74106). RNA concentration and purity were determined using a NanoDrop (Thermo Fisher). A duplex quantitative PCR with reverse transcription assay was performed in 384-well plates (Applied Biosystems, 4309849) on a QuantStudio 6 Flex device (Applied Biosystems) using an iTaq Universal Probes One-Step kit (Bio-Rad, 172-5140). Ten nanograms of RNA was mixed with relevant TaqMan probes to detect an *ACTB* probe for normalization. The cycles used were 50 °C for 10 min for reverse transcription, 95 °C for 3 min, followed by 40 cycles of 95 °C for 15 s and 60 °C for 1 min. *ACTB* cycling threshold (*C*_t_) values were subtracted from those of the evaluated gene and *C*_t_ values obtained for each well to calculate the ∆*C*_t_. The $2^{\Delta C_{\rm t}}$ value was calculated for each well. Averages for duplicate or triplicate samples were calculated for each data point. Eventually, the percentage of RNA expression from compound-treated samples was calculated in Excel (Microsoft) relative to RNA expression from vehicle-treated samples.

The following Taqman assays (Thermo Fisher Scientific) were used: human *CCN1* (6-FAM/ZEN/3′ IBFQ): Hs.PT.58.3413227.g; human *ANKRD1* (6-FAM/ZEN/3′ IBFQ): Hs.PT.58.14671023; human *CCN2* (6-FAM/ZEN/3′ IBFQ): Hs.PT.58.14485164.g; human *AMOTL2* (6-FAM/ZEN/3′ IBFQ): Hs.PT.58.39983582; human *BCL2L1* (6-FAM/ZEN/3′ IBFQ): Hs.PT.56a.14668121; human *MCL1* (6-FAM/ZEN/3′ IBFQ): Hs.PT.58.26560856; human *DUSP6* (6-FAM/ZEN/3′ IBFQ): Hs04329643_s1; human *SPYR4* (6-FAM/ZEN/3′ IBFQ): Hs01935412_s1; mouse *Ccn1* (6-FAM/ZEN/3′ IBFQ): Mm00487499_g1; mouse *Ankrd1* (6-FAM/ZEN/3′ IBFQ): Mm00496512_m1; mouse *Gapdh* (VIC-MGB): Mm99999915_g1; rat *Ankrd1* (6-FAM/ZEN/3′ IBFQ): Rn00566329_m1; rat *Ccn2* (6-FAM/ZEN/3′ IBFQ): Rn01537279_g1; rat *Actb1* (VIC-MGB): Rn00667869_m1; dog *Ccn1* (6-FAM/ZEN/3′ IBFQ): AJX01BF; dog *Ankrd1* (6-FAM/ZEN/3′ IBFQ): Cf02662722_m1; dog *Ccn2* (6-FAM/ZEN/3′ IBFQ): Cf02641589_m1; dog *Gapdh* (VIC-MGB): Cf02641589_m1.

The following human *ACTB* reagents were obtained separately from Integrated DNA Technologies: *ACTB* reverse primer: 5′-CCA GTG GTA CGG CCA GAG G-3′; *ACTB* forward primer: 5′-GCG AGA AGA TGA CCC AGA TC-3′; *ACTB* labeled probe: 5′-VIC-CCA GCC ATG TAC GTT GCT ATC CAG GC-TAMRA-3′.

### Experimental animals

All animal studies were conducted in accordance with ethics and procedures covered by permits BS-1763 and BS-1767, respectively, whether the model was induced ectopically or orthotopically, issued by the Kantonales Veterinäramt Basel-Stadt and in strict adherence to guidelines of the Eidgenössisches Tierschutzgesetz and the Eidgenössische Tierschutzverordnung, Switzerland. Female nude rats (Crl:NIH-*Foxn1*^*rnu*^ homozygous) and female nude mice (Crl:NU(NCr)-*Foxn1*^*nu*^ homozygous) were purchased from CRL Germany. Female SCID mice (C.B-*Igh-1*^*b*^/IcrTac-*Prkdc*^*scid*^) were purchased from Taconic Europe. Mice were maintained under optimal hygiene conditions in individually ventilated cages under 12-h dark/12-h light conditions and controlled temperature (21–22 °C) and humidity (between 50 and 55%) and had access to sterilized food and water ad libitum. Tumor volumes and body weights were measured two to four times weekly. The maximal tumor size/burden permitted is 1,500 mm^3^ and was not exceeded. Conditional survival was defined as a maximum estimated tumor diameter of 1.5 cm or when mice showed symptoms of morbidity/moribundity or body weight loss of >15%.

### Generation of xenograft tumor models in mice and rats

Subcutaneous tumors from cell lines were induced by injecting cells in 200 μl of HBSS containing 50% BD Matrigel subcutaneously in the flank of animals (5 millions cells per animal except for NCI-H226 at 2.5 millions and NCI-H1975 at 2 millions cells per animal). Nude mice were mainly used, except for two CDX models for which SCID mice were used (MSTO-211H and NCI-N87). For nude rat studies, animals were irradiated 24 h before MSTO-211H cell injection using an X-ray irradiator RS2000 at 5 Gy over 4 min. Irradiation was performed on conscious animals. Orthotopic tumors were induced by injecting 2 million MSTO-211H cells in 50 μl of HBSS through the fourth intercostal space in the pleural cavity. For all PDX models or the serially transplanted CDX originating from NCI-H2052 cells, approximately 1–2 mm^3^ tissue fragments were implanted subcutaneously with 50% (vol/vol) Matrigel (354234, Corning) into the flank region of mice using a trocar. Successfully engrafted tumor models were then passaged once and banked. Tumor material on flanks was collected in PBS and kept on wet ice for engraftment within 3 h after resection or slow frozen. Necrotic and supporting tissues were carefully removed using a surgical blade.

### Animal treatments

Most compounds were administered at the indicated doses by oral gavage with the following formulations. IAG933 was formulated in 0.5% methylcellulose and 0.1% Tween-80 in 100 mM phosphate buffer (pH adjusted to 8). VT104 and K-975 were formulated in 100% Maisine CC (Gattfossé). YTP-75 was formulated in 30% PEG300 and 50 mM acetate buffer (pH adjusted to 5.5). YTP-13 was formulated in 5% PEG300 and 50 mM acetate buffer (pH adjusted to 4.8). LTT462, dabrafenib and trametinib were formulated in 20% MEPC4 in water. JDQ443, TNO155, osimertinib and capmatinib were formulated in 0.5% methylcellulose and 0.1% Tween-80 in water. Other compounds were administered by intraperitoneal injection. Antibodies to trastuzumab and cetuximab and MRTX1133 compound were formulated in Dexolve (Cyclolab).

### PD in vivo studies

Animals were assigned into groups of *n* = 3–5 per time point and treatment. Blood, plasma and tumor samples for PK and PD analyses were collected. Blood samples were collected on ice and stored at –20 °C until further processing. Plasma and tumor samples were snap-frozen on dry ice and stored frozen at –80 °C until further processing. The in vivo TEAD reporter assay was performed with the MSTO-211H STB-Luc orthotopic pleural mesothelioma tumor model. For each measurement, mice were injected intraperitoneally with luciferin (150 mg kg^−1^). Exactly 20 min later, the mice were imaged with an IVIS Spectrum (PerkinElmer) while conscious and restrained for less than 1 min.

### In vivo efficacy studies

Mesothelioma PDX studies were conducted at Charles River, Germany, and the PDAC PDX mouse clinical trial study was conducted at Southern Texas Accelerated Research Therapeutics (XenoSTART). All other experiments were conducted internally. Treatment was initiated when the tumors engrafted in the flank were at least 100 mm^3^, and random enrollment was applied. Efficacy studies, tumor response and relapse were reported with the measures of tumor volumes at the start of treatment. For efficacy studies on ectopic models, animals were randomized into treatment groups based on tumor volume. Tumor size was measured using a caliper and calculated using the formula length × width^2^ × π/6. As a measure of efficacy, the percent *T*/*C* value was sometimes calculated at the end of the experiment or at best response using the formula (Δtumor volume treated/Δtumor volume control) × 100. In the case of tumor regression, the tumor response was quantified using the formula –(Δtumor volume treated/tumor volume treated at start) × 100. Statistical analyses were performed using GraphPad Prism. For efficacy studies on pleural orthotopic models, viable tumor burden was assessed by measurements of GLuc from 20 μl of blood collected in microvette EDTA-coated tubes, and samples were stored at –20 C. Coelentrazine (Nanolight) substrate solution was added (100 μl of a 100 mM solution) to each well of 96-well white plates, and 5 μl of blood was added in triplicate. Bioluminescence was measured with a CentroXS LB960 Luminometer (Berthold Technologies) for 2 s.

### Bioanalytical method for detection of compounds in blood, plasma and tumors

Concentrations of IAG933 and YTP-75 in total blood, plasma and tissues were determined by a ultrahigh performance liquid chromatography–tandem mass spectrometry (UPLC–MS/MS) assay. Frozen tissue samples were pulverized to powder using CryoPrep according to manufacturer’s instructions (Covaris) or homogenized in an equal volume of HPLC water (water for chromatography, Merck) using the Fast Prep-24 system (MP Biomedicals). Samples (about 25 mg, exact weight collected) of blood, plasma or tissue (in the form of powder or homogenate) were mixed with 25 µl of internal standard (1 µg ml^–1^) and extracted by the addition of 200 µl of acetonitrile to precipitate proteins. After sonication for 5 min, samples were centrifuged, and supernatants (70 µl) were mixed with 60 µl of HPLC water before the analysis of 5-µl aliquots by UPLC–MS/MS. Samples were injected onto a reverse-phase column (Waters) using formic acid in water and formic acid in acetonitrile as mobile phases. The column eluent was directly introduced into the ion source of the triple quadrupole mass spectrometer (Waters). Electrospray positive ionization multiple reaction monitoring was used for MS/MS detection of the analyte. PK parameters were calculated from the mean values with the linear trapezoidal rule by using a noncompartmental model for extravascular dosing (Phoenix Certara).

### Combination assays in matrix format

The effect of compound combinations on cell proliferation was assessed by ATP quantification using CellTiter-Glo reagent (Promega). Cells were seeded at 300–700 cells per well in white-walled, clear-bottomed 384-well plates (Greiner) and incubated overnight at 37 °C before the addition of serial compound dilutions or vehicle control in a matrix format using an HP300 digital dispenser (TECAN), and treatments were applied in triplicate. After incubation for 5–7 days in the presence of compounds, cell viability was monitored using CellTiter-Glo following the supplier’s instructions. Data were analyzed using the in-house program Combination Analysis Module. To enable differentiation of cytotoxic from cytostatic compound effects, the number of viable cells on the day of compound addition (day 0) was also assessed in a separate cell plate and used to calculate the extent of cell viability suppression. Depending on whether the CellTiter-Glo signal for a given point in the concentration matrix was above or below day 0, the latter suggesting cell death due to compound treatment, a ‘growth inhibition’ (*GI*) value was calculated as follows: *T* < *D*_0_: *GI* = 100 × {1 – [(*S* – *D*_0_)/*D*_0_]}; *T* ≥ *D*_0_: *GI* = 100 × [1 – (*S* – *D*_0_)/(*V* *–* *D*_0_)], where *D*_0_ is day 0, *V* is vehicle control, and *S* is signal. This formula leads to a scale where 0 corresponds to no compound effect compared to vehicle, 100 corresponds to growth arrest (that is, signal on endpoint equal to signal on day 0), and 200 corresponds to complete cell killing. In Fig. 6a, threefold dilutions were used for IAG933 starting from 5.595 µM for NSCLC and 3 µM for CRC cell lines and fourfold dilutions for JDQ443 starting with 1.6 µM as the highest compound concentrations.

### Long-term confluency assays

NCI-H2052, NCI-H226, MSTO-211H, SNU-216, NCI-H2170, NCI-H2122, NCI-H358, AsPC-1, HPAF-II and GP2D cell lines were plated in 96-well plates and incubated at 37 °C for 24 to 48 h before the addition of compounds at the indicated concentrations. Confluency was monitored at the indicated time points and quantified by Incucyte live-cell imaging technology.

### Live-cell monitoring of apoptosis

Cells were plated in 96-well plates and subjected to double thymidine block to arrest cells in S phase synchronization. Briefly, cells were plated in 96-well plates in growth medium and incubated overnight. The next day, thymidine was added to a final concentration of 2 mM for 24 h. Thymidine was removed by washing cells two times with growth medium. Release was induced by the addition of 100 µl per well of growth medium for 8 h. Following the release step, a second thymidine block was performed by repeated addition of 2 mM thymidine (final concentration) for an additional 24 h. After the second thymidine block, cells were washed twice with growth medium, 100 µl of growth medium was added, and treatment was applied. After release from S phase synchronization, compounds were added at the specified concentrations as well as Incucyte Caspase-3/Caspase-7 Green Dye (4440) and Incucyte Cytotox Red Dye (4632), following the supplier’s instructions. Caspase-3/caspase-7 activity (apoptosis), Cytotox staining (cell death) and cell number were monitored using Incucyte S3 during a 96-h period using the cell-by-cell analysis module to determine the percentage of cells undergoing apoptotic cell death (being positive for both caspase-3/caspase-7 and Cytotox reporters) over time.

### Immunoblotting

Lysates (10–200 µg per lane) were subjected to SDS–PAGE (4–12% NUPAGE gels and MES running buffer) followed by wet western blotting. Membranes were blocked for 1 h in 5% skim milk/PBS/0.1% Tween-20, and the following antibodies were used and diluted as indicated by the manufacturer in blocking buffer at 4 °C overnight: anti-YAP (D8H1X; Cell Signaling Technology, 14074S), anti-TAZ (Cell Signaling Technology, 4883), anti-TEF-1 (BD, 610922), pan-TEAD D3F7L (Cell Signaling Technology, 13295), anti-vinculin (Sigma, V9131), anti-V5-Tag (Cell Signaling Technology, 80076), anti-KRAS (3B10-2F2; Novus, H00003845-M01), anti-RSK1/RSK2/RSK3 (32D7; Cell Signaling Technology, 9355), anti-phospho-MAPK (Thr 202/Tyr 204; Cell Signaling Technology, 9101), anti-phospho-RSK3 (T356/S360; Cell Signaling Technology, 9348), anti- MAPK (Cell Signaling Technology, 9102), anti-MCL1 (ENZO, ADI-AAP-240-F), anti-BCL-xL (Cell Signaling Technology, 2764), anti-BMF (Cell Signaling Technology, 50542), anti-cleaved PARP (Cell Signaling Technology, 5625), anti-BIM (Cell Signaling Technology, 2933), anti-GAPDH (Cell Signaling Technology, 8884), anti-actin clone C4 (Millipore, MAB1501) and anti-β-tubulin (Sigma-Merck, T4026).

Secondary antibodies used were anti-mouse or anti-rabbit (Cell Signaling Technology, 7074 and 7076), Veriblot-HRP (Abcam, ab131366), anti-rabbit-HRP (Dako, P0448) or anti-mouse-HRP (Amersham GE Healthcare, NA931). Chemiluminescent signal was acquired using a Fusion-FX7 edge camera (Vilber Lourmat)

### Coimmunoprecipitation

For pan-TEAD immunoprecipitation, cells were lysed in NP-40 buffer (Invitrogen, FNN0021), 6 mg ml^–1^ sodium pyrophosphate and phosSTOP and protease inhibitor cocktail (Roche). Protein lysates were incubated with pan-TEAD D3F7L antibody (Cell Signaling Technology, 13295) for 16 h with rotation at 4 °C and then incubated with 1∶10 Dynabeads (Invitrogen, 10004D) for 1.5 h with rotation at 4 °C. Immunoprecipitates were then washed three times with NP-40 and eluted with Laemmli sample buffer (Bio-Rad, 310010517) by incubation at 95 °C for 5 min or with LDS sample buffer (Invitrogen, NP0007 + NP0009) by incubating at 70 °C for 15 min.

For BMF and BIM immunoprecipitations, HCC1171 cells were lysed in M-PER (Thermo, 78501) supplemented with complete protease inhibitor (Roche Diagnostics, 11 836 145001) and PhosStop tablets (Roche Diagnostics, 04 906 837001). Total lysates (200 µg) were incubated with anti-BMF (Cell Signaling Technology, 50542) 1:200 or anti-BIM (Cell Signaling Technology, 2933) 1:200 for 3 h on ice, and 30 µl per sample of Protein G Dynabeads was added and incubated for 1 h at 4 °C on a rotating wheel. Samples were washed three times with 900 µl of lysis buffer, beads were recovered using a magnetic stand, and 40 µl per sample of SDS sample buffer was added to elute precipitated proteins. Enriched samples were then subjected to SDS–PAGE and western blotting.

### RNA-seq and data analysis

Cell lines were treated as indicated, and three biological replicates were collected for each condition. Total RNA was extracted using a Qiagen RNeasy Mini kit. Library construction was performed using a RiboZero RNA-seq kit (Qiagen) and a TruSeq RNA Sample Prep kit v2 (Illumina). Sequencing was performed on a HiSeq 4000 machine (2 × 76 base pair (bp) reads). We obtained more than 30 million raw reads per sample. The quality of raw data was evaluated using RSeQC (v3.0.0), and no read trimming was performed. Transcript quantification was performed using PISCES v.2018.04.1 (ref. ^65^) and referenced to the hg38 human genome. Differential expression analysis versus DMSO-treated cells was performed using DESeq2. Gene functional annotation was performed with R Bioconductor and the clusterProfiler package (v2.10.0).

### ChIP–seq and data analysis

ChIP–seq was performed essentially as previously described^31^. Briefly, cells were cross-linked for 10 min in 1% formaldehyde (Sigma), followed by quenching with 0.125 M glycine (Sigma). Cells were lysed and collected in ChIP buffer (100 mM Tris (pH 8.6), 0.3% SDS, 1.7% Triton X-100 and 5 mM EDTA), and chromatin was sonicated using a Bioruptor Pico (Diagenode) to obtain fragments of average 200–500 bp in size. One hundred micrograms of DNA for transcription factors and 10 μg of DNA for histone marks were used per immunoprecipitation (measured as DNA abundance) and incubated for 16 h with the following antibodies: YAP (Abcam, ab52771), TEAD4 (Abcam, ab58310), H3K27ac (Cell Signaling Technology, 8173) and H3K4me1 (Cell Signaling Technology, 5326). Libraries for ChIP–seq were generated using the Ovation Ultralow Library System V2 (NuGEN), and barcodes were added using New England Biolabs Next Multiplex Oligos for Illumina (index primers set 1) according to the manufacturer’s recommendations. Sequencing was performed on a NovaSeq (Illumina). On average, 60 millions reads per sample were obtained, with a minimum of 25 million uniquely mapped reads. Peak calling was performed using MACS2 version 2.2.7.1 with default parameters. All samples passed the ENCODE quality control pipeline using MultiQC version 1.6.

### TT-seq and data analysis

TT-seq was performed as previously described^66^. MSTO-211H cells were grown as an adherent monolayer under regular cell culture conditions (37 °C, 5% CO_2_) using RPMI-1640 medium supplemented with 10% fetal bovine serum, 2 mM l-glutamine, 1 mM sodium pyruvate, 0.1 mM of each nonessential amino acid and 10 mM HEPES. Briefly, for each replicate, approximately 10 million MSTO-211H cells were treated for 1 h with solvent DMSO (control) or for 1 h or 6 h with 250 nM YTP-75. Five minutes before the treatment endpoint, labeling was performed by adding 500 µM 4-thiouridine (4sU; Sigma-Aldrich) for 5 min at 37 °C and 5% CO_2_. Liquid was discarded, and total RNA was extracted using QIAzol lysis reagent (Qiagen). At this point, 4sU-labeled RNA from *Drosophila melanogaster* S2 cells was added as a spike-in (10% of total amount). Three hundred micrograms of RNA was sonicated to generate fragments of <1.5 kbp using 1.5-ml TPX microtubes (Diagenode, C30010010) on a Bioruptor Plus sonication device (Diagenode) at high settings for one cycle of 30 s ON/30 s OFF. 4sU-labeled and fragmented RNA was biotinylated using EZ-Link HPDP-Biotin (Thermo Scientific) and precipitated and separated using Streptavidin beads (Invitrogen). RNA was then purified using an RNA Clean and Concentrator kit 5 (Zymo Research), and integrity and concentration was assessed before library preparation. Libraries were prepared using a New England Biolabs Next Ultra II Directional RNA library prep kit for Illumina according to the protocol. Each replicate was sequenced with ∼60 millions reads per sample with two replicates for each condition on a HiSeq2500 device (Illumina).

Fastq files were mapped to hg38 using the STAR (2.5.2a) aligner in paired-end mode. The resulting bam files were sorted using Samtools (1.12). For strand-specific coverage, the alignments were split into two bam files using Samtools (1.12). Alignments to the forward strand were selected by Samtools view -b -f 128 -F 16 and Samtools view -b -f 80. Alignments to the reverse strand were selected by Samtools view -b -f 144 and Samtools view -b -f 64 -F 16. Separate bigwig files were then generated using bamCoverage (deepTools 3.3.1) with options -binSize 10–skipNonCoveredRegions–normalizeUsing RPKM–extendReads. In addition, a Homer (4.11)-based workflow for GRO-seq using makeTagDirectory, makeUCSCfile, annotatePeaks.pl and findPeaks all -style groseq -o auto was applied. The workflow is described at http://homer.ucsd.edu/homer/ngs/groseq/groseq.html.

### Reporting summary

Further information on research design is available in the Nature Portfolio Reporting Summary linked to this article.

### Supplementary information

Reporting Summary

### Source data

Source Data Fig. 1 Statistical source data.

Source Data Fig. 2 Statistical source data.

Source Data Fig. 3 Statistical source data.

Source Data Fig. 4 Statistical source data.

Source Data Fig. 5 Statistical source data.

Source Data Fig. 6 Statistical source data.

Source Data Fig. 7 Statistical source data.

Source Data Fig. 8 Statistical source data.

Source Data Extended Data Fig. 1 Statistical source data.

Source Data Extended Data Fig. 2 Statistical source data.

Source Data Extended Data Fig. 3 Statistical source data.

Source Data Extended Data Fig. 4 Statistical source data.

Source Data Extended Data Fig. 5 Statistical source data.

Source Data Extended Data Fig. 6 Statistical source data.

Source Data Extended Data Fig. 7 Statistical source data.

Source Data Extended Data Fig. 8 Statistical source data.

Source Data Extended Data Fig. 9 Statistical source data.

Source Data Extended Data Fig. 10 Statistical source data.

Source Data Figs. 1 and 6 and Extended Data Figs. 2, 6, 8 and 9 Unprocessed western blots.

## Data Availability

The cocrystal structure that supports the findings of this study has been deposited to the Protein Data Bank with the accession number 8P0M and is listed in Extended Data Fig. 1. The ChIP–seq, RNA-seq and TT-seq data with single-agent treatment (48 samples) and the RNA-seq results comparing genetic and pharmacological profiles (36 samples) have all been deposited to SRA under BioProject ID PRJNA991752. The RNA-seq data for the combinations with KRAS^G12C^ inhibitor JDQ443 (210 samples) have been deposited to SRA under BioProject ID PRJNA991764. Source data are provided with this paper. All other data supporting the findings of this study are available from the corresponding authors upon reasonable request.

## Code availability

No custom algorithms were used in this study. Open-source software was used to analyze the data. Details of software versions are specified in the Methods.
