# Author Correction: Direct and selective pharmacological disruption of the YAP–TEAD interface by IAG933 inhibits Hippo-dependent and RAS–MAPK-altered cancers

**DOI:** 10.1038/s43018-024-00797-y

**Published:** 2024-06-17

**Authors:** Emilie A. Chapeau, Laurent Sansregret, Giorgio G. Galli, Patrick Chène, Markus Wartmann, Thanos P. Mourikis, Patricia Jaaks, Sabrina Baltschukat, Ines A. M. Barbosa, Daniel Bauer, Saskia M. Brachmann, Clara Delaunay, Claire Estadieu, Jason E. Faris, Pascal Furet, Stefanie Harlfinger, Andreas Hueber, Eloísa Jiménez Núñez, David P. Kodack, Emeline Mandon, Typhaine Martin, Yannick Mesrouze, Vincent Romanet, Clemens Scheufler, Holger Sellner, Christelle Stamm, Dario Sterker, Luca Tordella, Francesco Hofmann, Nicolas Soldermann, Tobias Schmelzle

**Affiliations:** 1 Novartis BioMedical Research, Basel, Switzerland; 2 Novartis BioMedical Research, Cambridge, MA USA; 3 Present Address: AstraZeneca, Oncology R&D, Cambridge, UK; 4 Present Address: Pierre Fabre Group, R&D Medical Care, Toulouse, France

**Keywords:** Cancer, Cancer therapy

Correction to: *Nature Cancer* 10.1038/s43018-024-00754-9, published online 2 April 2024

In the version of the article initially published, in Fig. 1f, the MSTO-211H graph was originally a duplicate of NCI-H226. This has now been amended, and the correct MSTO-211H graph can be found in the HTML and PDF versions of the article.

