# Supplementary material for: Direct and selective pharmacological disruption of the YAP–TEAD interface by IAG933 inhibits Hippo-dependent and RAS–MAPK-altered cancers
Source: Nat Cancer. 2024 Apr 2;5(7):1102–20. doi: 10.1038/s43018-024-00754-9 (PMC11286534; doi:10.1038/s43018-024-00754-9)
Supplement: Supplementary file 1 — Reporting Summary [file 43018_2024_754_MOESM1_ESM.pdf]

nature portfolio

Corresponding author(s): Emilie Chapeau ; Tobias Schmelzle; NATCANCER-A09662B

Last updated by author(s): Feb 01, 2024

Reporting Summary

Nature Portfolio wishes to improve the reproducibility of the work that we publish. This form provides structure for consistency and transparency in reporting. For further information on Nature Portfolio policies, see our Editorial Policies and the Editorial Policy Checklist.

Statistics

For all statistical analyses, confirm that the following items are present in the figure legend, table legend, main text, or Methods section.

n/a	Confirmed	
☐	☒	The exact sample size (*n*) for each experimental group/condition, given as a discrete number and unit of measurement
☐	☒	A statement on whether measurements were taken from distinct samples or whether the same sample was measured repeatedly
☐	☒	The statistical test(s) used AND whether they are one- or two-sided<br>*Only common tests should be described solely by name; describe more complex techniques in the Methods section.*
☐	☒	A description of all covariates tested
☐	☒	A description of any assumptions or corrections, such as tests of normality and adjustment for multiple comparisons
☐	☒	A full description of the statistical parameters including central tendency (e.g. means) or other basic estimates (e.g. regression coefficient) AND variation (e.g. standard deviation) or associated estimates of uncertainty (e.g. confidence intervals)
☐	☒	For null hypothesis testing, the test statistic (e.g. *F*, *t*, *r*) with confidence intervals, effect sizes, degrees of freedom and *P* value noted<br>*Give P values as exact values whenever suitable.*
☒	☐	For Bayesian analysis, information on the choice of priors and Markov chain Monte Carlo settings
☒	☐	For hierarchical and complex designs, identification of the appropriate level for tests and full reporting of outcomes
☐	☒	Estimates of effect sizes (e.g. Cohen's *d*, Pearson's *r*), indicating how they were calculated

Our web collection on statistics for biologists contains articles on many of the points above.

Software and code

Policy information about availability of computer code

Data collection

X-ray diffraction data were collected with the Swiss Light Source (SLS, beamline X10SA) using a Pilatus pixel detector.
Biochemical experiments used Biacore T200 (Cytivia, Marlborough, MA) and Genios Pro reader (Tecan, Switzerland).
Cellular assays used M200 multi-purpose plate reader (TECAN), Cellomics ArrayScan VTI high content imager (ThermoFisher), and Incucyte live cell imaging technology.
IHC acquisition was done with Scanscope XT slide scanner (Aperio).
qPCR data was obtained by QuantStudio 6 Flex device (Applied Biosystems).
Pharmacokinetics results were obtained with the Triple quadrupole mass spectrometer Xevo TQ-xs™ (Waters Corporation, Milford, MA, USA).
In vivo imaging was performed with IVIS Spectrum (Perkin Elmer) and CentroXS LB960 Luminometer (Berthold Technologies).
Immunoblotting used Fusion-FX7 edge camera (Vilber Lourmat).
Genomics sequencing were obtained with HiSeq 4000 device (Illumina), HiSeq2500 device (Illumina), Novaseq (Illumina).

Data analysis	X-ray analyses: autoPROC,2 STARANISO, PHASER3, COOT4 and BUSTER (version 2.11.8) and PyMol.<br>Biochemical analyses : Biacore T200 evaluation software (Cytivia, Marlborough, MA)<br>IHC : HALO Area Quantification algorithm and CytoNuclear algorithm (Indica Labs)<br>Animal imaging : IVIS Spectrum sotware (Perkin Elmer)<br>Pharmacokinetics : Phoenix® Certara, US<br>Genomics : RSeQC (v3.0.0), PISCES v.2018.04.1, DESeq2, R Bioconductor - clusterProfiler package (v 2.10.0), STAR (2.5.2a) aligner, Samtools (1.12), deepTools 3.3.1, Homer (4.11)<br>GraphPad Prism (GraphPad Software)<br>Excel (Microsoft)

For manuscripts utilizing custom algorithms or software that are central to the research but not yet described in published literature, software must be made available to editors and reviewers. We strongly encourage code deposition in a community repository (e.g. GitHub). See the Nature Portfolio guidelines for submitting code & software for further information.

Data

Policy information about availability of data

All manuscripts must include a data availability statement. This statement should provide the following information, where applicable:

- Accession codes, unique identifiers, or web links for publicly available datasets
- A description of any restrictions on data availability
- For clinical datasets or third party data, please ensure that the statement adheres to our policy

The co-crystal structure that support the findings of this study have been deposited to the Protein Data Bank with the accession number 8P0M and are listed in Extended Data Fig. 1. The ChIP-seq, RNA-seq and TT-seq data with single agent treatment (48 samples), and the RNA-seq results comparing genetic and pharmacological profiles (36 samples), have all been deposited to SRA with BioProject ID: PRJNA991752. The RNA-seq for the combinations with KRASG12C inhibitor JDQ443 (210 samples) have been deposited to SRA with BioProject ID: PRJNA991764. Source data are provided with this paper. All other data supporting the findings of this study are available from the corresponding authors upon reasonable request.

Research involving human participants, their data, or biological material

Policy information about studies with human participants or human data. See also policy information about sex, gender (identity/presentation), and sexual orientation and race, ethnicity and racism.

Reporting on sex and gender	not applicable
Reporting on race, ethnicity, or other socially relevant groupings	not applicable
Population characteristics	not applicable
Recruitment	not applicable
Ethics oversight	not applicable

Note that full information on the approval of the study protocol must also be provided in the manuscript.

Field-specific reporting

Please select the one below that is the best fit for your research. If you are not sure, read the appropriate sections before making your selection.

☒ Life sciences ☐ Behavioural & social sciences ☐ Ecological, evolutionary & environmental sciences

For a reference copy of the document with all sections, see nature.com/documents/nr-reporting-summary-flat.pdf

Life sciences study design

All studies must disclose on these points even when the disclosure is negative.

Sample size	Sample sizes were determined based on the frequently used number of experimental replicates in the available literature rather than on sample size calculations. The sample size was also selected in order to enable statistical analysis. The sample size for in vivo experiments was sufficient to evaluate statistical significance. No statistical method was used to predetermine sample size.
Data exclusions	No data was excluded from analyses. Sex of tumor models was not considered in the study design
Replication	Most experiments were performed at least twice with similar results.
Randomization	For mouse or rat in vivo experiments, when tumors reached a volume of approximately 150-250 mm3, animals were randomized based on tumor size and assigned to different experimental groups.

Blinding

Investigators were blinded to group allocation during genomics data processing. For other experiments, no blinding was applied. Blinding is impossible in biological and biochemical experiments due to the experimenter implementing treatments, the complexity of experiments, and ethical considerations.

Reporting for specific materials, systems and methods

We require information from authors about some types of materials, experimental systems and methods used in many studies. Here, indicate whether each material, system or method listed is relevant to your study. If you are not sure if a list item applies to your research, read the appropriate section before selecting a response.

Materials & experimental systems

n/a	Involved in the study
☐	☒ Antibodies
☐	☒ Eukaryotic cell lines
☒	☐ Palaeontology and archaeology
☐	☒ Animals and other organisms
☒	☐ Clinical data
☒	☐ Dual use research of concern
☒	☐ Plants

Methods

n/a	Involved in the study
☐	☒ ChIP-seq
☒	☐ Flow cytometry
☒	☐ MRI-based neuroimaging

Antibodies

Antibodies used

anti-YAP antibody (EP1674Y, Abcam)
anti-TAZ antibody (Cell Signaling Technology #4883)
anti-TEF-1 (TEAD1) antibody (Becton Dickinson)
AlexaFluor 488-labeled goat anti-rabbit antibody (Invitrogen)
Alexa Fluor 568-labeled goat anti-mouse antibody (Invitrogen)
anti-Ki67 clone SP6 (Neomarkers, #RM9106)
cleaved Caspase3 (CST, #9661)
cleaved PARP (CST, #9541)
Pan-TEAD D3F7L (Cell Signaling Technology #13295)
anti-YAP (D8H1X) XP (Cell Signaling Technology #14074S);
anti-TEF1 (BD #610922);
anti-Vinculin (Sigma #V9131);
anti-V5-Tag (Cell Signaling Technology #80076);
anti-KRAS (3B10-2F2) (Novus #H00003845-M01);
anti-RSK1-RSK2-RSK3 (32D7) (Cell Signaling Technology #9355);
anti-phospho-MAPK (Thr202/Tyr204) (Cell Signaling Technology #9101);
anti-phospho-RSK3 (T356/S360) (Cell Signaling Technology #9348);
anti- MAPK (Cell Signaling Technology #9102);
anti-MCL1 (ENZO #ADI-AAP-240-F);
anti-Bcl-xL (Cell Signaling Technology #2764);
anti-BMF (Cell Signaling Technology #50542);
anti-cleaved PARP (Cell Signaling Technology #5625);
anti-BIM (Cell Signaling Technology #2933);
anti-GAPDH (Cell Signaling Technology #8884);
anti-Actin Clone C4 (Millipore #MAB1501);
anti-β-Tubulin (Sigma-Merck #T4026).
TEAD4 (Abcam #ab58310),
H3K27ac (Cell Signaling Technology #8173),
H3K4me1 (Cell Signaling Technology #5326)
Secondary antibodies used were anti-mouse or anti-rabbit (Cell Signaling Technology #7074, #7076), Veriblot-HRP (Abcam #Ab131366), anti-rabbit-HRP (Dako #P0448), or anti-mouse-HRP (Amersham GE Healthcare #NA931).

Validation

Antibodies were not orthogonally validated in-house. Antibodies with as many trusted citations as possible were used. All antibodies functioned as expected for their respective assays and included positive or negative controls.

Eukaryotic cell lines

Policy information about cell lines and Sex and Gender in Research

Cell line source(s)

All human cancer cell lines are part of the Broad-Novartis Cancer Cell Line Encyclopedia (https://sites.broadinstitute.org/ccle). Non-human cell lines were purchased at ATCC® : MDCK (ATCC CCL-34), NIH-3T3 (ATCC CRL-1658), CT-26 (ATCC CRL-2638), and RAT-1 (ATCC CRL-2210).

Authentication

All cell lines are tested for authentication regularly by SNP profiling, and were used less than 15 passages to avoid genetic drifting.

Mycoplasma contamination

Cell lines are regularly confirmed to be negative for mycoplasma contamination by PCR testing. For in vivo implantation, cell lines are confirmed Mycoplasma-free and pathogen-free (with IMPACT-8; IDEXX BioAnalytics.

Commonly misidentified lines
(See ICLAC register)

no commonly misidentified cell line was used

Animals and other research organisms

Policy information about studies involving animals; ARRIVE guidelines recommended for reporting animal research, and Sex and Gender in Research

Laboratory animals	Female nude rats, Crl:NIH-FOXn1rnu-Homozygous and Female nude mice, Crl:NU(NCr)-Foxn1nu-Homozygous were purchased from CRL Germany. Female SCID mice, C.B-Igh-1b/IcrTac-Prkdcscid were purchased from Taconic Europe. They were between 8-20 week-old approximately at the time of the experiment. The animals were housed in a 12h light/dark cycle facility and had access to sterilized food and water ad libitum. Animals were allowed to accommodate at least for 7 days before handling. The maximal tumor size/burden permitted is 1500mm3 and was not exceeded.All animal studies were conducted in accordance with ethics and procedures covered by permit BS-1763 and BS-1767, respectively whether the model was induced ectopically or orthotopically, issued by the Kantonales Veterinäramt Basel-Stadt and in strict adherence to guidelines of the Eidgenössisches Tierschutzgesetz and the Eidgenössische Tierschutzverordnung, Switzerland.
Wild animals	no wild animals used in these studies
Reporting on sex	no gender-based analysis was performed
Field-collected samples	This study did not use field collected samples
Ethics oversight	All animal studies were conducted in accordance with ethics and procedures covered by permit BS-1763 and BS-1767, issued by the Kantonales Veterinäramt Basel-Stadt and in strict adherence to guidelines of the Eidgenössisches Tierschutzgesetz and the Eidgenössische Tierschutzverordnung, Switzerland.

Note that full information on the approval of the study protocol must also be provided in the manuscript.

Plants

Seed stocks	not applicable
Novel plant genotypes	not applicable
Authentication	not applicable

ChIP-seq

Data deposition

☒ Confirm that both raw and final processed data have been deposited in a public database such as GEO.

☒ Confirm that you have deposited or provided access to graph files (e.g. BED files) for the called peaks.

Data access links<br>*May remain private before publication.*	Deposited to SRA with BioProject ID: PRJNA991752
Files in database submission	FastQ
Genome browser session<br>(e.g. UCSC)	N/A. Data from repository can be fully downloaded from repository and processed according to standard ENCODE pipeline

Methodology

Replicates	two independent replicates were performed
Sequencing depth	In average 60 millions reads per sample, and >25 milions uniquely mapped reads.
Antibodies	YAP (Abcam #ab52771), TEAD4 (Abcam #ab58310), H3K27ac (Cell Signaling Technology #8173), and H3K4me1 (Cell Signaling Technology #5326).
Peak calling parameters	Peak calling has been performed using MACS2 version 2.2.7.1. with default parameters
Data quality	All samples passed ENCODE QC pipeline using MultiQC version 1.6
Software	Standard software packages were employed.
