# Supplementary material for: Direct and selective pharmacological disruption of the YAP–TEAD interface by IAG933 inhibits Hippo-dependent and RAS–MAPK-altered cancers
Source: Nat Cancer. 2024 Apr 2;5(7):1102–20. doi: 10.1038/s43018-024-00754-9 (PMC11286534; doi:10.1038/s43018-024-00754-9)

Fig 1b

MSTO-211H coIP with panTEAD

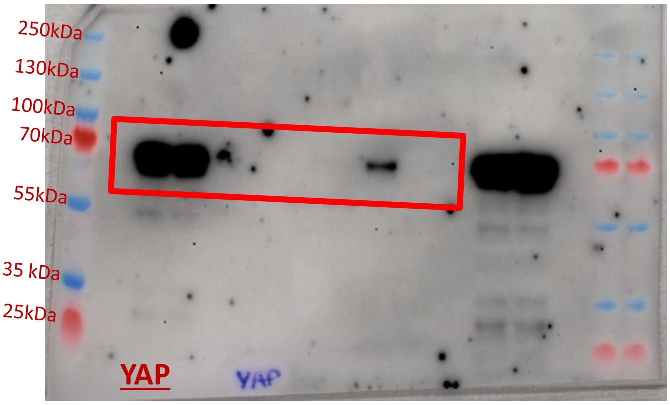

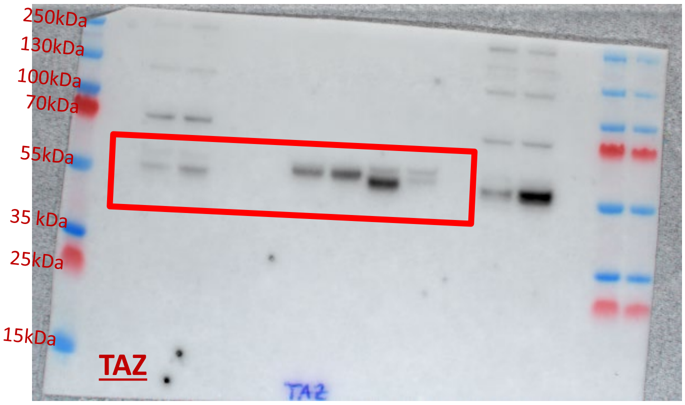

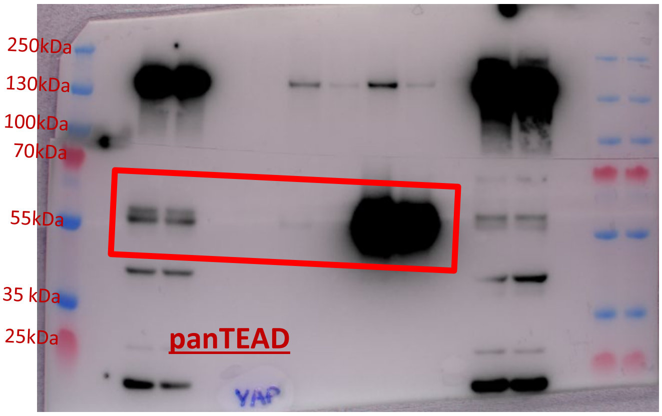

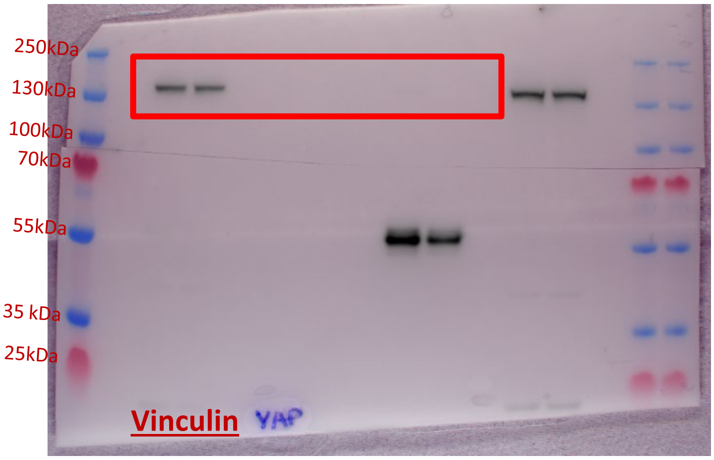

Fig 1b

NCI-H2052

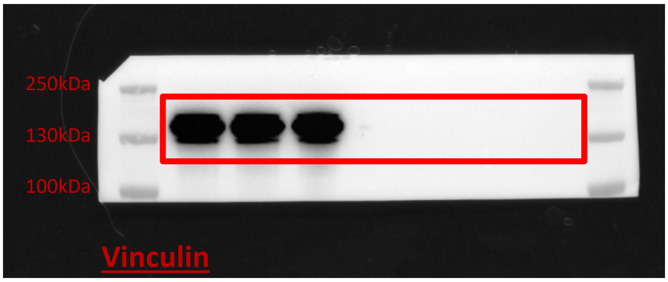

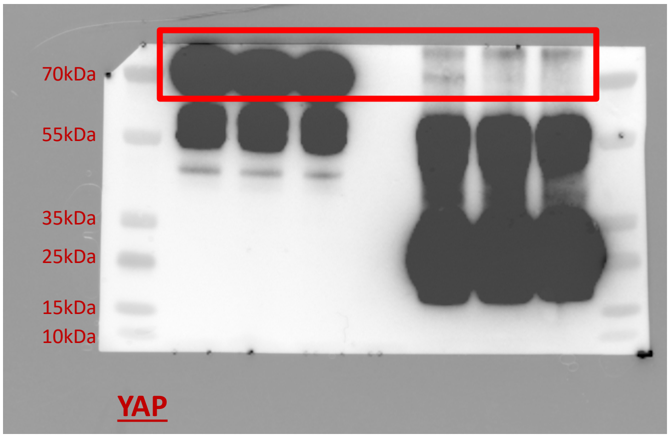

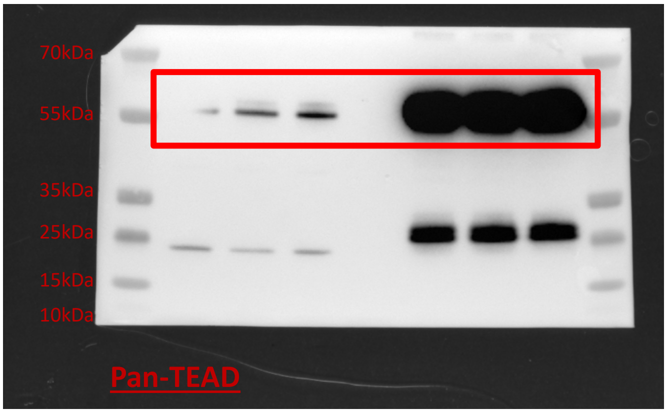

Fig 6f

HCC-1171

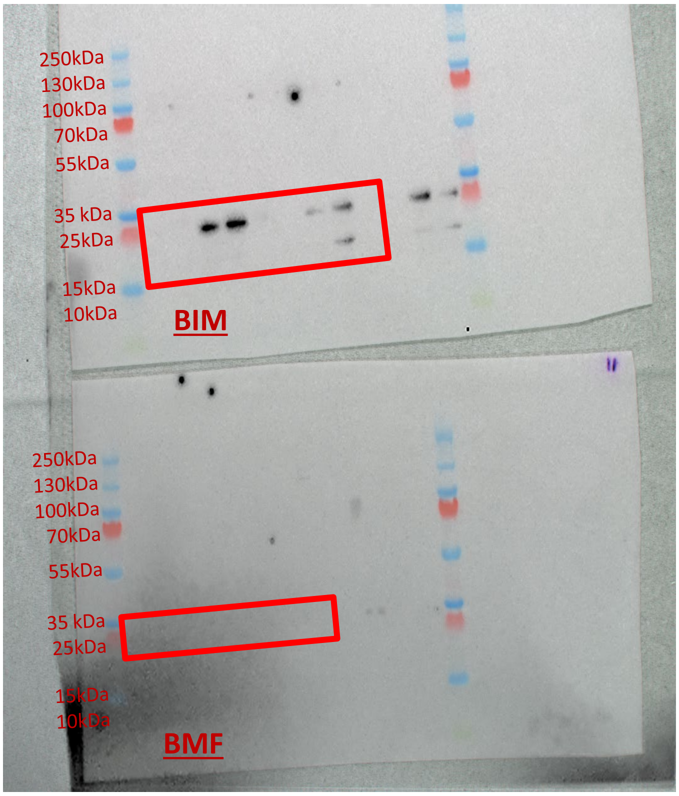

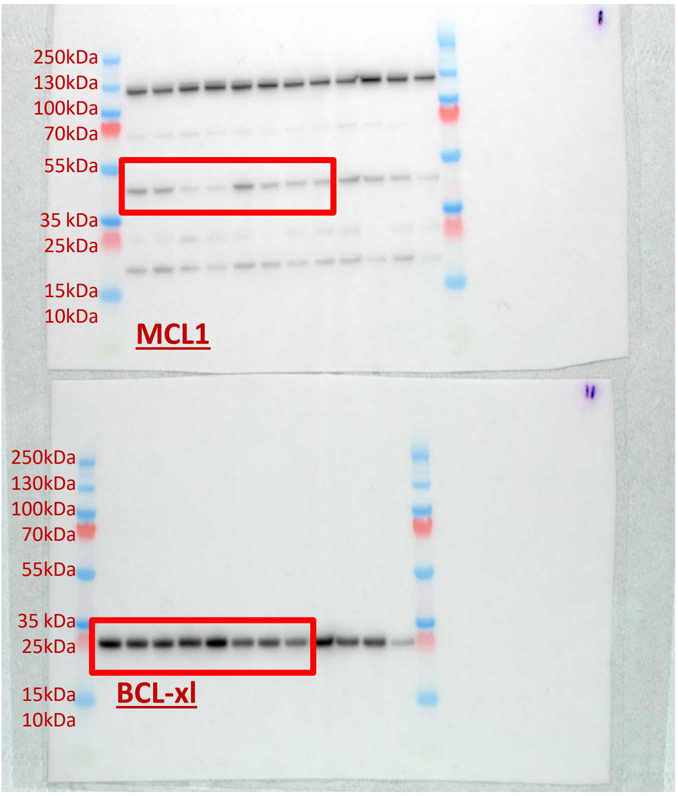

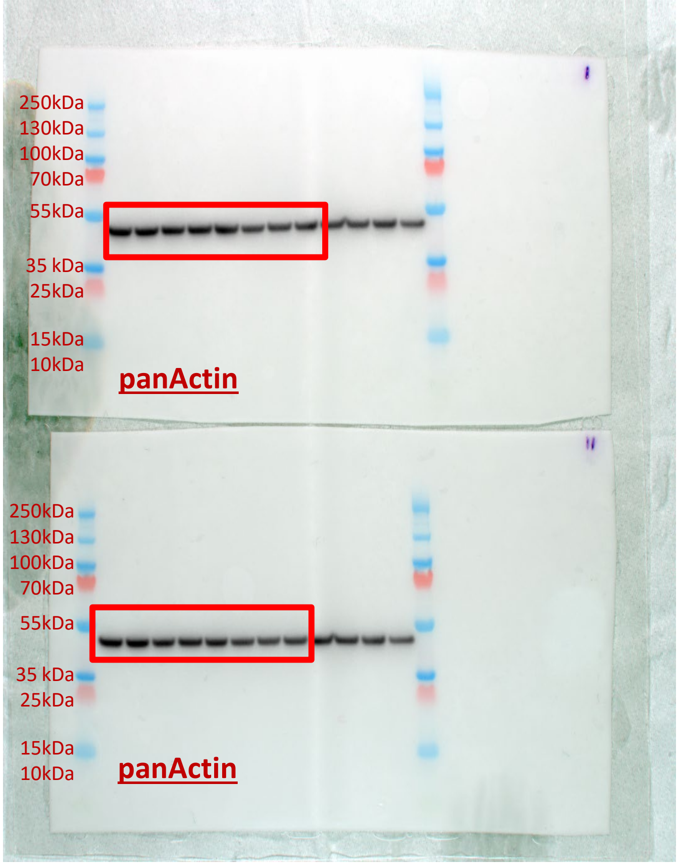

Fig 6g

HCC-1171

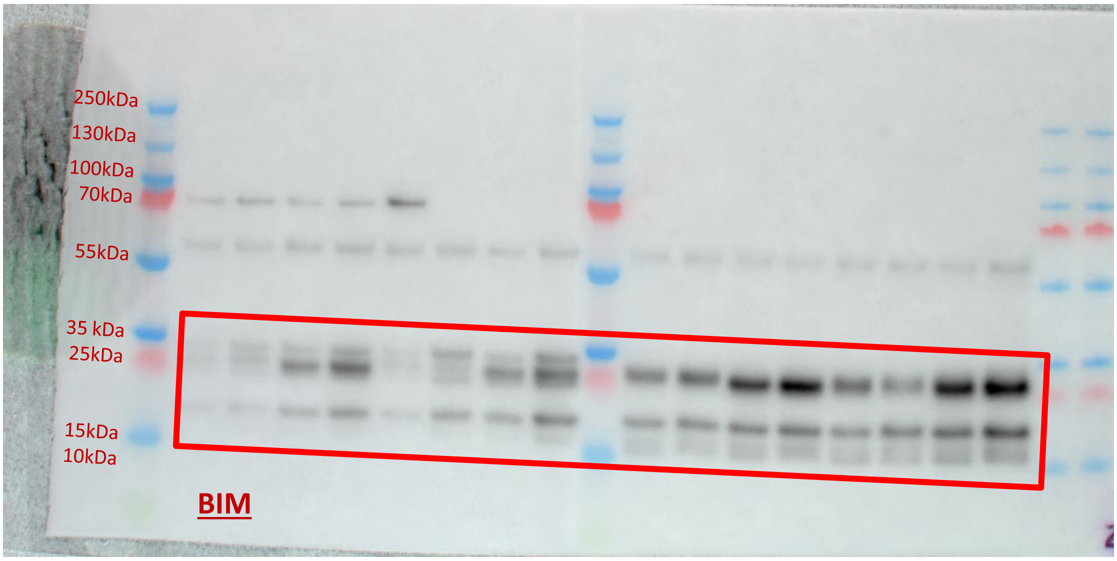

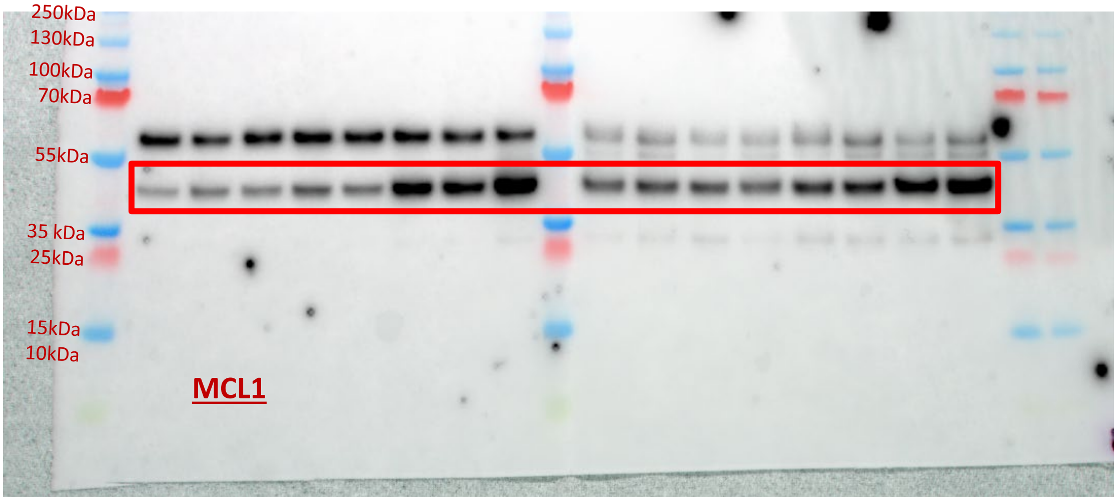

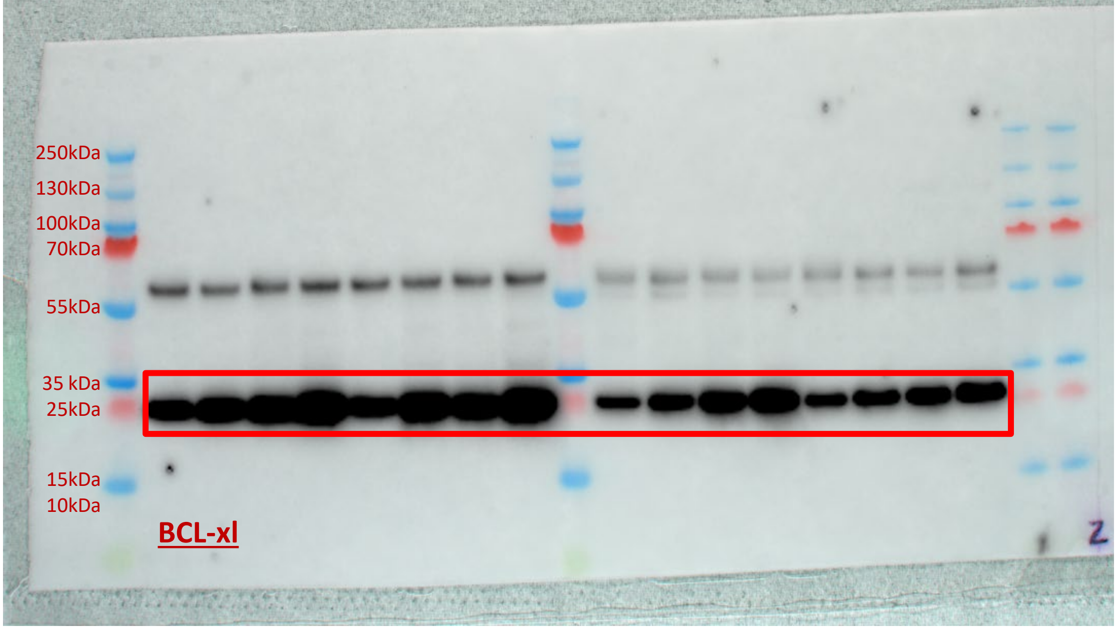

Fig S2b

MSTO-211H coIP with panTEAD and YTP-75

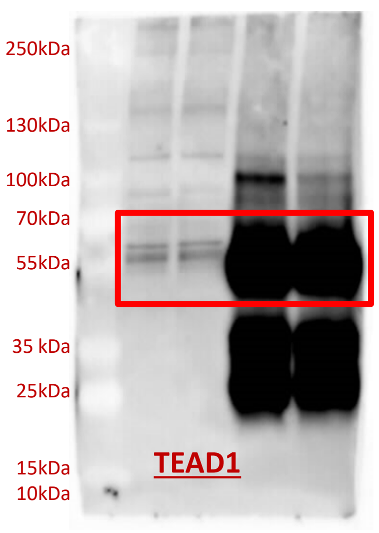

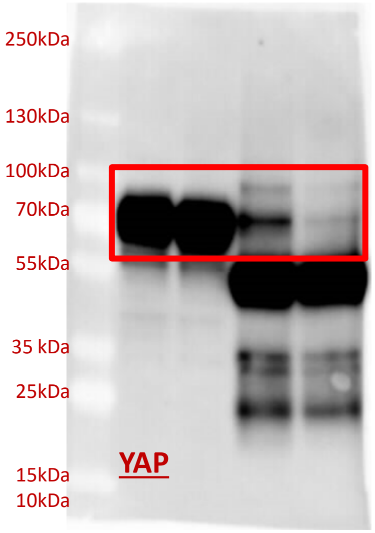

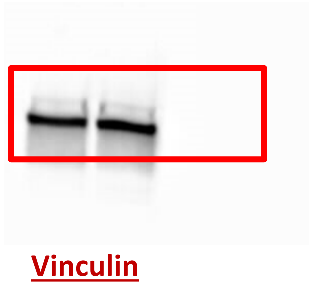

MSTO-211H

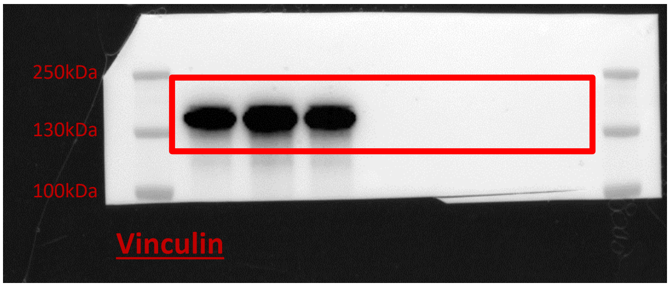

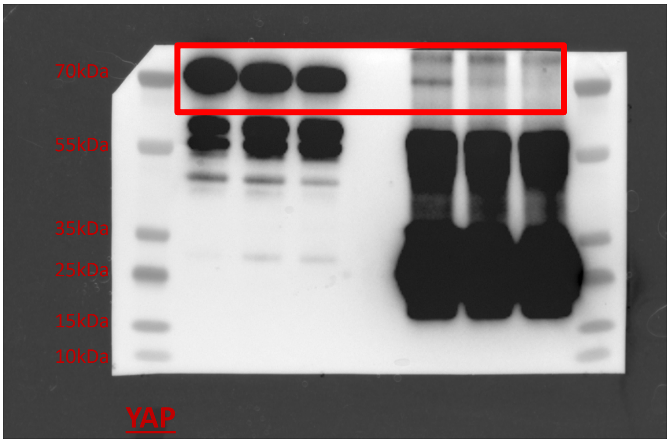

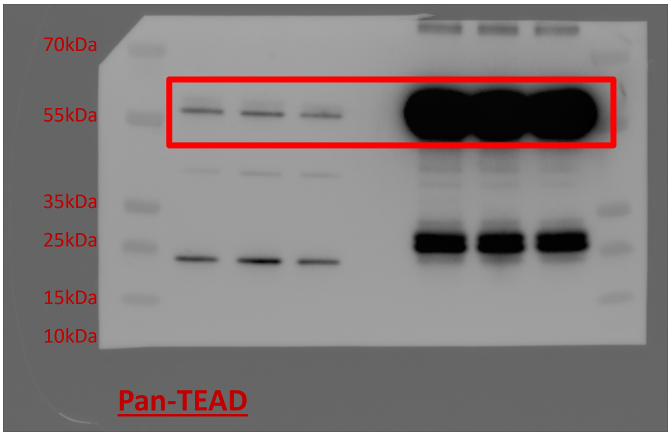

Fig S6a

MSTO-211H xenograft tumors – single dose IAG933 – 30mg/kg p.o.

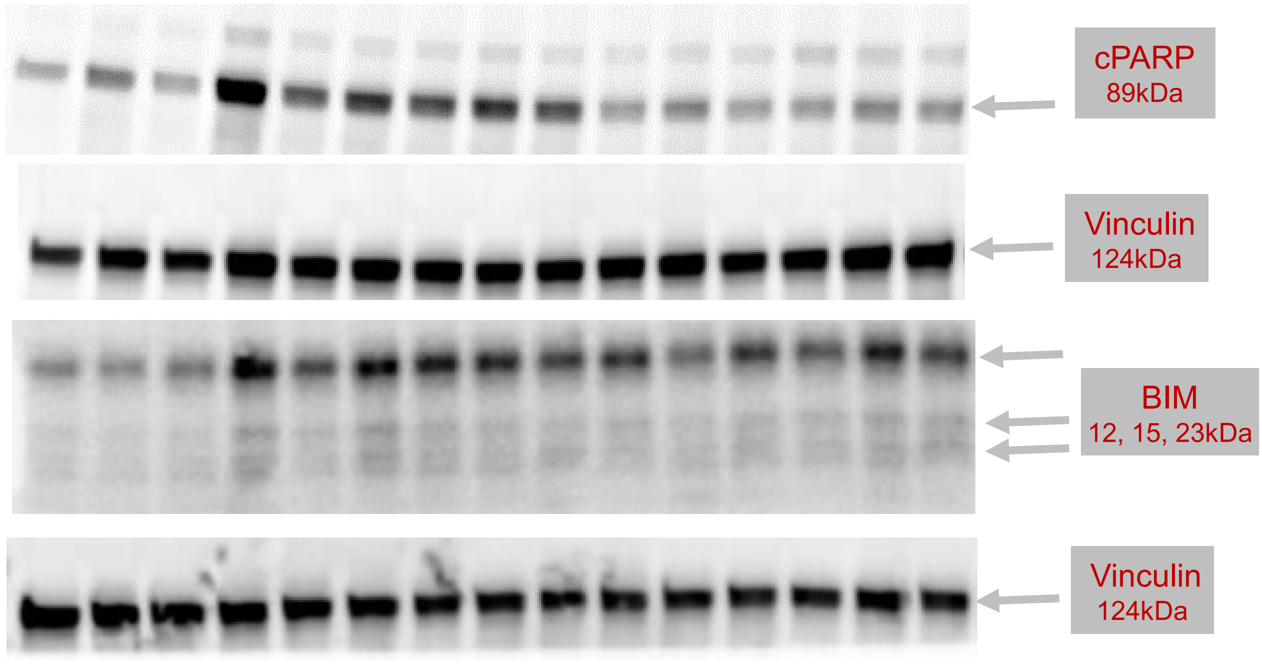

MSTO-211H xenograft tumors – single dose IAG933 – 240mg/kg p.o.

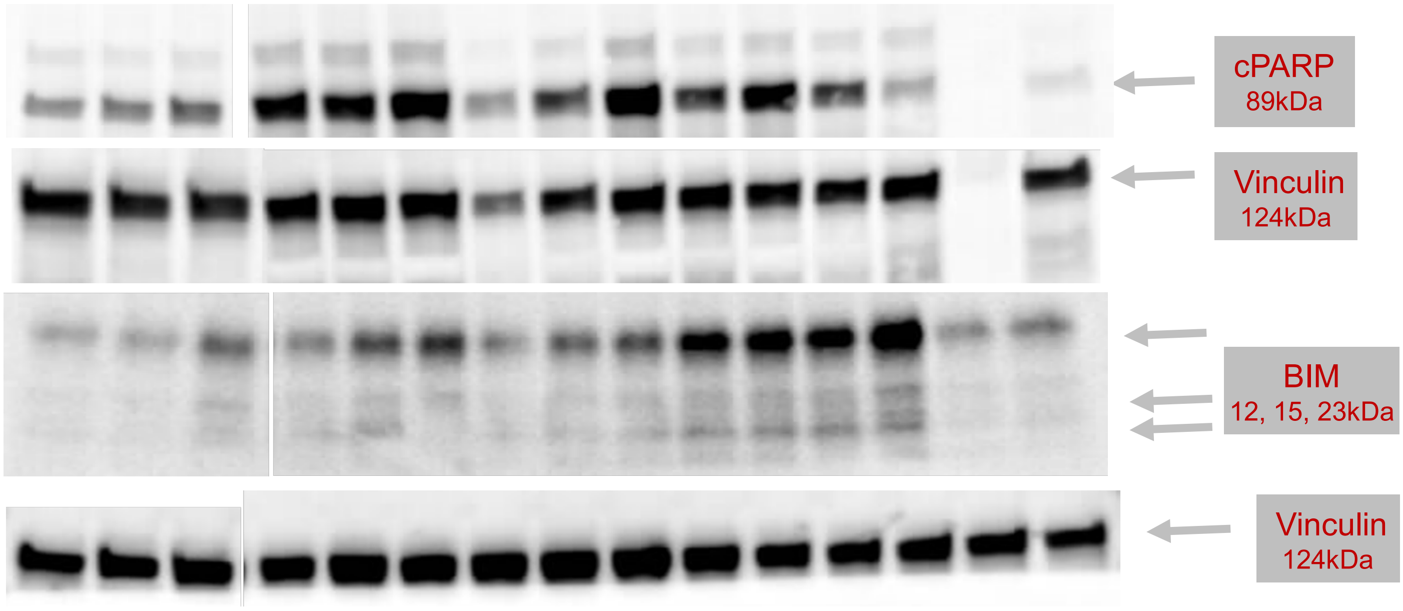

Fig S8a

NIH-3T3-pXP1510 YAP-MAML2, TAZ-CAMTA1 & YAP WT

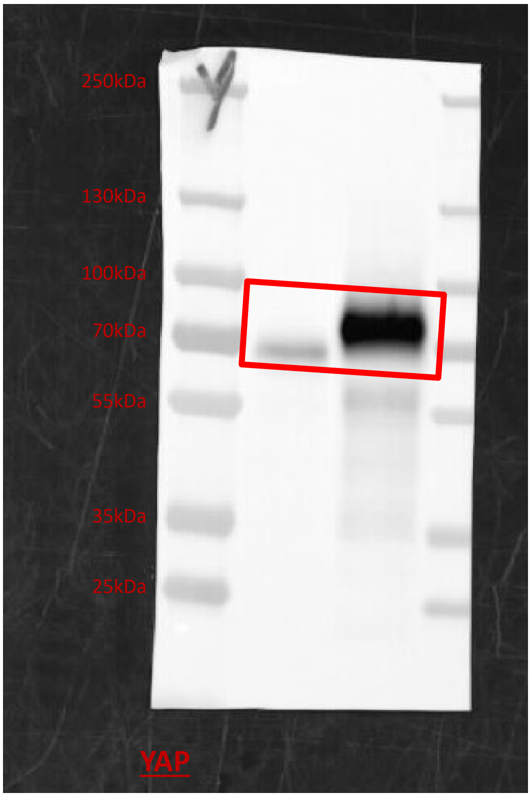

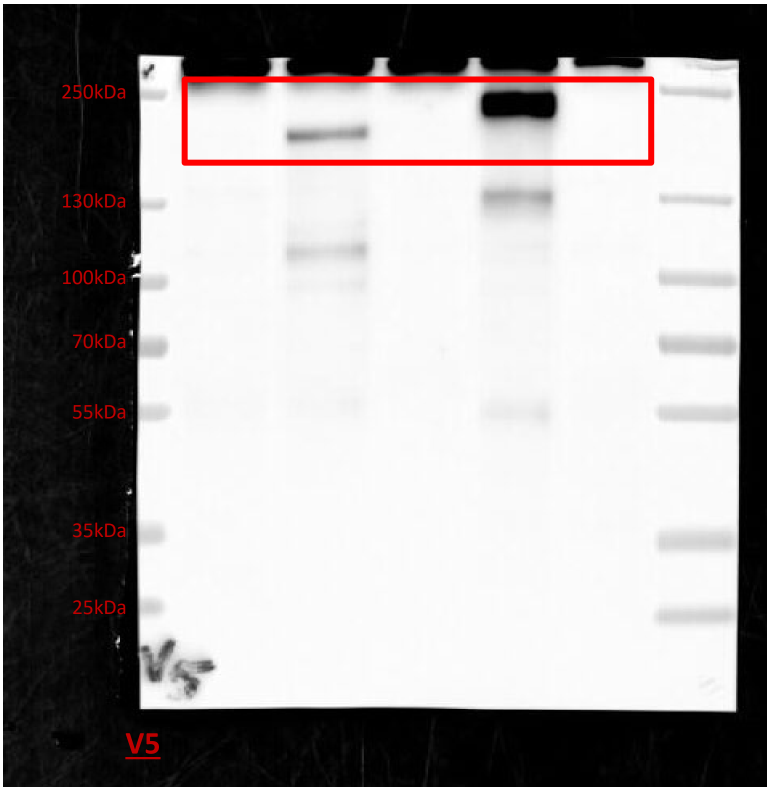

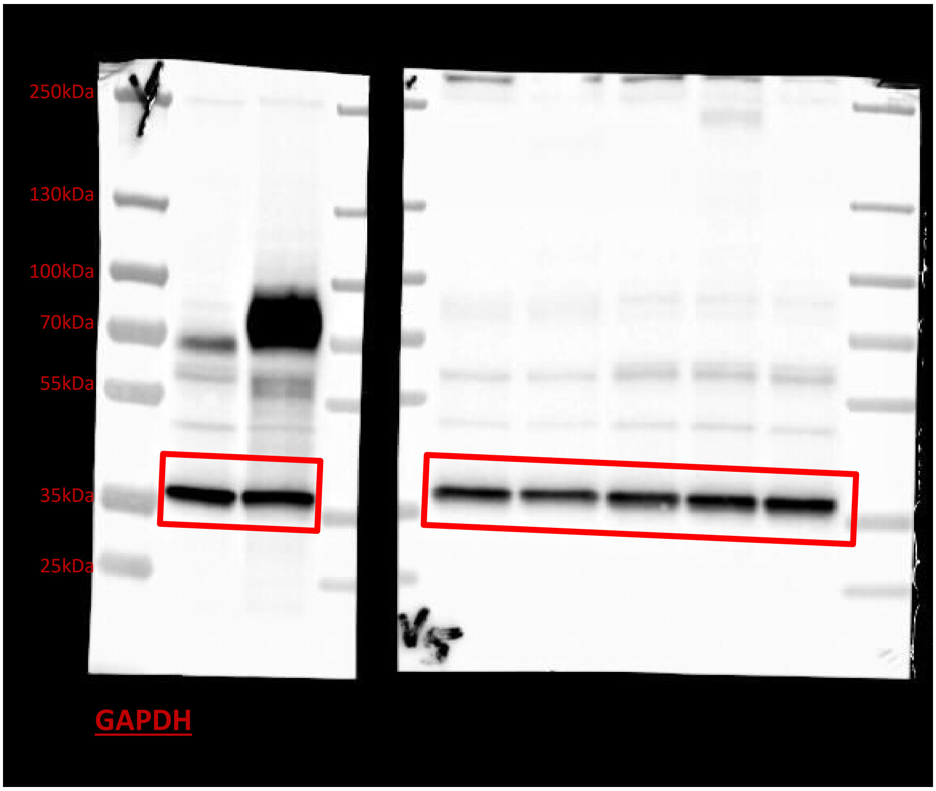

Fig S9d

NCI-H1792

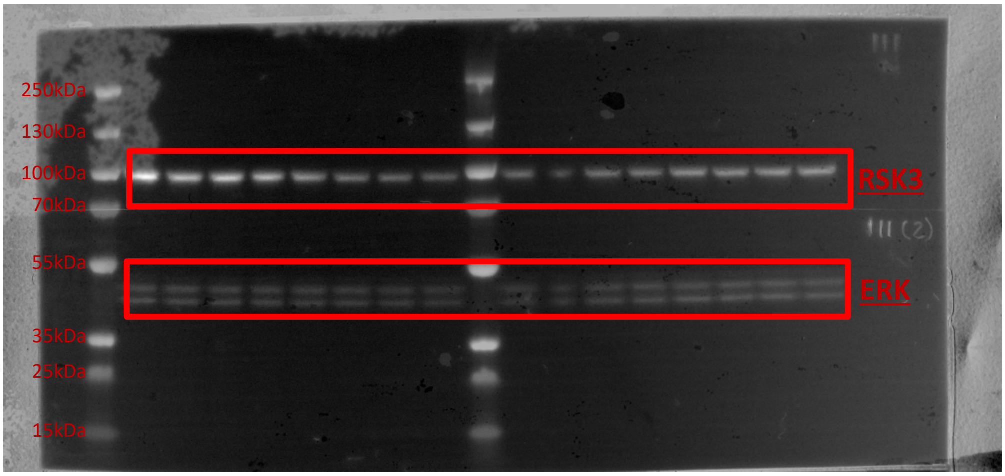

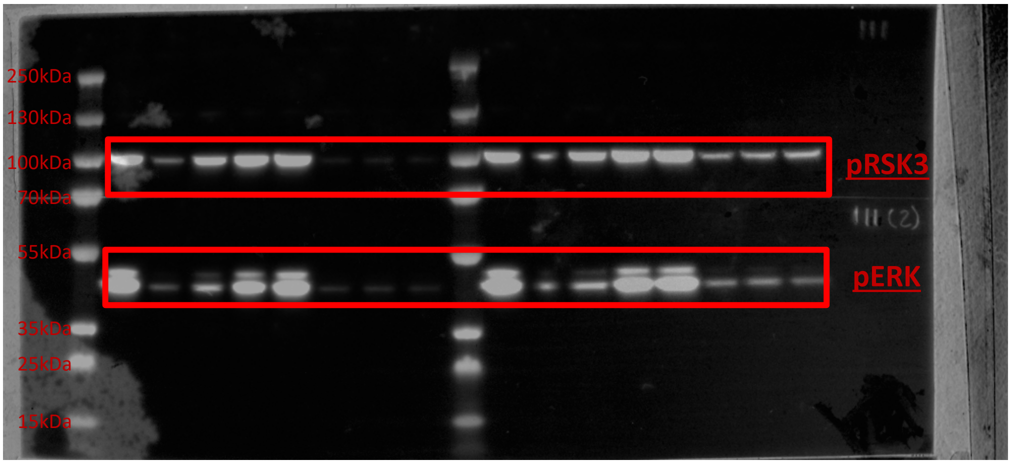

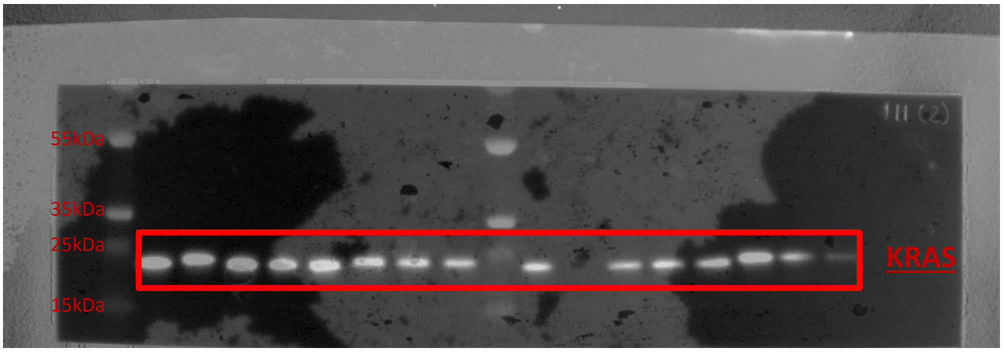

Fig S9d

NCI-H1373

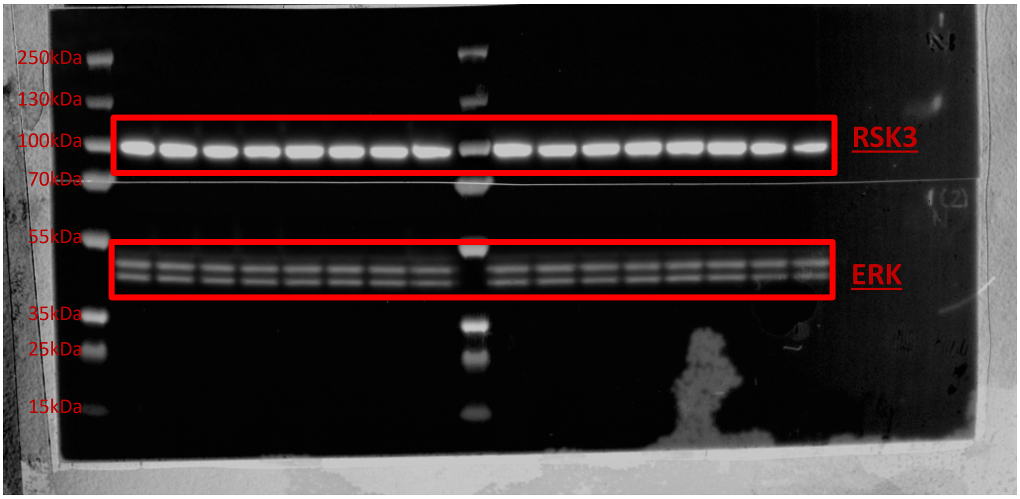

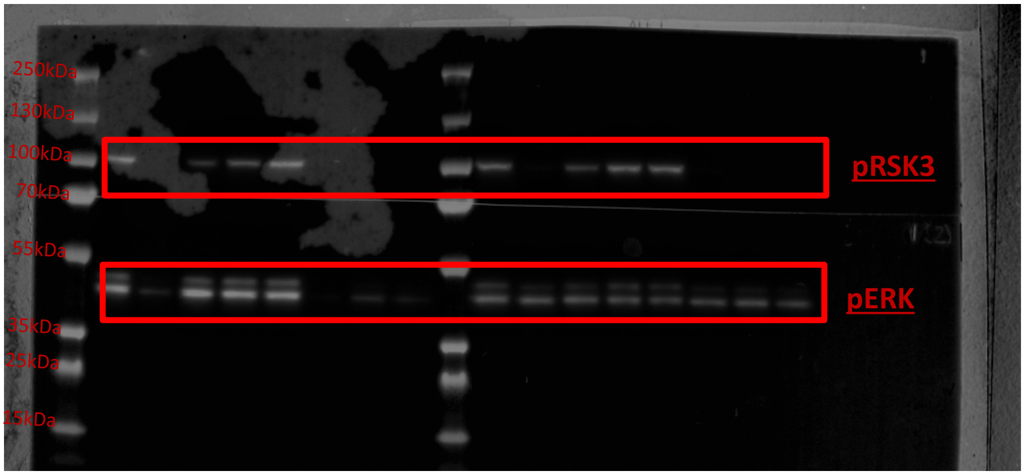

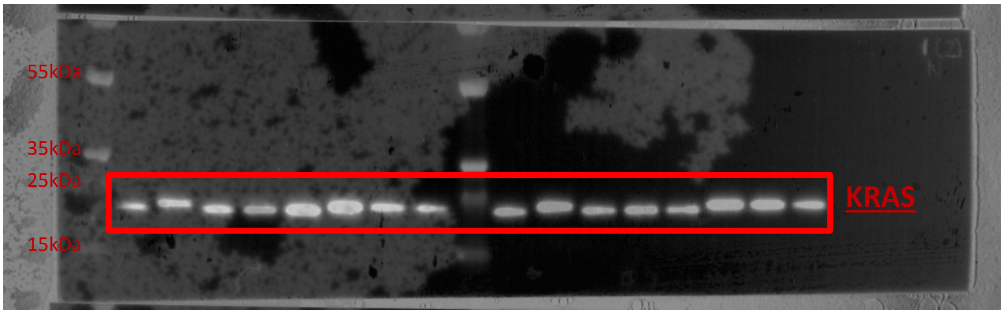

Fig S9d

Calu1

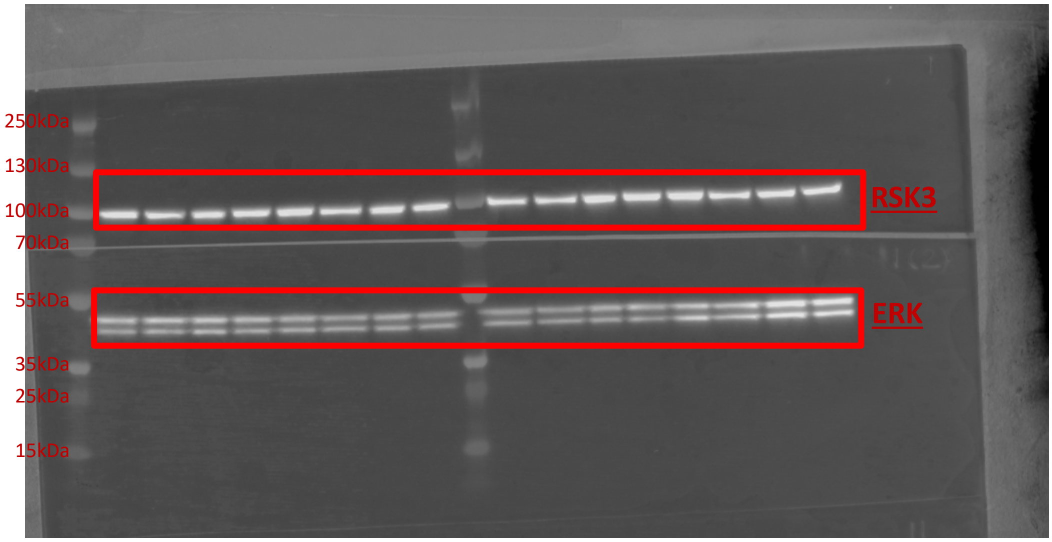

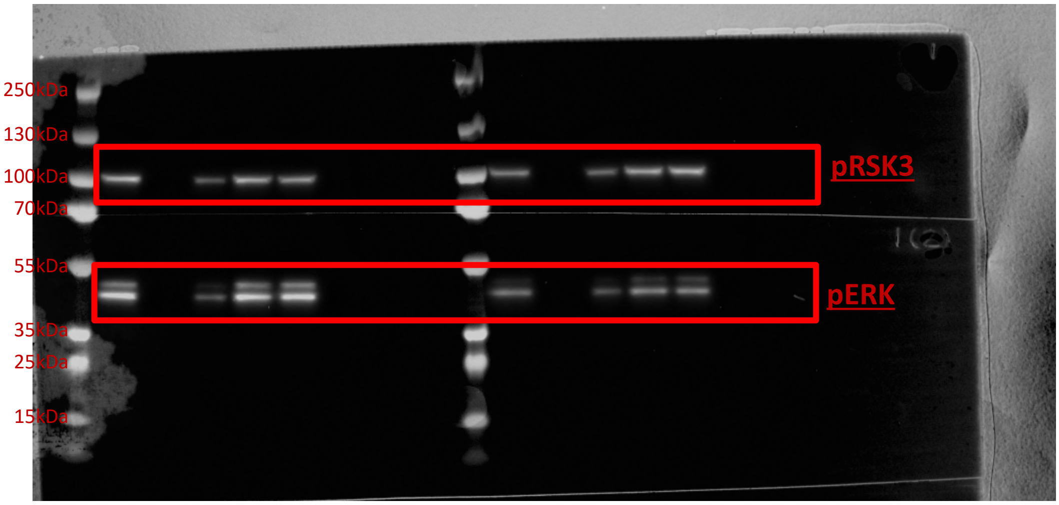

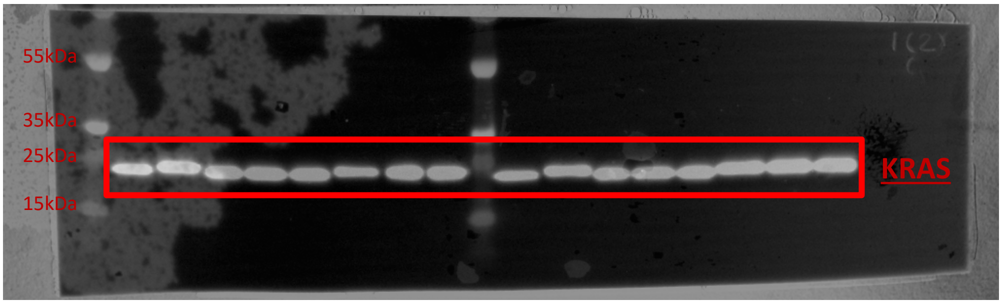

Fig S9e

HCC44

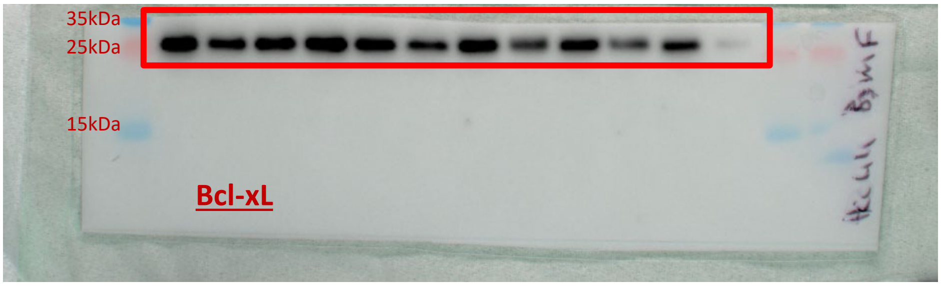

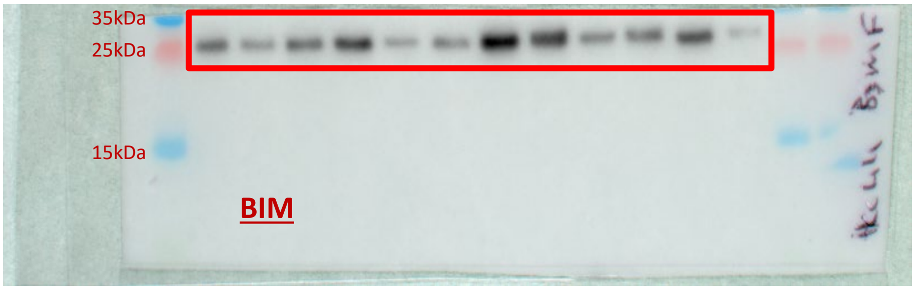

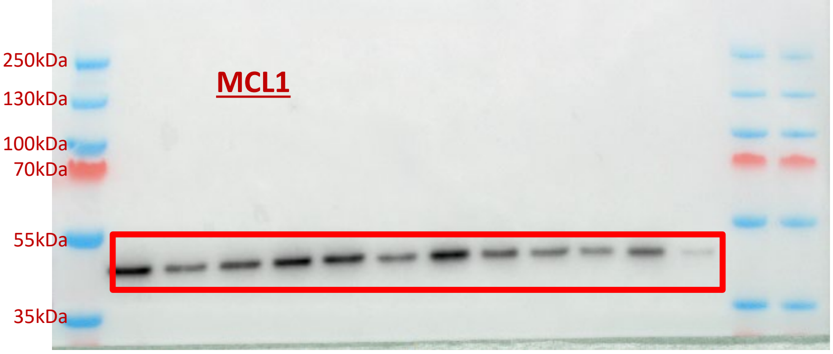

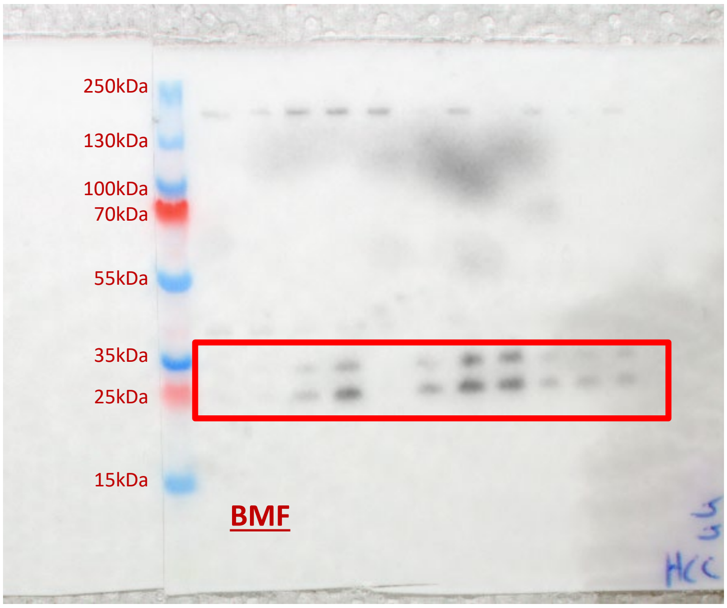

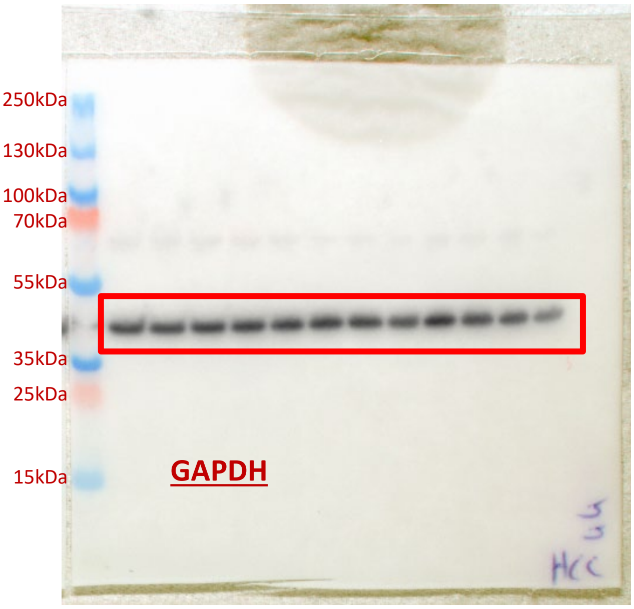

Fig S9e

NCI-H1373

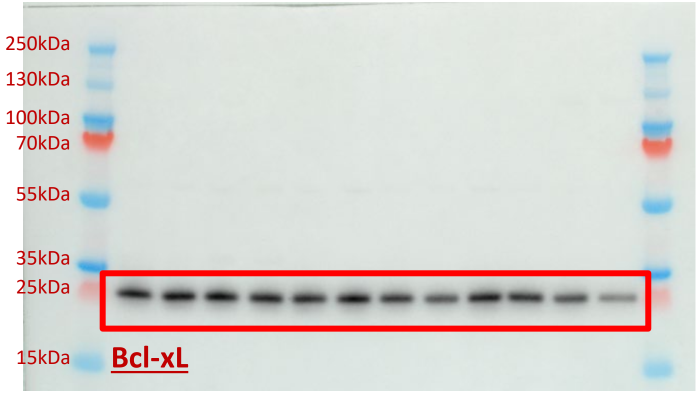

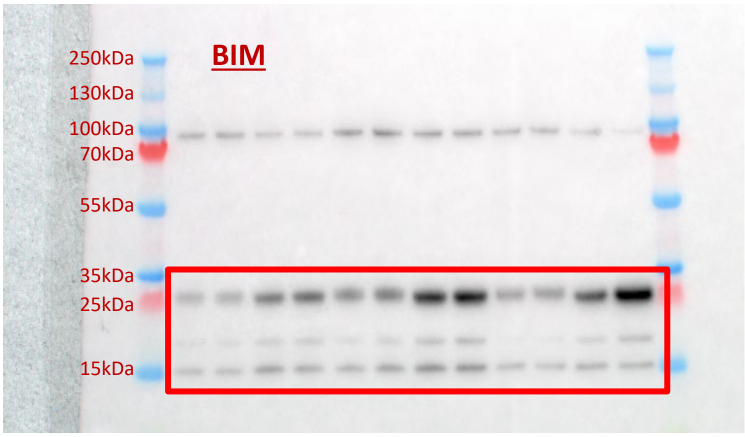

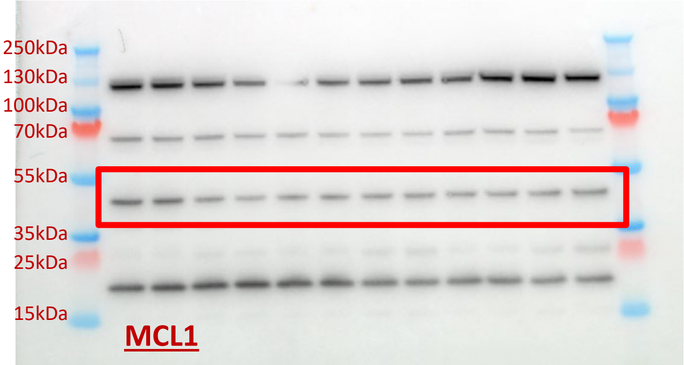

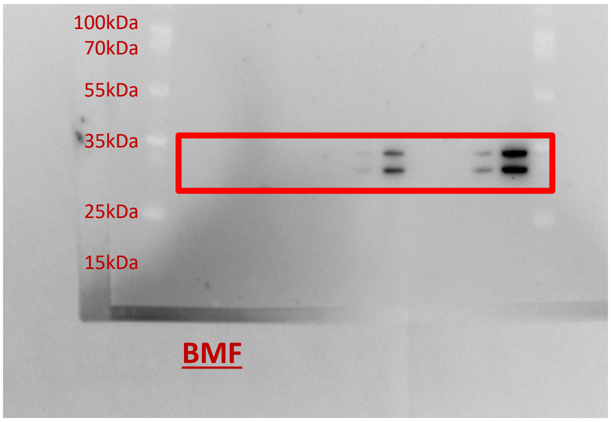

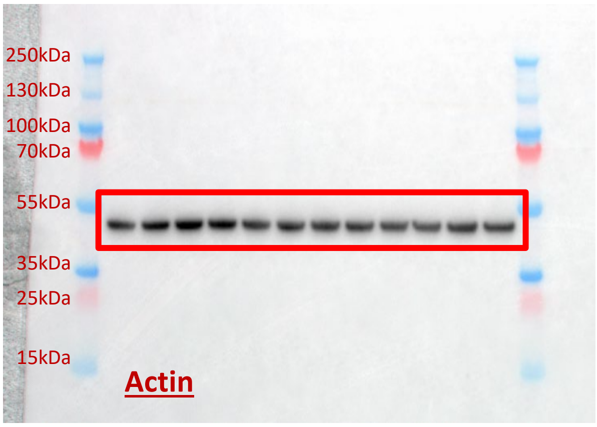

Fig S9e

Calu1

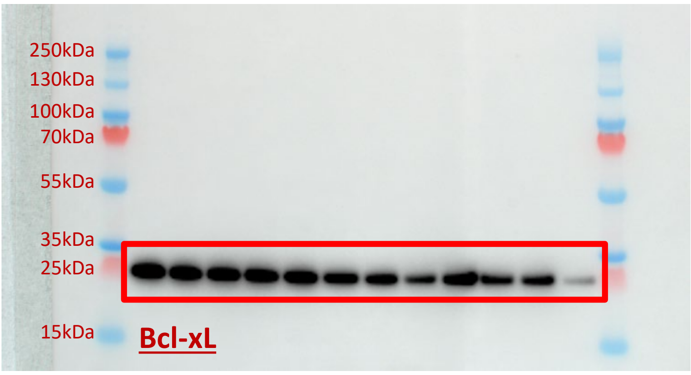

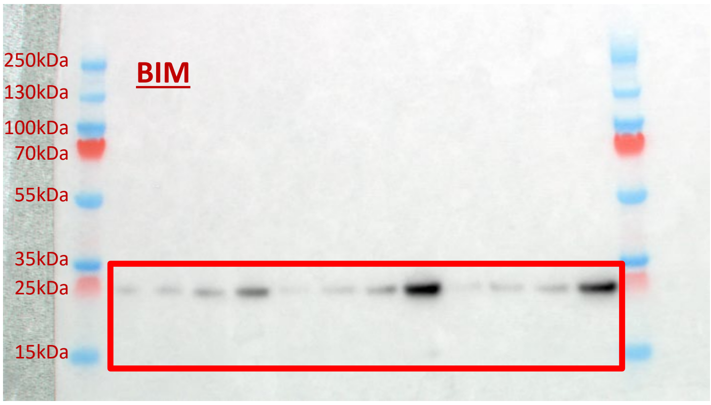

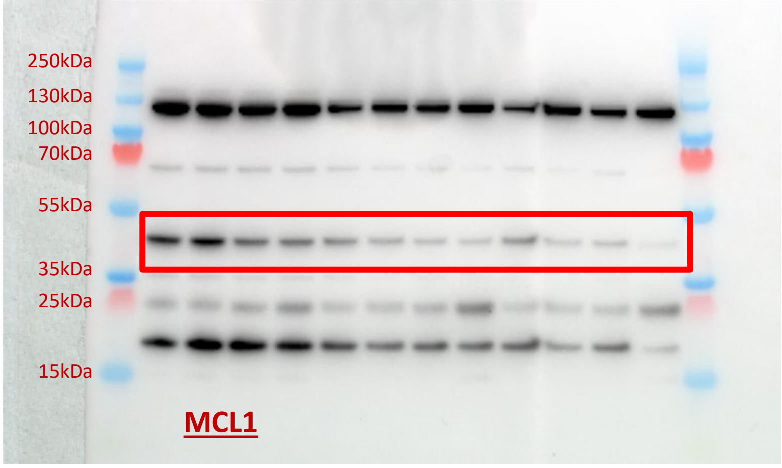

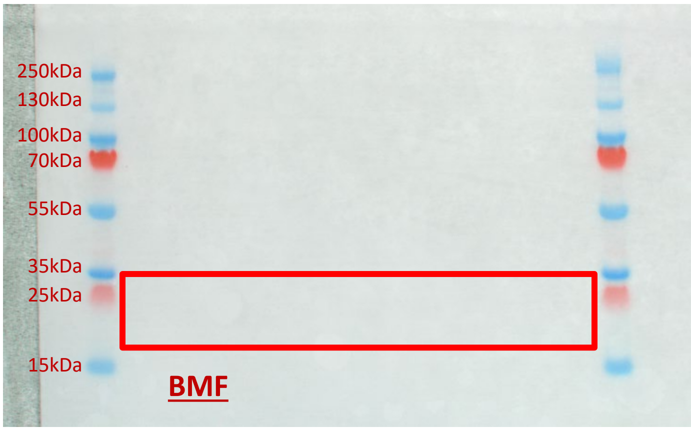

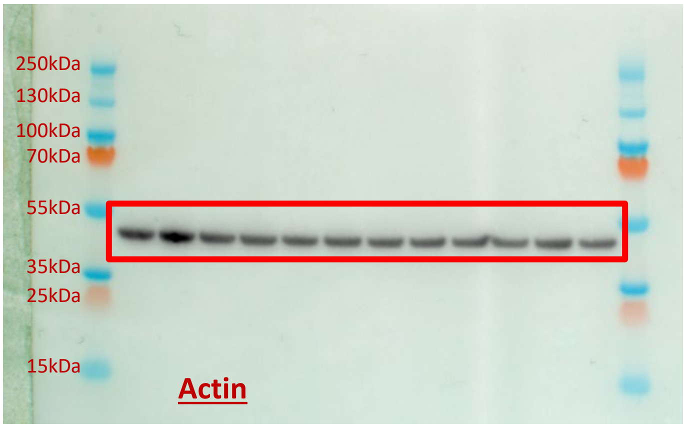

Fig S9e

Lu99

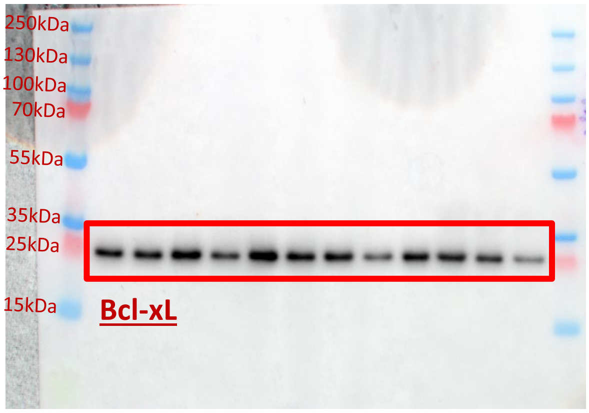
250kDa
130kDa
100kDa
70kDa
55kDa
35kDa
25kDa
15kDa
Bcl-xL

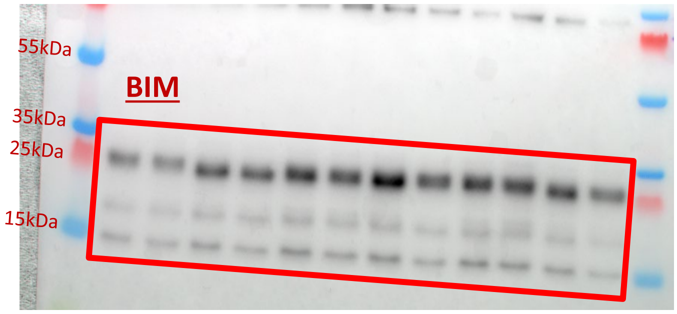
55kDa
BIM
35kDa
25kDa
15kDa

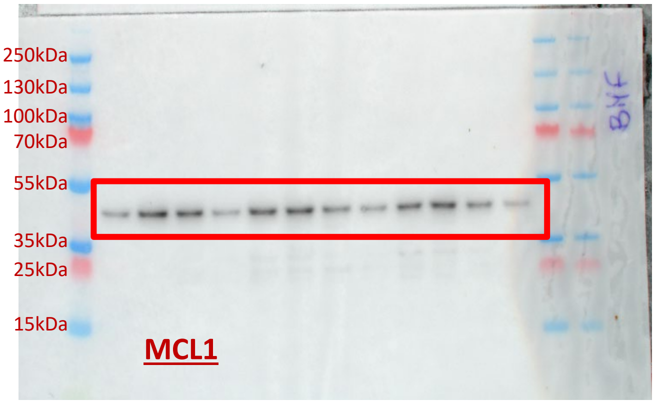
250kDa
130kDa
100kDa
70kDa
55kDa
35kDa
25kDa
15kDa
MCL1

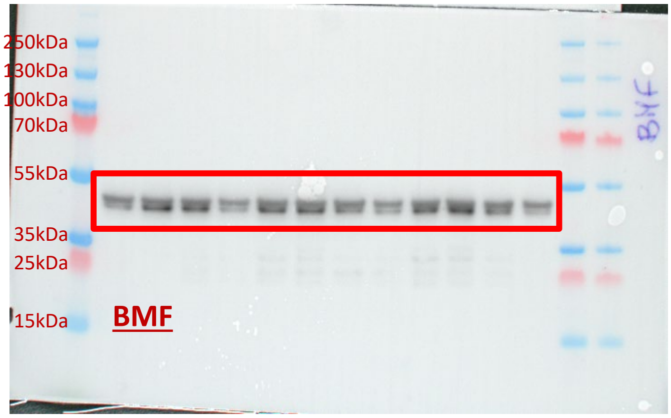
250kDa
130kDa
100kDa
70kDa
55kDa
35kDa
25kDa
15kDa
BMF

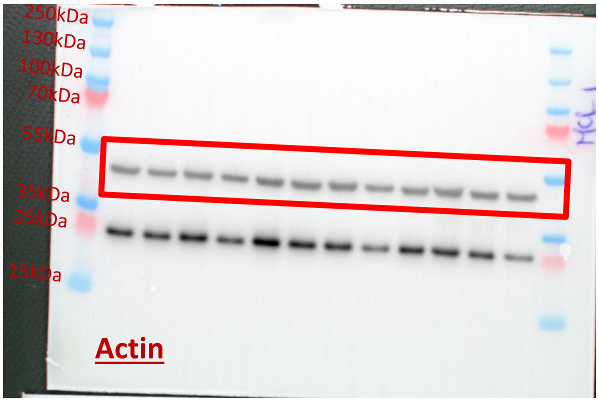
250kDa
130kDa
100kDa
70kDa
55kDa
35kDa
25kDa
15kDa
Actin

Fig S9e

NCI-H1792

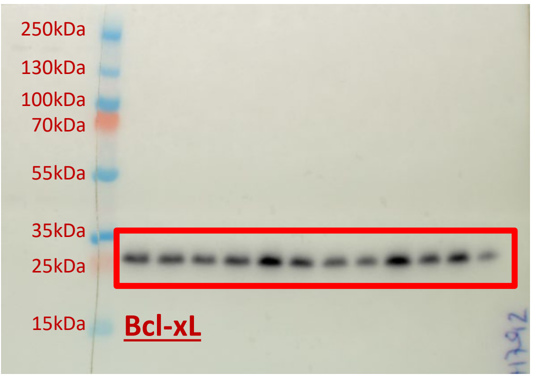

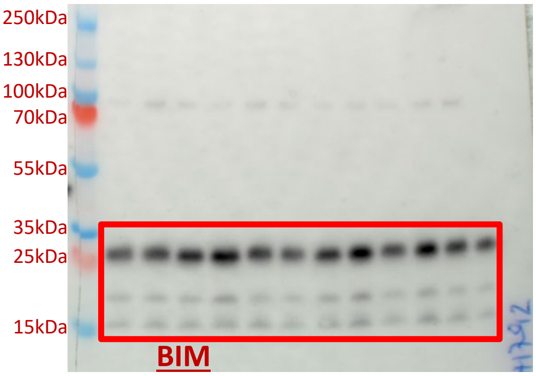

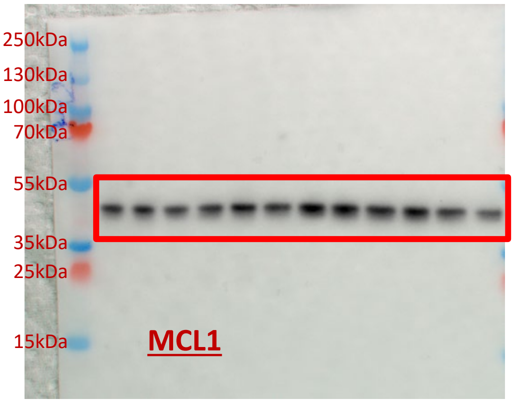

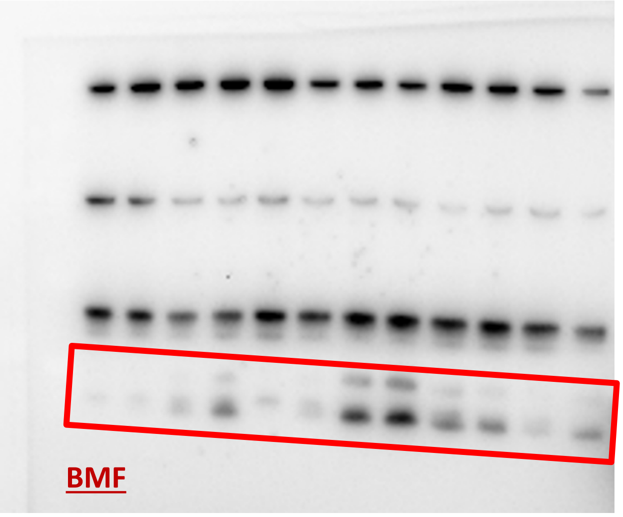

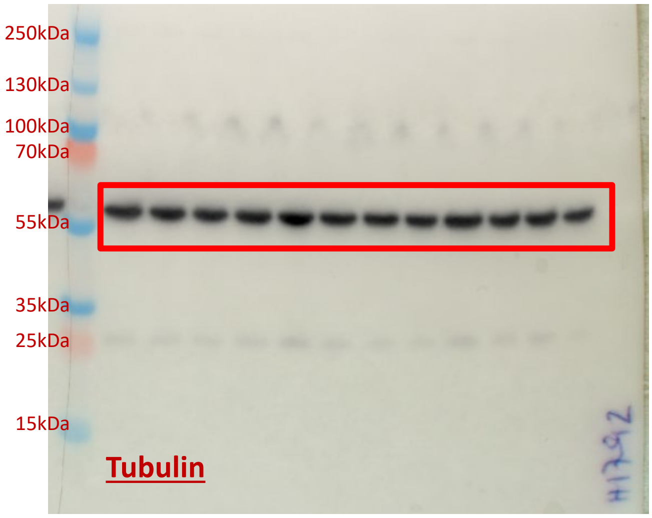

Supplement: Supplementary file 20 — Unprocessed western blots. [file 43018_2024_754_MOESM20_ESM.pdf]
